# Effects of aerobic exercise on cognitive function in older adults with mild cognitive impairment: a systematic review and meta-analysis

**DOI:** 10.3389/fpsyt.2025.1741998

**Published:** 2026-01-09

**Authors:** Qian Zhao, Zhao Chen Hong, Tian Tian Wei, Yu Ting Jiang, Hui Zeng, Guiling Miao

**Affiliations:** 1 Department of Respiratory and Critical Care Medicine, Nanjing Drum Tower Hospital, the Affiliated Hospital of Nanjing University Medical School, Nanjing, Jiangsu, China; 2 Xiangya School of Nursing of Central South University, Changsha, Hunan, China

**Keywords:** aerobic exercise, mild cognitive impairment, old adults, cognitive function, meta-analysis

## Abstract

**Background:**

Regular exercise may boost cognition in older adults with MCI, but the effectiveness of different aerobic exercise (AE) modalities is unclear. We conducted a meta-analysis to assess AE’s impact on cognition in this group.

**Objective:**

This systematic review and meta-analysis aimed to evaluate the effects of AE on cognitive function in older adults with MCI.

**Methods:**

We searched PubMed, Embase, Web of Science, and the Cochrane Library up to July 2025 and referenced included articles. RCTs with aerobic exercise and outcomes for global cognition and specific cognitive domains in older MCI adults were included. Data was pooled using mean difference, standardized random-effect model, and 95% CI with RevMan V.5.4 and Stata 16.0.

**Results:**

A total of 33 studies with 1996 participants were included. The results revealed that AE exerted a significant positive effect on global cognition (SMD = 0.81, 95% CI (0.50, 1.12), Z = 5.07, p=0.00) and a moderate positive effect on memory (SMD = 0.34, 95% CI (0.06, 0.63); Z = 2.33, p = 0.02). Notably, no significant improvements were observed in executive function, attention, processing speed, language, or visuospatial abilities. Furthermore, meta-regression and subgroup analyses indicated statistically significant variations across different intervention combinations, suggesting heterogeneity in treatment effects.

**Conclusion:**

AE significantly improved global cognition and memory in elderly individuals with MCI. Furthermore, the frequency (3–5 sessions per week), timing, intensity, and total duration of the interventions should be tailored to individual needs and gradually refined for personalized optimization.

**Systematic Review Registration:**

https://www.crd.york.ac.uk/prospero/, identifier CRD42021238308.

## Introduction

The accelerating aging of the global population has led to a significant increase in the risk of MCI among adults agedtss years, with prevalence rates rising sharply with advancing age. For instance, the prevalence is 6.7% in adults aged 60–64 years but rises dramatically to 25.2% among those aged 60 years and above (1, 2). Other studies have reported that approximately 15% of adults aged > 60 years are affected by MCI (3). Among those affected, individuals with amnestic MCI (aMCI), characterized by substantial memory decline, are at a significantly higher risk of developing Alzheimer’s disease(AD). In contrast, those with non-amnestic MCI (naMCI) primarily exhibit deficits in other cognitive domains, such as executive function and language (4). Compared to cognitively healthy peers, individuals with MCI face a markedly elevated risk of dementia progression. The conversion rate for MCI patients aged 65 and above is approximately 14.9% within 2 years and rises to 23.8-46% within 3 years (4, 5). Although an estimated 10tima of individuals may experience cognitive stability or even improvement (6), the lack of effective, timely interventions poses a substantial socioeconomic burden, with associated costs projected to soar to US$2.54 trillion by 2030 (7). This escalating burden underscores the urgent need for effective prevention and treatment strategies in global public health initiatives (8–10). Given the limited efficacy and lack of consensus regarding pharmacological interventions (e.g. acetylcholinesterase inhibitors) (11, 12), non-pharmacological approaches, including cognitive training, dietary modifications, and social engagement, have emerged as primary strategies for mitigating cognitive decline (13, 14).

Among the array of non-pharmacological interventions for MCI, exercise has garnered significant attention for its cost-effectiveness and practicality (15, 16). Among the various exercise modalities, aerobic exercise (AE) offers distinct advantages owing to its unique neuroprotective mechanism. Research indicates that AE not only enhances cardiopulmonary fitness, increases cerebral blood flow, and promotes hippocampal neuroplasticity (17, 18) but also confers neuroprotective benefits via multiple pathways, including reducing amyloid-g deposition (19–21) and mitigating oxidative stress (22–24). Recent high-quality evidence reviews have further clarified the overall benefits of AE for individuals with MCI (25–27). Nevertheless, the specific effects of AE on cognitive function in older adults with MCI remain inconsistent. For example, a network meta-analysis identified moderate-intensity AE as a highly effective intervention for enhancing global cognition in this population (25). Similarly, an umbrella review affirmed the positive impact of exercise (including AE) on cognitive impairment but emphasized that significant heterogeneity in exercise type, dosage, and assessment tools contributes to inconsistent findings (27). Furthermore, research comparing different exercise modalities for alleviating neuropsychiatric symptoms in MCI underscores the importance of tailored intervention strategies (26). Therefore, this study aims to conduct a systematic review and meta-analysis to quantitatively evaluate the effects of AE on cognitive function in older adults with MCI. The specific objectives are: (1) to determine the overall efficacy of AE for improving cognition in MCI; (2) to explore, via subgroup analysis and meta-regression, the relationship between specific exercise parameters (e.g., frequency, intensity, duration) and cognitive outcomes; and (3) to provide an evidence-based foundation for developing standardized and feasible AE intervention protocols.

## Methods

The review protocol was registered with the International Prospective Register of Systematic Reviews (PROSPERO) (CRD42021238308).

### Literature research

For a more comprehensive literature search, two investigators independently searched PubMed, Embase, the Cochrane Central Register of Controlled Trials, and Web of Science for relevant literature from inception to July 2025. The search phrases used were: (“Cognition Disorders” or (“cogniti*” or “impair*”)) and (“exercise” or “physical activity” or “fitness” and (“Geriatrics” or “Aged”). Hand searching was also performed to identify additional relevant publications from the reference lists of all articles. The complete search strategy is presented in **Table 1**.

**Table 1:** Search strategy.

PICO Component	#	Search strategy
intervention	1	“Physical Education and Training” [Mesh] or “Sports” [Mesh] or “complementary therapies” [Mesh] or “exercise” [Mesh] or “exercise therapy” [Mesh] or “physical fitness” [Mesh]
	2	(physical* OR training OR exercise*) ti,ab or (yoga OR tai chi OR tai ji OR walking OR running OR jogging OR swimming OR cycling OR bicycling OR qigong OR chi kung) ti,ab or aerobic* ti,ab or fitness ti,ab
	3	#1 or #2
population	4	“Cognition Disorders” [MeSH]
	5	((cognit* OR memory OR cerebr* OR mental*) AND (declin* OR impair* OR loss* OR deteriorat* OR degenerat* OR complain* OR disturb* OR disorder*)). ti,ab or (MCI OR AAMI OR ACMI OR ARCD OR CIND OR SMC OR BSF OR MD OR LCD OR QD OR AACD OR MNCD OR MCD OR aMCI OR MCIa) or (“CDR” OR “clinical dementia rating scale” OR “GDS” OR “global deterioration scale”) ti,ab or (“preclinical AD” OR “pre‐clinical AD” OR “preclinical alzheimer*” OR “pre‐clinical alzheimer*”) ti,ab or (“Benign senescent forgetfulness” OR “mild neurocognitive disorder*”) ti,ab or (prodrom* AND dement*) or (episodic* AND memory)
	6	#4 or #5
	7	Aged [MeSH] OR Geriatrics [MeSH] OR Aging[MeSH]
	8	(Old OR older* OR Elder* OR geriatr* OR senior* OR “late life” OR ageing OR aging OR aged) ti,ab
	9	#7 or #8
study design	10	(randomized controlled trial[Publication Type] OR controlled clinical trial[Publication Type] OR randomized[Title/Abstract] OR placebo[Title/Abstract] OR clinical trials as topic [Mesh: NoExp] OR randomly[Title/Abstract] OR trial[Title]) NOT (animals [Mesh] NOT (humans [Mesh] AND animals[Mesh]))
	11	#3 AND #6 AND #9 AND #10

### Inclusion and exclusion criteria

Studies that met the following criteria were included in the systematic review and meta-analysis: (1) Participants: older adults aged 60 years and above who met the diagnostic criteria for MCI, Individuals aged ≥ 60 years are considered older according to WHO (28). (2) Interventions: Aerobic exercise (29), also referred to as endurance activities, entails rhythmic movement of the body’s large muscle groups over a sustained duration. This form of exercise is renowned for enhancing cardiovascular and pulmonary health and includes popular activities such as walking, running, swimming, and cycling. (3) Any type of control group was eligible, including no treatment, a waiting list, or an alternative active treatment group. (4) Outcome measurements involving global cognition, memory (immediate and delayed recall), executive function, attention, language, and visuospatial function; (5) Randomized controlled trials (RCTs).

Conference abstracts, case reports, commentary articles or letters to the editor, and protocols were excluded. Studies including elderly patients with vascular cognitive impairment or other neurological disorders resulting from AD, dementia, Parkinson’s disease, stroke, cardiovascular disease, or other severe illnesses were also excluded. In addition, when duplicate or overlapping data were found in multiple reports, only the report with the most complete information was included.

### Data extraction

Two independent authors (ZQ and HZC) evaluated the eligible studies and extracted data using a self-designed information extraction form. Disagreements were resolved by consensus or by the judgment of a third reviewer (ZH). The following information was extracted from each study: author, country, study type, participants (age, sex, sample size, and percentage), intervention (type, duration, frequency, and intensity), and outcome. We extracted the means and standard deviations to compute effect sizes. The direction of the effect size was adjusted such that a positive effect size indicated an improvement in the outcome measures.

### Risk of bias assessment

Two independent reviewers (ZQ and HZC) assessed the risk of bias of the included studies using the Cochrane Collaboration Risk of Bias Tool (30). The overall rating is determined by evaluating the six aspects of each study (Selection Bias, Performance Bias, Detection Bias, Attrition Bias, Reporting Bias, and other Biases) as low risk, high risk, and unclear. Disagreements between the two reviewers were resolved through discussions.

### Statistical analysis

All analyses were performed using Review Manager (RevMan 5.4) and Stata 16. Hedges’ adjusted g was used to calculate the effect sizes for the standardized mean difference (SMD). SMD was preferred over Mean Difference (MD) because of the use of different instruments to measure cognitive outcomes in the included studies. SMD standardizes results across diverse scales, enabling the comparison of effect sizes despite different measurement tools (31). This approach ensured a more consistent and interpretable summary of the intervention effects on cognitive function. SMD values of 0.15–0.40, 0.40–0.75, and over 0.75 were considered small, medium, and large, respectively (32). Random-effects models were used to pool the summary SMD. Tests for heterogeneity were conducted and assessed using I^2^ and Q statistics. An I^2^ of less than 25%, 25%–50%, and over 50% was usually viewed as low, moderate, and high heterogeneity, respectively (33). A p-value of < 0.05 for the Q statistic and I^2^  > 50% reflected significant heterogeneity. Egger’s tests and a contour-enhanced funnel plot were created to check for publication bias (34).

Additionally, a meta-regression analysis was performed to explore the sources of heterogeneity among studies and systematically evaluate the effects of potential moderator variables on the research outcomes (35). Subsequently, subgroup meta-analyses were performed based on the moderator variables identified as significant in the meta-regression analysis. This approach facilitates a deeper exploration and elucidation of the influence of these moderator variables on effect sizes within more homogeneous subsets (36). These analyses assessed the moderating effects of intervention frequency, duration, intensity, and intervention (i.e. dance, bicycle, walking, Tai Chi) and control (i.e. usual care, placebo, health education, others) types.

## Results

### Search process

**Figure 1:**
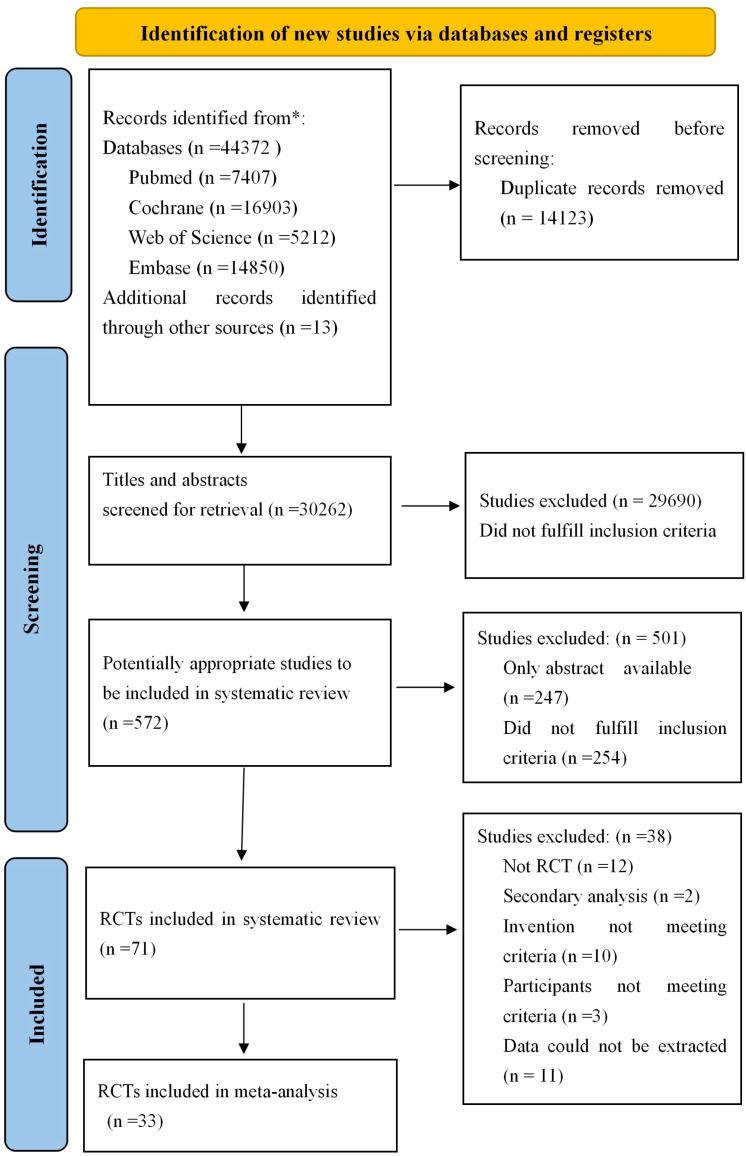
Flow diagram for searching and selection of the included studies. RCTs, Randomized controlled trials.

The results of the search are presented in **Figure 1**. A total of 44372 studies were identified from four databases, and 13 studies were obtained from citations related to references. A total of 14123 were removed because of duplication. After screening the titles and abstracts, 29690 studies were excluded, leaving 572 studies with full texts to be checked for eligibility. Of these, 247 were protocol/conference abstracts, 254 did not fulfill the inclusion criteria, 12 were non-RCTs, and two studies (37, 38) were secondary analyses from one study that produced more than one publication; thus, they were regarded as one study. The intervention method is multi-component intervention (39–48), three studies did not meet the inclusion criteria (49–51) and 11 were excluded due to insufficient data. Ultimately, 33 studies were included in this review.

### Study characteristics

The detailed characteristics of the 33 studies included in this analysis are listed in **Table 2**. A total of 33 RCTS, involving 1996 participants with MCI (men, 640; women, 1010; mean age 71.44) were included, of whom 1004 were allocated to the intervention groups and 992 to the control groups. All studies were published between 2008 and 2024. The study countries and regions included China (52–64)(n=13), USA (65, 66)(n=2), Spain (67, 68)(n=2), Turkey (69, 70)(n=2), Thailand (71, 72)(n=2), Korea (73)(n=1), Japan (74) (n=1), French (75) (n=1), Canada (76) (n=1), Brazil (77) (n=1), Greece (78) (n=1), Germany (79) (n=1), Dutch (80) (n=1), India (81) (n=1), Pakistan (82) (n=1), Iran (83) (n=1), Tunisia (84)(n=1). In terms of intervention characteristics, the type of aerobic exercise included walking (55, 63, 66, 69, 71, 77, 80, 81, 85) (n=9), treadmill or bicycle (53, 59, 65, 68, 75, 82) (n=6), dance (552, 56, 60, 64, 67, 74, 78) (n=7), pedaling exercise (84)(n=1) kayak (73) (n=1), Handball (62)(n=1), stepping (57) (n=3), Taichi (54, 72)(n=2) and Baduanjin (58)(n=1). The duration of aerobic exercise was, on average, 19 weeks (range =8–48 weeks) with 3 sessions per week (range =1–7 sessions) of 20–60 minutes in volume in the 33 studies, a heart rate reserve of 40–60%, 40–90% maximum heart rate, Rating of Perceived Exertion(RPE) 11-15, Borg scale12-14, or metabolic equivalents were applied to control the intensity of aerobic exercise in 25 studies included studies. Among the 33 articles, 16 studies clearly indicated the adoption of moderate-intensity exercise, two studies adopted low or extremely low-intensity exercise (68, 71), four studies adopted high-intensity exercise (65, 66, 79, 83), and one study used the intensity of exercise that varied with the duration of intervention (52, 55, 59, 67, 70, 76) (**Table 2**).

**Table 2:** Characteristics of included studies.

Author, year, country	Design	Setting	No of patients (Male/ Female)	Age (year)	Exp group			Control group	Outcome
					Frequency/Duration and intensity	Duration/follow-up	invention	Intervention	
Ayed,2024 (84) Tunisia	RCT	Laboratory	EG15 (5/10) CG14(2/12)	EG: 67.93 ± 5.18 CG: 69.24 ± 4.84	20-min/day, twice-weekly/moderate intensity (60% HRmax)	8 weeks/1 months	Aerobic (pedaling exercise):	Reading for 20 minutes	MMSE, RL/RI-16, Letter fluency, Category fluency, Clock test, Stroop, Tower of Hanoi, DST
			EG15 (5/10)	EG: 67.13 ± 3.04	20-min/day, twice-weekly/moderate intensity (60% HRmax)	8 weeks/1 months	Aerobic combined with cognitive (ACT)		
Baker,2010 (65) USA	RCT	clinical research unit	EG23 (13/10) CG10(5/5)	EG: 67.95 ± 8.51 CG: 72.6 ± 8.7	45–60 min/day, 4 days/week, high-intensity (75-85% HRmax)	6months	Aerobic (treadmill, stationary bicycle, or elliptical trainer)	Stretching exercise	TMT, SCWT, Task Switching, Verbal Fluency, SDMT, Story Recall, List Learning, visual memory
Bisbe,2020 (67) Spain	RCT	Hospital	EG17 (9/8) CG14(7/7)	EG: 72.88 ± 5.60 CG: 77.29 ± 5.16	60 min/day, 2 days/week, Low to Moderate intensity(NR)	12 weeks	Choreography (aerobic dances)	Physical Therapy (strength, endurance, flexibility, balance, coordination and gait)	WMS-III, RBANS, TMT-A, TMT-B, Letter fluency, Category fluency, Boston Naming Test, Judgment of Line Orientation
Bademli,2018 (69) Turkey	RCT	nursing home	EG30(12/18) CG30(13/17)	EG: 72.24 ± 7.16 CG: 70.67 ± 8.34	20 -40min, minimum 3 days/week, Moderate intensity (3–6 MET)	20 weeks	Aerobic Exercise (rhythmic exercises + free walking)	NR	MMSE
Chang 2021 (52)China	single-blind RCT	nursing home	EG 62(NR) CG 47(NR)	EG: 76.56 ± 3.60 CG: 75.94 ± 3.61	30 min/times, 3 times/week, Moderate-high intensity(100-140/min HR)	18 weeks/	Aerobic Exercise (square dance)	NR	MOCA
Choi,2019 (73) Korea	RCT	A welfare center	EG 30 (5/25) CG 30(4/26)	EG: 77.27 ± 4.37 CG: 75.37 ± 3.97	60 min/day, 2 days/week, NR	6 weeks	Virtual kayak paddling exercise	Home exercise	MOCA
Doi,2017 (74) Japan	3 arms, single-blinded, RCT		EG 67 (33/34) CG 67(36/31)	EG: 75.7 ± 4.1 CG: 76.0 ± 4.9	60 min/week, NR, NR	40 weeks	Dance	Health education	story memory and word list memory tests
Donnezan,2018 (75) French	4 arms, RCT	Memory clinic	EG 18 (NR) CG 14(NR)	EG: 77.1 ± 1.44 CG: 75.37± 3.97	60 min/day, 2 days/week, Moderate intensity (60% HRmax)	12weeks/6 months	Aerobic exercise (bikes)	Usual lifestyle	The Matrix reasoning test, SCWT, DST-F, DST-B
			EG21 (NR)	EG: 67.13 ± 3.04	60 min/day, 2 days/week, Moderate intensity (60% HRmax)	12weeks	Combined simultaneous physical and cognitive training		
Karthikeyan,2022 (81)India	RCT	Community	EG 15(9/6) CG 15(8/7)	EG: 64.86 ± 2.87 CG: 64.40 ± 2.66	40–50 min/day, 1 session/day, moderate intensity (50-70% HRmax and RPE of 11-14.)	8 weeks	aerobic exercise program i.e. walking	stretching exercises	MOCA
Khattak, 2022 (82) Pakistan	RCT	Physiotherapy Rehabilitation Center	EG 29 (NR) CG 30(NR)	62.49 ± 1.82	40–50 mins/day, 5 days/week, moderate intensity (RPE of 11-14)	6 weeks	aerobic program (walked on treadmill)	Routine Care	MMSE, MoCA, TMT-A, TMT-B.
Kohanpour,2017 (83) Iran	CT	NR	EG 10 (10/0) CG 10(10/0)	67.85 ± 3.89	21–39 min, 3 times a week, high intensity (75%-85% of HRmax)	12 weeks	Aerobic exercise	Placebo	MMSE
Krootnark, 2024 (71) Thailand	Single-blind RCT	Community	EG 30 (6/24) CG 30 (6/24)	EG: 68.60 ± 4.86 CG: 69.70 ± 5.55	35–65 min/day,5 days/week, low intensity (Borg scale ≤ 13)	3 months	Aerobic exercise(walk, march, step)	Usual daily activities	MoCA, TMT-A, TMT-B, SCWT, DST-B, DST-F, SDT
Langoni,2018 (77) Brazil	Single-blind RCT	Primary care units	EG 26 (6/20) CG 26 (6/20)	EG: 72.6 ± 7.8 CG: 71.9 ± 7.9	20–30 min/day, 2 days/week, moderate intensity (60–75% HRmax)	24 weeks	Strenthing training +aerobic exercise (walking)	No treatment	MMSE
Law,2019 (53) China	4 arms, single-blind RCT		EG 16 (8/8) CG 14 (5/9)	EG: 77.94 ± 6.11 CG: 71.9 ± 7.9	60 min/session, 1–2 session/week, Moderate-intensity(NR)	8 weeks	Stretching and aerobic exercise (structured whole body movement exercise, bicycle and arm ergometry)	Waitlist control group	the Neurobehavioral Cognitive Status Exam- ination (NCSE), CVVLT, TMT-A, TMT-B
Lazarou,2017 (78) Greece	Single-blind RCT	Alzheimer Association and Related disorders	EG 66 (13/53) CG 63(15/48)	EG: 77.94 ± 6.11 CG: 67.92 ± 9.47	60 min/day, 2 days/week, NR	40 weeks,	International Ballroom Dancing	No treatment	MMSE, RBMT; the Verbal Fluency F-A-S test (FAS); TMT-B; Rey Osterrieth Complex Figure Test copy and delay recall (ROCFT-copy and delayed recall); RAVLT; Test of Everyday Attention (TEA)
Liu,2022 (54) China	Single-blind RCT	Community	EG 17 (5/12) CG 17(6/11)	EG: 73.2± 6.3 CG: 73.4 ± 6.5	50 min, 3 times a week, Moderate intensity(RPE 12-14)	12 weeks	Aerobic (24 form Yang Style Tai Chi)	usual daily physical activities	MoCA, TMT, California Verbal Learning Test (CCVLT), SCWT, The spatial n-back task test
Nagamatsu,2013 (55) China-	Single-blind,RCT	Community	EG 30 (NR) CG 28(NR)	EG: 75.6 ± 3.6 CG: 75.1 ± 3.6	60 min/day, 2 days/week, Low-Moderate intensity (40%-70–80% HRmax)	26 weeks	Aerobic (walking)	Balance and Tone	RAVLT, Spatial Memory
Qi,2019 (56) China	RCT	Dementia clinic and community	EG 16 (5/11) CG 16(4/12)	EG: 70.6 ± 6.2 CG: 69.1 ± 8.1	35 min/day, 3 days/week, Moderate-intensity (60–80% HRmax)	3 months	Aerobic exercise(dance)	Usual care	MMSE,MOCA,WMS-R LM, DST-F,B;TMT-A,B;SDMT
Song,2019 (57) China	RCT	Community	EG 60 (12/48) CG 60(18/42)	EG: 76.22± 5.76 CG: 75.33 ± 6.78	60 min/session, 3 times/week, Moderate-intensity(Borg scale 12-14)	16 weeks	aerobic stepping exercise	health education	MoCA
Sungkarat 2018 (72) Thailand	RCT	community.	EG 33 (2/31) CG 33(7/26)	EG: 68.3 ± 6.7 CG: 67.5 ± 7.3	50 min/session, 3 times/week, NR	6-month	Aerobic Exercise (Tai Chi)	routine lifestyle	WMS-LM, the Block Design Test, DST-F, DST-B;TMT-A, B
Stuckenschneider,2021 (85)Germany	RCT	three centers.	EG 60 (32/28) CG 58(23/35)	EG: 70.6 ± 6.1 CG: 71.6 ± 6.9	45 min/session, 3 times/week, High intensity (RPE≥13)	12 months	Aerobic Exercise (walking- running)	NR	a computer-based CogState Battery (International Shopping List Task-immediate and delayed recall, Detection Task, Identification Task, One Back Task, and One Card Learning Task), Verbal fluency, TMT.
Tao,2019 (58) China	RCT		EG 20 (5/15) CG 20(6/14)	EG: 66.17 ± 4.17 CG: 65.97 ± 5.66	60 min/day, 3 days/week, NR	24 weeks	Baduanjin group	health education	MOCA
			EG 17 (7/10)	EG: 64.32 ± 2.60	60 min/day, 3 days/week, Moderate-intensity(55-75% HRmax)	24 weeks	Brisk walking		
Thomas,2020 (66)USA Including: Tarumi,2019 (37) USA	Single-blind,RCT Single-blind,RCT	Community Community	EG 15 (8/7) CG 15(8/7) EG 31 (12/19) CG 39(15/24)	EG: 66.4 ± 6.6 CG: 75.8 ± 6.1 EG: 64.0 ± 5.9 CG: 65.3 ± 6.6	25–30 min,30-35min, 30-40min;3 session,3–4 session,4–5 session in a week; high-intensity (75-85%,85-90% HRmax)	12 Months 6 months	Aerobic Exercise (walking)	stretching and toning groups	Logical Memory Delayed Recall; Word Interference Test-Color Naming; Word Interference Test-Inhibition; California Verbal Learning Test; Boston Naming Test; Clock Drawing Test 1 the California Verbal Learning Test-second edition (CVLT-II) and the Delis-Kaplan Executive Function System (D-KEFS);
TenBrinke,2015 (76) Canada	Single-blind,RCT	Community and a memory clinic	EG 14 (NR) CG 13(NR)	EG: 76.07 ± 3.43 CG: 75.46 ± 3.93	60 min/day, 2 days/week, Moderate-high intensity(RPE 13-15)	6 months	Aerobic training	Balance and tone programme	RAVLT, SCWT; TMT-A, TMT-B
Tsai,2019 (59) China	3 arms, RCT	Hospital	EG 19 (5/14) CG 18(5/13)	EG: 66 ± 7.68 CG: 65.17 ± 7	40 min/day, 3 days/week, Low-moderate intensity (50-60%, 70-75% HRmax)	16 weeks	Aerobic exercise(a bicycle ergometer or a motor-driven treadmill)	static stretching exercises	MMSE, and the digit span component of the Wechsler-IV Adult intelligence test
Uysal 2022 (70) Turkey	RCT	Physiotherapy and Rehabilitation	EG 12 (10/2) CG 12 (10/2)	EG: 73.5 ± 3.2 CG: 74.08± 7.82	30 min/session, 3 times/week, Low-moderate intensity (40-60%, 50-75% HRmax)	12 weeks	AG: Aerobic + Lower extremity strengthening	Solely Lower extremity strengthenin	MMSE
			EG 12 (10/2)	EG: 73.25 ± 2.01	3 sessions/week,50-75% HRmax		ADG: Aerobic + Dual-task training + Lower extremity strengthening		
Van Uffelen,2008 (80) Dutch	Double-blind,RCT	Community	EG 77 (37/40) CG 75(48/27)	EG: 75 ± 2.96 CG: 75 ± 2.82	60 min/day, 2 days/week, Moderate-intensity(NR)	12 months	Walking program (WP)	Placebo Activity program (PAP)	MMSE, Auditory Verbal Learning Test (AVLT); verbal fluency test (VFT); Digit Symbol Substitution Test (DSST); SCWT-A
Varela,2012 (68) Spain	RCT	Elder residential care homes	EG 17(NR) CG 15(NR)	EG: 79.24 ± 10.07 CG: 79.40 ± 6.72	30 min/day, 3 days/week, Extremely low intensity(40% HRR)	3 months/3 months	Cycling in a recumbent bike	Recreational activities(playing cards,reading newspapers, handicrafts)	/MMSE
			EG 16 (NR)	EG: 76.44 ± 11.38	30 min/day, 3 days/week, low intensity(60% HRR)	3 months	Cycling in a recumbent bike		
Wang,2020 (61) China	2 arms, RCT	Community	EG 57 (21/36) CG 54(22/32)	EG: 68.37 ± 5.27 CG: 68.24 ± 5.15	60 min/day, 3 days/week, Moderate-intensity(60-80% HRmax)	12 weeks/12 week	Health class and structured exercise(limbering-up exercise & upper and lower limbs exercise & relaxation exercise)	Health education	MOCA, The Purdue Pegboard test
Wang,2024 (60) China	RCT	nursing home	EG 30 (NR) CG 30(NR)	EG: NR CG: NR	40 min/session, 4 times/week,NR	12 weeks	Aerobic (square dance exercise)	usual lifestyle	MOCA, The Stroop task, The n-back test, The more-odd shifting test
Wei,2014 (62)China	RCT	Nursing home	EG 30 (23/7) CG 30(19/11)	EG: 66.73 ± 5.48 CG: 65.27 ± 4.63	30 min/day, 5 days/week, Moderate-intensity(60% HRmax)	6 months	Handball exercise	routine lifestyle	MMSE
Zhu,2018 (64) China	Single-blind, RCT	Dementia clinic	EG 29 (14/15) CG 31(10/21)	EG: 70.3 ± 6.7 CG: 69.0 ± 7.3	35 min/day, 3 days/week, Moderate-intensity(60-80% HRmax)	3 months/3 months	Dance and usual care	Usual care	MOCA, The logical memory (LM) subtest of the Wechsler Memory Scale-Revised (WMS-R); SDMT; TMT, DST-F,DST-B
Zhang,2023 (63)China	RCT	Community	EG 14 (1/13) CG 14(1/13)	EG: 66.22 ± 5.51 CG: 69.75 ± 7.02	60 min/sessions, 3 times/week, Moderate-intensity(60% HRmax)	12 weeks	WG: a 60-minute 3 km walk	health education	MOCA, MMSE, SDMT
			EG 14 (2/12)	EG: 66.22 ± 5.51	60 min/sessions, 3 times/week, 60% HRmax	12 weeks	TCE + RTG: stretching, aerobic exercise, acupoint massage, and a “close your eyes and adjust your breath” exercise		

NR, no report; EG, exp group; CG, control group; RCTs, randomized controlled trials; HRmax, Maximum Heart Rate; RPE, The rate of perceived exertion; MOCA, Montreal Cognitive Assessment; MMSE, The Mini-Mental State Examination; ADAS-Cog, Alzheimer’s Disease Assessment Scale—Cognitive subscale; TMT-A, Trail Making Test A, TMT-B, Trail Making Test B; CVFT, category verbal fluency test; DST-F, Digit Span forward, DST-B, Digit Span Backward; SDMT, the Symbol Digit Modalities Test; RAVLT, the Rey Auditory Verbal Learning Test; SCWT, the Stroop Color and Word Test; SCWT-A, Abridged Stroop Colour Word Test; CVVLT, Chinese Version Verbal Learning Test; RBMT, Rivermead Behavioral Memory Test; RL/RI-16, the 16-item free and cued recall task; WMS-LM, the Logical Memory (LM) delayed recall, a subtest of the Wechsler Memory Scale; WMS-R, the Wechsler Memory Scale-Revised; WMS-III, Word List Learning test from the Wechsler Memory Scale-Third Edition; RBANS, the Visual Memory subtest of the Repeatable Battery for the Assessment of Neuropsychological Status; MIC, Memory Inventory for Chinese.

### Risk of bias

**Figure 2:**
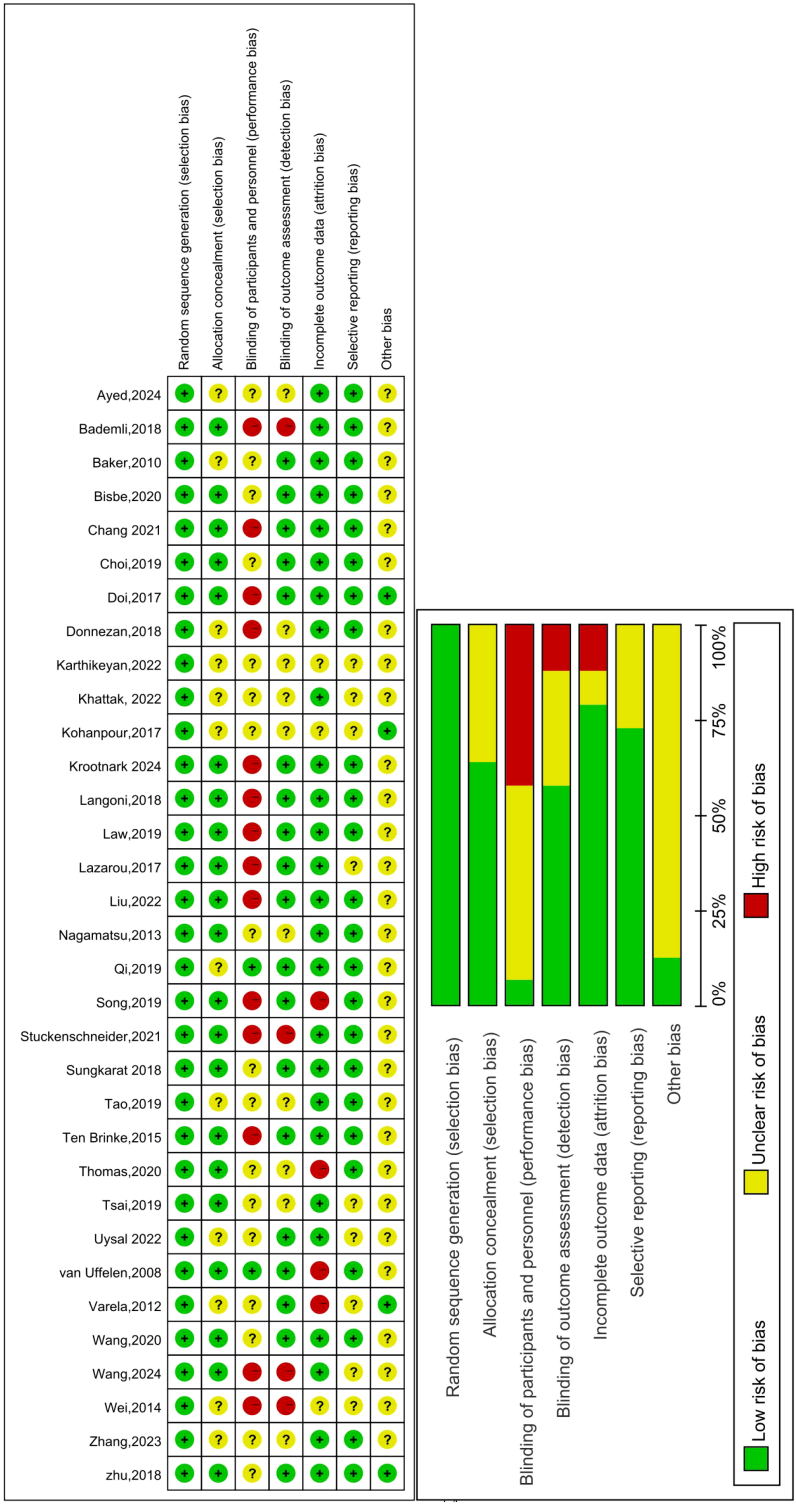
Risk of bias summary: Review authors’ judgements about each risk of bias item for each included study. Green circle: The risk of bias was low. Red circle: The risk of bias was high. Yellow circle: The risk of bias was unclear.

We conducted a risk of bias assessment for the included studies. The risk of bias and specific percentages (high, unclear, or low risk) are shown in **Figure 2**. Regarding the selection bias, all studies included reported random allocation, 21 studies of them reported the specific method of generated random number using computer (53, 55, 57, 61, 64, 71, 72, 74, 79), software(SPSS, Stata, Excel, SAS) (60, 66, 67, 73, 77, 78, 80), draw lots (52), URL (69, 76). Only two studies clearly indicated the method of allocation concealment using sealed and opaque envelopes (54, 59). Due to the type of intervention, it is difficult to blind the participants; therefore, the performance bias is high, and only two studies blinded the participants (56, 80). Nineteen studies adopted blinding to outcome evaluators (52–54, 56, 57, 61, 64, 65, 67, 68, 70–74, 76–78, 80), so the detection bias was low. In the attrition bias, studies were considered high risk since the dropout rate was higher than 20% (57, 66, 68, 80). We considered the reporting bias were low risk, since all the outcomes were reported, except in some studies that lacked research registration information.

### Effects of aerobic exercise intervention on MCI

#### Primary outcome: global cognition

**Figure 3:**
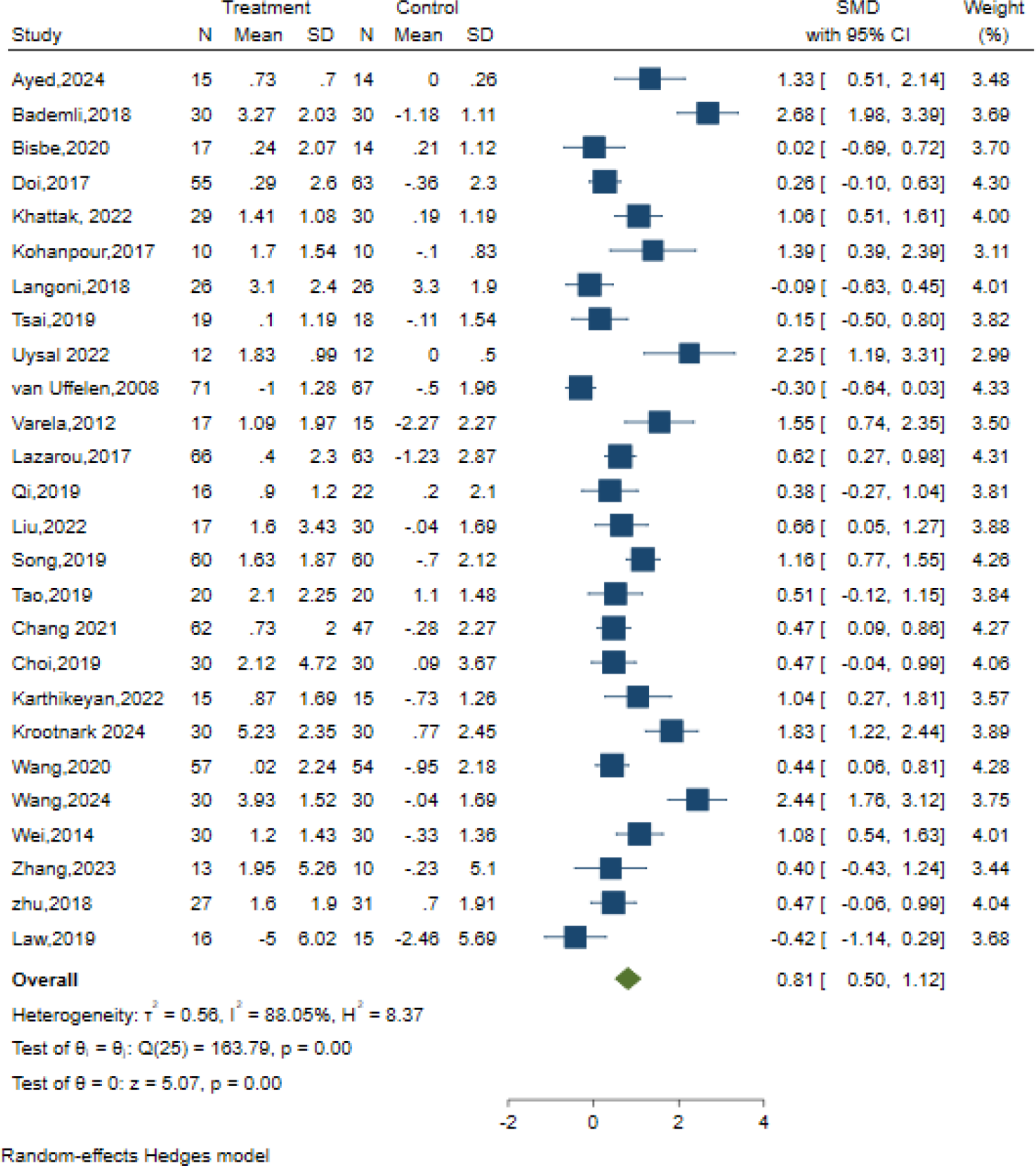
Forest plot for aerobic exercise on global cognition.

26 studies examined the effects of AE on global cognition using different classic and sensitive tests, including the MMSE, MoCA, and the Neurobehavioral Cognitive Status Examination (NCSE). The intervention group presented substantial improvement in global cognition relative to the control condition, using a random effects model. [SMD = 0.81, 95% CI (0.50, 1.12), Z = 5.07, p=0.00; I^2^ = 88.05%, p<0.01] (**Figure 3**). The contour-enhanced funnel plot showed that no study was trimmed; however, Egger’s regression test (z = 2.35, p = 0.02) suggested a potential publication bias (**Table 3**, **Supplementary Figure S1**).

**Table 3:** Summary of the meta-analysis results.

Outcomes	N of trials	N of participants	Effect Size	Test of Heterogeneity	Publication Bias (Egger’s Test)
Global cognition	26	1576	SMD = 0.81, 95% CI (0.50, 1.12)	I^2^ =88.0; Q = 163.79, p = 0.00	z = 2.35, p = 0.02
Attention	13	1106	SMD = 0.11, 95% CI (-0.19, 0.41)	I2 = 82.93%, Q = 54.40, p=0.00	z = 0.68, p = 0.50
Executive function	34	2862	SMD = -0.32; 95% CI (−0.14, 078)	I2 = 95.55%, Q = 262.45, p=0.00	z = 2.05, p = 0.04
memory	37	2038	SMD = 0.34, 95% CI (0.05, 0.63)	I^2^ =90.07; Q = 134.45, p = 0.00	z =1.90, p = 0.06
Processing speed	9	501	SMD = -0.07, 95% CI (-0.41, 0.27)	I^2^ =70.11; Q = 24.85, p = 0.00	/
Visual-spatial construction	6	414	SMD = 0.74, 95% CI (-0.37, 1.86)	I^2^ =96.09; Q =73.47, p = 0.00	/
Lanaguage	8	337	SMD = 0.22, 95% CI (-0.18, 0.62)	I^2^ =65.3; Q = 18.62, p = 0.01	/

Additionally, the meta-regression analysis indicated that AE Duration (min) had a statistically significant impact on effect size. Subgroup analysis was performed to assess the duration of AE interventions, which demonstrated that a duration of ≥30-≤50 min [SMD = 1.00, 95% CI (0.45, 1.55)], > 50 min [SMD = 0.30, 95% CI (0.00, 0.61)], and change in exercise duration[SMD = 1.31, 95% CI (0.57, 2.04)] conferred cognitive outcomes in older adults with MCI, Among which the duration of exercise exhibited the most significant variation in effect size and demonstrated the most optimal cognitive enhancement for older adults with MCI (**Figure 3**). The remaining meta-regression analyses showed that none of the moderators had a statistically significant impact on effect size. Specifically, the AE period in weeks did not significantly impact global cognition (z=-1.47, p = 0.14); similarly, the frequency of sessions per week (z=1.92, p = 0.06), Intervention Intensity (z=-0.15, p = 0.88), intervention type (z=0.17, p = 0.86), control type(z=0.04, p = 0.97), and scale type(z=-0.33, p = 0.74) showed no significant effect on the results. (**Supplementary Table S1**).

Despite these factors, we performed subgroup analyses concerning the overall duration of the intervention, frequency, intensity, and type of intervention, as well as the nature of the control group. Our findings revealed that aerobic exercise lasting ≤12 weeks[SMD = 0.93, 95% CI (0.54, 1.32)] exhibited a more significant intervention impact than exercise lasting > 12 weeks[SMD = 0.64, 95% CI (0.11, 1.16)]. Regarding intervention frequency, optimal results were observed when the sessions were conducted 3–5 times weekly [SMD = 1.08, 95% CI (0.70, 1.46)]. Regarding intervention intensity, low-intensity interventions emerged as the most effective [SMD = 1.20, 95% CI (0.16, 2.25)]. Among the diverse intervention types, walking interventions [SMD = 0.93, 95% CI (0.15, 1.72)] were more beneficial than dance interventions [SMD = 0.65, 95% CI (0.06, 1.25)]. Additionally, within the control group categories, stretching exercises [SMD = 1.10, 95% CI (-0.09, 2.29)] displayed the most favorable outcomes (**Supplementary Figures S2A–E**).

#### Secondary outcomes: cognitive sub-domains

Apart from the effects on global cognition, most studies also examined the effects of AE on specific cognitive sub-domains, including executive functions, memory, attention, language, processing speed, and visuospatial function.

### Executive function

**Figure 4:**
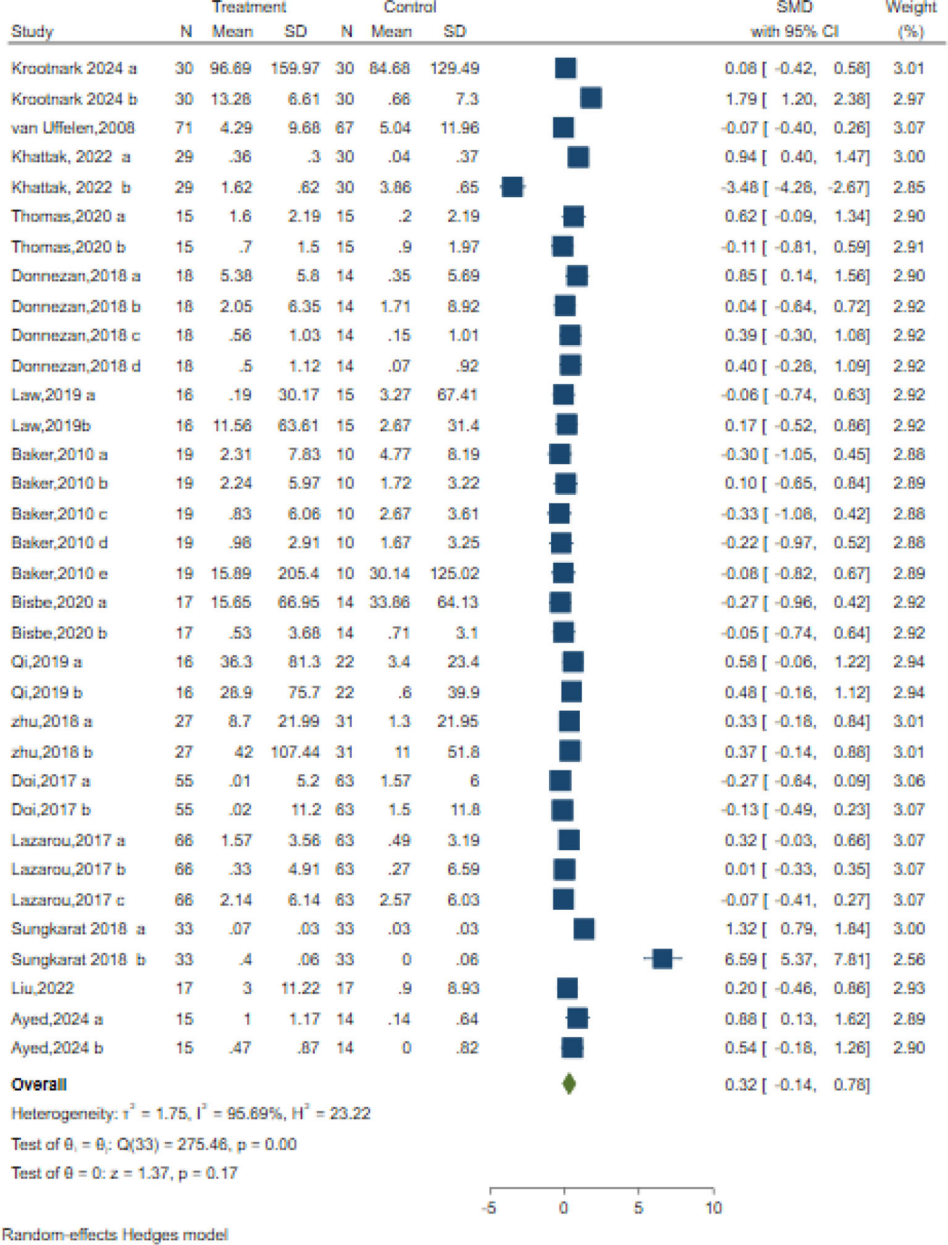
Forest plot for aerobic exercise on executive function.

Executive functions are a comprehensive cognitive domain, which includes functions like planning, problem solving, set shifting and inhibition (86) 34 controlled trials in 15 studies evaluated the effects on executive functions but used different classic and sensitive tests, including the Verbal Fluency Test (VFT), Stroop-Colour Test (SCT), Trail-Making Test (TMT), Matrix Reasoning test, Rey Osterrieth Complex Figure Test and Digit Span Test individually or in combination. A random-effects meta-analysis for executive function showed no significant changes in executive function [SMD = 0.32; 95% CI (−0.14, 078); Z = 1.37, p = 0.17; I^2^ = 95.69%, Q = 275.46, p=0.00] (**Figure 4**). Both Egger’s test (z = 2.05, p = 0.04) and contour-enhanced funnel plot suggested a potential risk of publication bias (**Table 3**, **Supplementary Figure S1**).

Additionally, the meta-regression analysis indicated that none of the moderators had a statistically significant impact on the effect size. Specifically, the AE period (z=-0.03, p = 0.97), duration of each session in minutes (z=-0.76, p = 0.45), frequency of sessions per week (z=-0.60, p = 0.55), Intervention Intensity (z=-1.32, p = 0.19), intervention type (z=1.66, p = 0.10), and control type(z=-1.02, p = 0.31) showed no statistically significant differences (**Supplementary Table S1**).

We also performed subgroup analyses concerning the overall length of the intervention, frequency, intensity, and type of intervention, as well as the type of control group. Our findings revealed that only the duration of intervention and the type of control group had a significant positive impact on executive function. Specifically, interventions lasting ≥30-≤50 min demonstrated the most prominent improvement in executive function[SMD = 1.37, 95% CI (-0.34, 3.08)]. Among control group types, interventions showed optimal efficacy when compared to usual care (SMD = 0.70, 95% CI: -0.30 - 1.71) or reading (SMD = 0.70, 95% CI: -0.19 -1.22). Given the inclusion of only two studies, this research suggests that interventions have the most significant effects when the control group receives usual care (**Supplementary Figures S3A–F**).

### Memory

**Figure 5:**
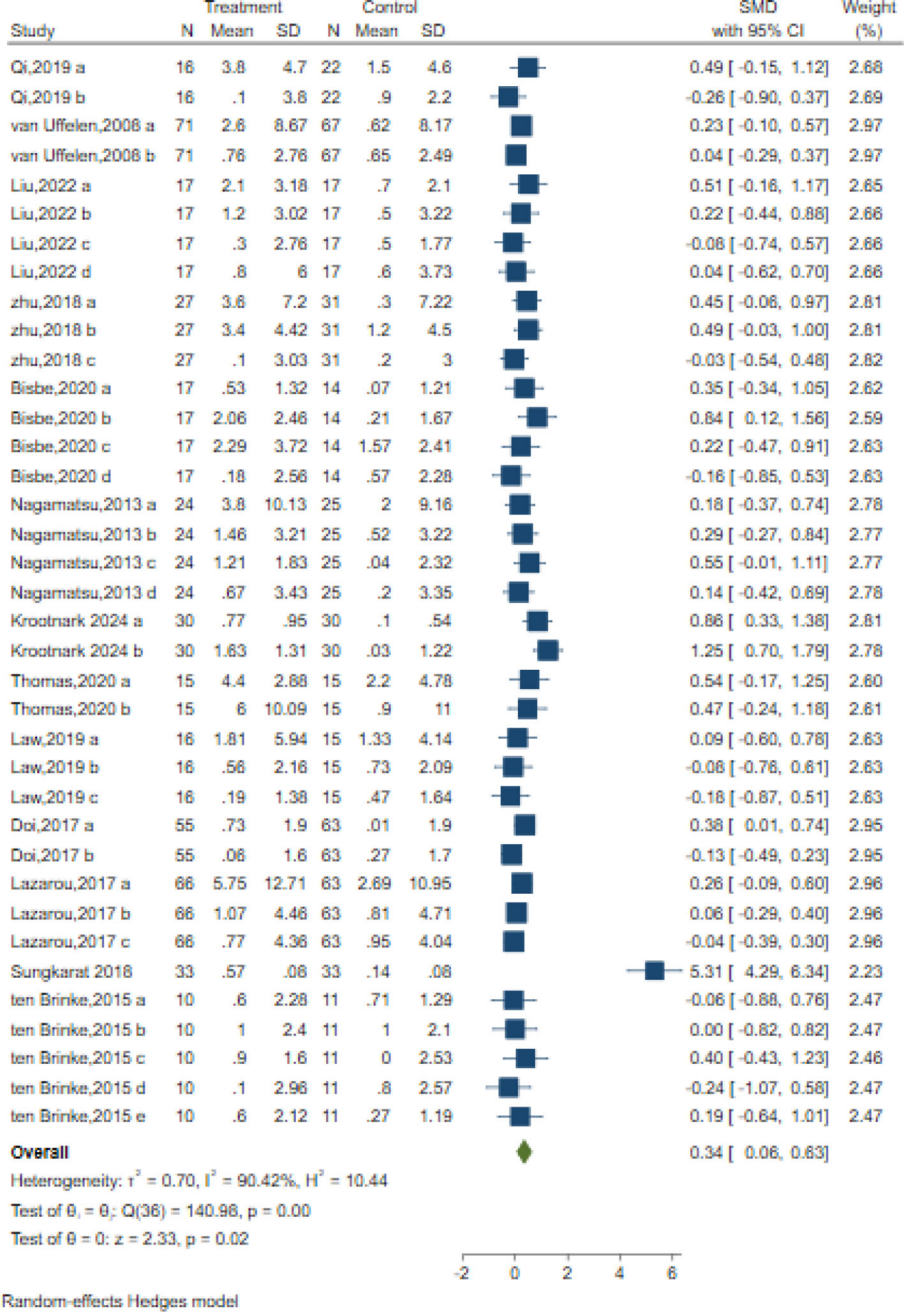
Forest plot for aerobic exercise on memory.

In total, 37 trials in 14 studies measured memory in older adults with MCI using different classic and sensitive tests, including the Auditory Verbal Learning Test (AVLT), Wechsler Memory Scale (WMS-II, WMS-III), Wechsler Memory Scale-Revised (WMS-R), Digit Span Test (DST-F, DST-B), spatial n-back task test, Visual Memory subtest of the Repeatable Battery for the Assessment of Neuropsychological Status (RBANS), and list learning delayed recall test. The results indicated that the intervention significantly improved memory with a moderate effect size compared to the control intervention [SMD = 0.34; 95% CI (0.06, 0.63); Z = 2.33, p = 0.02, I^2^ = 90.42%, Q = 140.98, p=0.00] (**Figure 5**). Among them,16 trials in six studies measured verbal memory. A small positive effect was reported after AE intervention compared to the control intervention [SMD = 0.11; 95% CI (-0.06, 0.28); Z = 1.27, p = 0.20, I^2^ = 0.29%, Q = 15.73, p=0.40] (**Figure 5**). Although Egger’s test indicated no evidence of publication bias (z = 1.90, p = 0.06), the results of the contour-enhanced funnel plot suggested that some studies were trimmed, implying a potential risk of publication bias (**Table 3**, **Supplementary Figure S1**).

The meta-regression analysis in **Supplementary Table S1** indicates that neither the intervention period (z=-0.18, p = 0.86) nor session frequency (z=1.53, p = 0.13) had a significant effect on memory. Additionally, duration (z=-0.58, p = 0.56) and intervention intensity (z=-1.23, p = 0.21) did not significantly impact the outcomes. Intervention types (z=1.61, p = 0.11) and control types (z=-0.90, p = 0.37) also did not differ significantly from no intervention (**Supplementary Table S1**). Overall, no significant moderators were identified for the memory.

We also performed subgroup analyses concerning the overall length of the intervention, duration, frequency, intensity, and type of intervention, as well as the type of control group. Our findings revealed that the overall length of the intervention, duration, frequency, intensity, and type of intervention all had a significant positive impact on memory. Specifically, the duration varied in exercise progress [SMD = 0.84, 95% CI (0.50, 1.17)], intervention frequency ≤ 3times/week [SMD = 0.14, 95% CI (0.03, 0.25)], intervention intensity varied in exercise progress [SMD = 0.23, 95% CI (0.06, 0.42)], and the over length ≤12 weeks[SMD = 0.27, 95% CI (0.10, 0.44)] demonstrated the most prominent improvement in memory. Among the intervention group types, walking interventions showed optimal efficacy (SMD = 0.42, 95% CI: 0.20 – 0.64). Similarly, given the current inclusion of only one or two studies in intensity, this research suggests that intervention intensity varies in exercise progress, exhibiting the most significant effects (**Supplementary Figures S4A–F**).

### Attention

**Figure 6:**
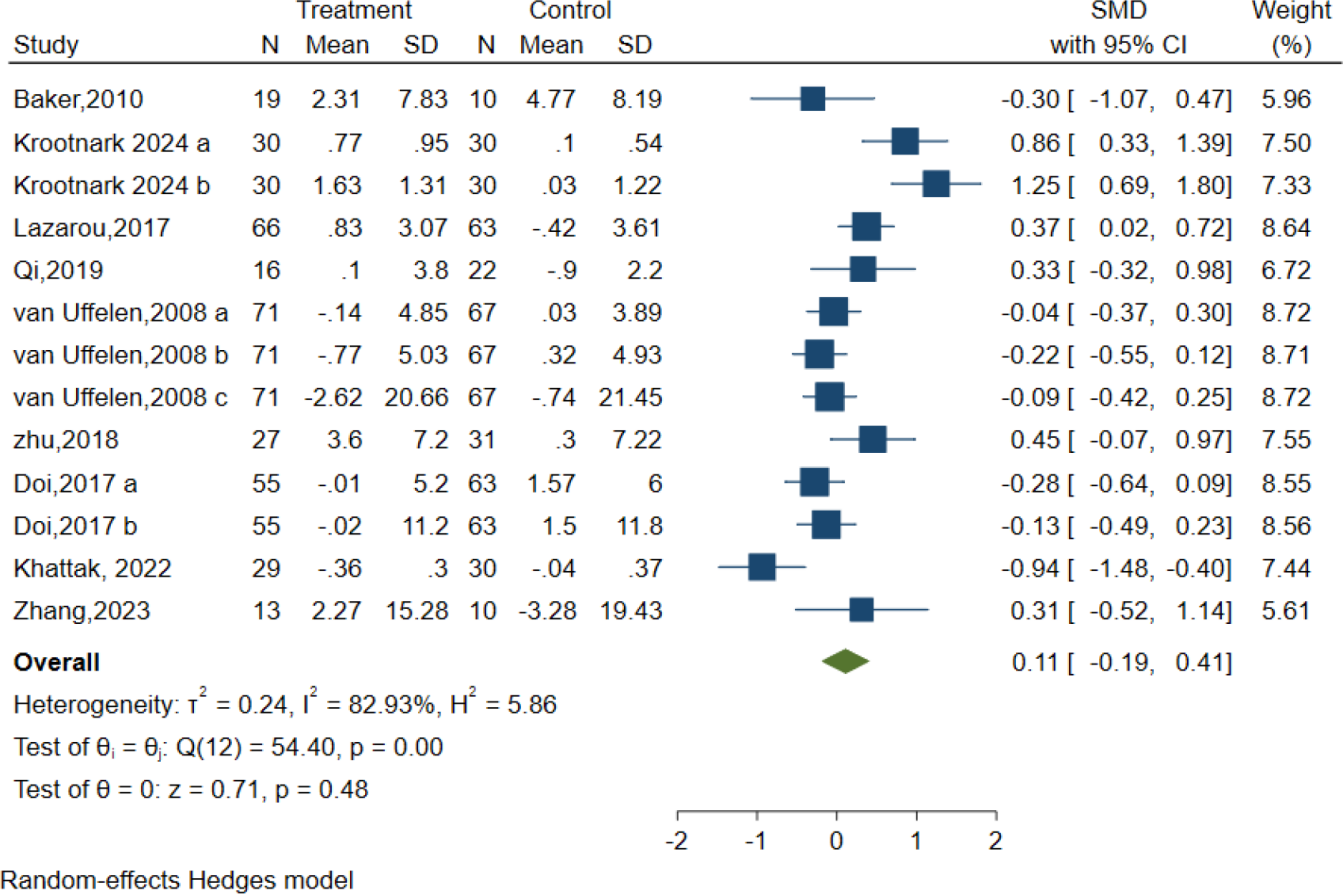
Forest plot for aerobic exercise on attention.

In total, 13 controlled trials in nine studies examined the impact of AE on attention using the Symbol Digit Modalities Test (SDMT), Digit Span Test (DST-F, DST-B), Test of Everyday Attention (TEA 4), and Abridged Stroop Colour Word Test (SCWT-A). Regardless of the assessments applied to assess the intervention effects on the attention domain. The intervention group presented no substantial improvement in attention relative to the control condition, as demonstrated by the random effect model [SMD = 0.11; 95% CI (-0.19, 0.41); Z = 0.71, p = 0.48, I^2^ = 82.93%, Q = 54.40, p=0.00] (**Figure 6**). The contour-enhanced funnel plot showed that no study was trimmed, and Egger’s regression test (z = 0.68, p = 0.50) suggested no potential publication bias (**Table 3**, **Supplementary Figure S1**).

Additionally, meta-regression analysis indicated that AE intensity had a minor statistical impact on effect size. A subgroup analysis was performed to assess the intensity of AE interventions, which demonstrated that moderate intensity conferred a very minor optimal attention in elderly individuals with MCI [SMD = -0.05, 95% CI (-0.40, 0.29), I^2^ = 75.14%, p=0.01] (**Figure 6**). The remaining meta-regression analyses showed that none of the moderators had a statistically significant impact on effect size. Specifically as follows: AE period in weeks did not significantly impact attention (z=-1.13, p = 0.26); Similarly, the duration of each session in minutes (z=-0.04, p = 0.96), frequency of sessions per week (z=0.66, p = 0.51), intervention type (z=0.03, p = 0.97) and control type(z=-1.43, p = 0.15) showed no significant effect on the results. (**Supplementary Table S1**).

Subgroup analyses were also conducted concerning the overall length of the intervention, frequency, type of intervention, and type of control group. Our findings revealed that none of these factors had a significant impact on attention (**Supplementary Figures S5A–E**).

### Processing speed

**Figure 7:**
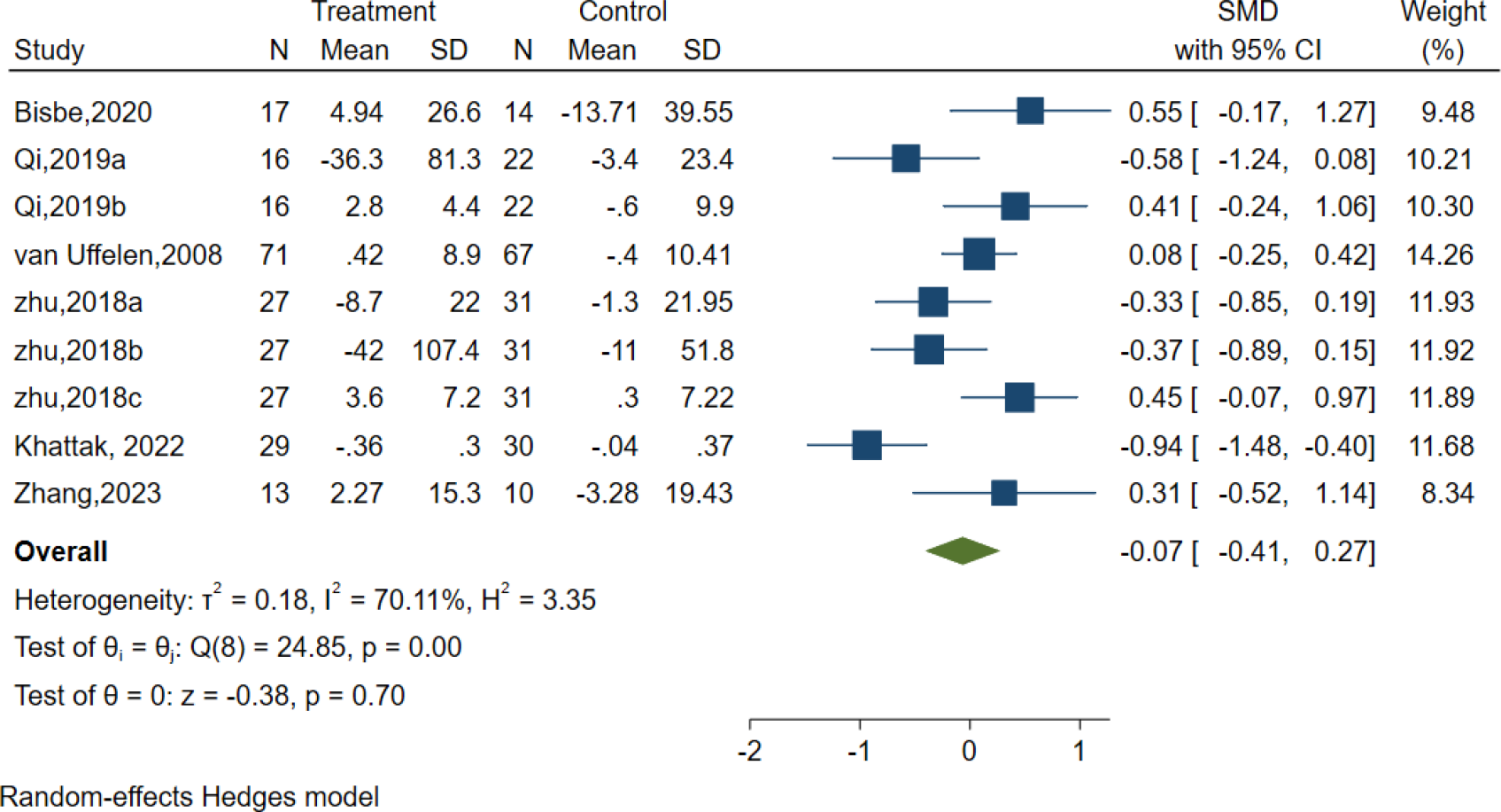
Forest plot for aerobic exercise on processing speed.

Six studies evaluated the effects of AE on Processing speed using different classic and sensitive tests, including the Symbol Digit Modalities Test (SDMT), Trail-Making Test (TMT), and Digit Symbol Substitution Test (DSST). However, the results indicated no significant effect of AE in the Processing speed [SMD =-0.07, 95% CI (−0.41, 0.27), p = 0.70; I^2^ = 70.11%, Q = 24.85, p = 0.00] (**Figure 7**).

Subgroup analyses were also conducted concerning the overall length of the intervention, duration, frequency, intensity, and type of intervention, as well as the type of the control group. Our findings revealed that none of these factors had a significant impact on attention (**Supplementary Figures S6A–F**).

### Language

**Figure 8:**
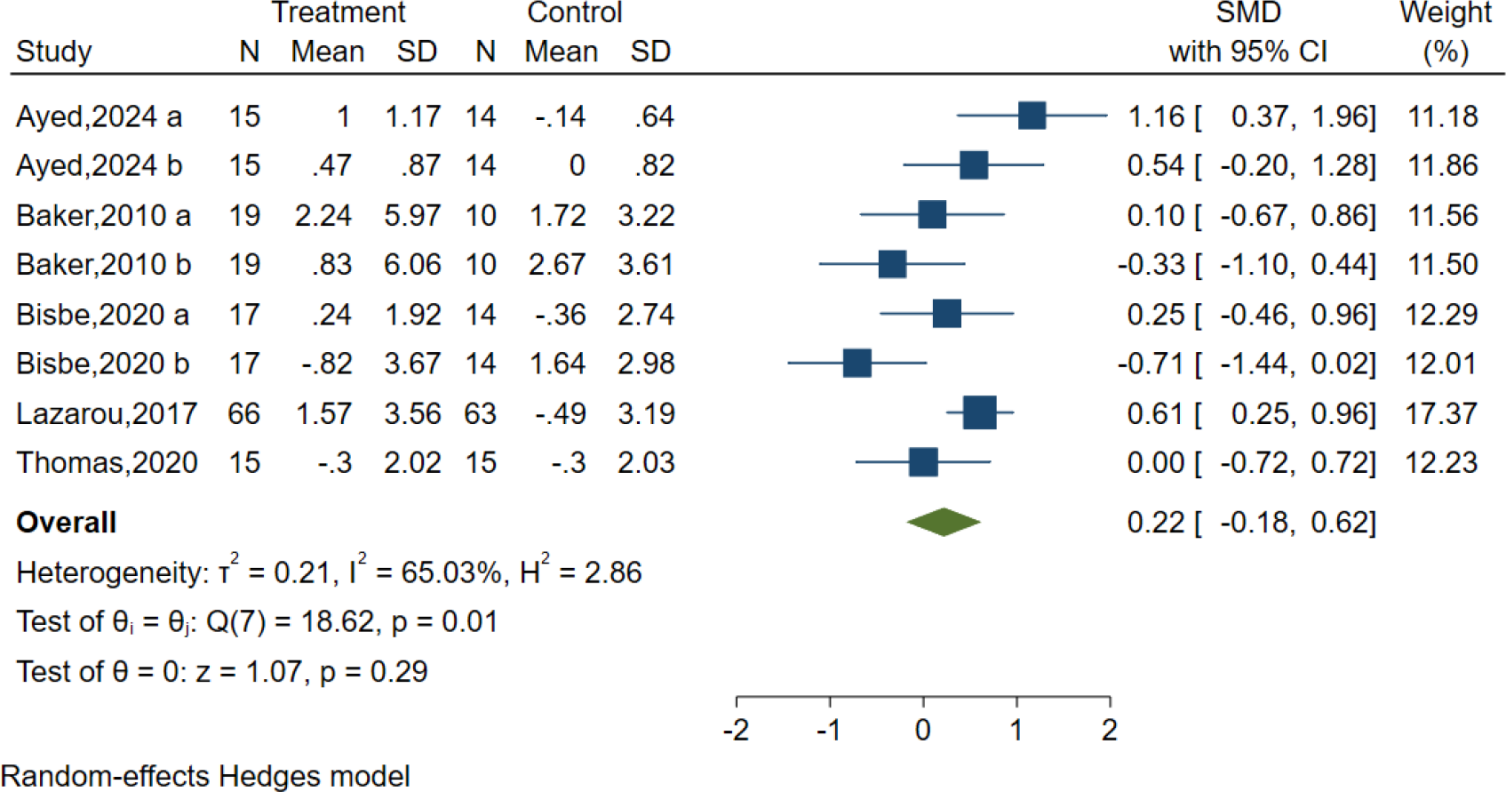
Forest plot for aerobic exercise on language.

Five studies evaluated the effects of AE on language using different classic and sensitive tests, including the Verbal Fluency Test (VFT), Verbal Fluency F-A-S test(FAS), and Boston Naming Test (BNT). However, the results indicated no significant effect of AE in the Language test [SMD = 0.22, 95% CI (−0.18, 0.62), p = 0.29; I^2^ = 65.03%, Q = 18.62, p = 0.01] (**Figure 8**).

We also performed subgroup analyses concerning the overall length of the intervention, duration, frequency, intensity, and type of intervention, as well as the type of control group. Our findings revealed that the intensity and type of control had a significant positive impact on language. Specifically, moderate-intensity [SMD = 0.84, 95% CI (0.23, 1.45)], and the control type was reading [SMD = 0.84, 95% CI (0.23, 1.45)] showed optimal efficacy. However, the results are not recommended for adoption because of the limited number of studies included, which may compromise their reliability and generalizability (**Supplementary Figures S7A–F**).

### Visuospatial function

**Figure 9:**
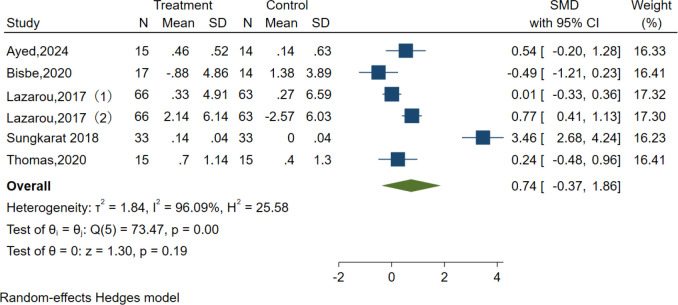
Forest plot for aerobic exercise on visuospatial function.

Five studies examined the effects of AE on visuospatial function using the clock test, Judgment of Line Orientation (JLO), and Rey-Osterrieth Complex Figure Test (ROCFT). However, the results indicated no significant effect of AE in the Visuospatial function test [SMD = 0.74, 95% CI (−0.37, 1.86), p = 0.19; I^2^ = 96.09%, Q = 73.47, p < 0.01] (**Figure 9**).

We also performed subgroup analyses concerning the overall length of the intervention, duration, frequency, intensity, and type of intervention, as well as the type of control group. Our findings revealed that the intensity and type of control had a significant positive impact on language. Specifically, the duration was ≥30–≤50 min [SMD = 3.46, 95% CI (2.70, 4.22)], the intervention type was Tai Chi [SMD = 3.46, 95% CI (2.70, 4.22)], and the control type was usual care [SMD = 3.46, 95% CI (2.70, 4.22)], which showed optimal efficacy. However, the results are not recommended for adoption due to the limited number of studies included, which may compromise their reliability and generalizability (**Supplementary Figures S8A–F**).

### Sensitivity analysis

We performed sensitivity analyses for both global cognition and specific cognitive domains. In each iteration, we removed one study at a time and assessed its impact on the overall effect size. Neither global cognition nor specific cognitive domains exhibited significant variations in effect size compared to the overall average, thereby validating the robustness of our research findings (**Supplementary Figure S9**).

## Discussion

We conducted a meta-analysis of global cognition and specific cognitive domains in older patients with MCI. Our analysis demonstrates that AE intervention has remarkable potential for enhancing cognitive function and significantly mitigating the conversion from MCI to dementia. Among elderly MCI patients, it elicits substantial improvement in global cognition, with domain-specific efficacy variations ranging from mild to moderate in the present study. The effect sizes of memory and attention were 0.34 and 0.15, respectively. There was no significant improvement in executive function, processing speed, language and visuospatial function. The meta-analysis did not detect significant enhancements in visual-spatial function, language, or processing speed following AE. This may be attributable to the limited sample size, as AE primarily engages large muscle groups and does not directly target the fine motor skills or hand-eye coordination mechanisms underlying the assessed cognitive domains.

In addition, we performed a dose-response meta-analysis to examine the impact of intervention frequency, duration, intensity, and cumulative duration on overall cognitive function and specific cognitive domains using subgroup analysis. Regarding intervention frequency, our results revealed that a regimen of 3–5 sessions per week elicited a large effect size, significantly enhancing both global cognition (ES = 1.08) and memory (ES = 0.70) in older adults with MCI. Sanders et al (86). engaging in AE at least four times a week elicited beneficial outcomes. Jia et al (87). demonstrated that working out three or more times weekly had a positive impact on enhancing cognitive function. These findings are largely consistent with our research, which involved older adults with dementia and included activities beyond aerobic exercise. Regarding intervention duration, our findings revealed that variations in intervention duration exhibited the most significant impact on overall cognition and memory in elderly individuals with MCI, with effect sizes of 1.31 and 0.84, respectively. Notably, for elderly MCI patients, executive function improvements achieved the largest effect size (1.37) when exercise sessions were tailored to a duration of 30–50 min. This aligns with the observations of Ahn et al (88), who similarly reported optimal cognitive benefits for elderly with MCI following 30–50 min of AE. However, contrasting results emerged from Sanders et al.’s study (86), which indicated peak efficacy at 30 min of exercise, with durations exceeding 45 min showing no significant effects. It is important to note that their study included patients with dementia and diverse aerobic modalities, suggesting that prolonged exercise may induce central fatigue, a phenomenon potentially linked to diminished cognitive outcomes. Supporting this, existing research has demonstrated that exercise exceeding 50 min can lead to reduced cerebral blood flow, thereby compromising neurological function (89). Uncertainty persists regarding the role of specific time intervals in triggering fatigue in humans. In terms of intervention intensity, the results of this study demonstrated that low-intensity AE exerted the most significant impact on the overall cognitive function of elderly patients with MCI (ES = 1.20), while fluctuations in intensity during exercise duration exhibited the greatest influence on memory performance (ES = 0.23). Furthermore, this study highlights that despite intervention intensity being a source of heterogeneity in attention outcomes, moderate-intensity aerobic exercise has a minimal effect on attentional abilities in elderly individuals with MCI(ES=-0.05). Conversely, the existing literature suggests that moderate-intensity AE may confer optimal overall cognitive benefits in the elderly (90). This observation could be attributed to the physiological decline in older adults, which limits their capacity to sustain higher-intensity workouts. Consistent with this, prior meta-analyses have indicated that chronic exercise with varied intensity loads differentially affects working memory, while other studies have elucidated that the beneficial effects of physical activity vary contingent upon exercise modality (91, 92). Regarding the length of intervention, this study revealed that interventions lasting ≤12 weeks yielded an overall cognitive function effect size of 0.93 and a memory effect size of 0.27. Notwithstanding, prior literature has suggested that 3–6 month interventions may elicit optimal effects on global cognition and diverse cognitive domains in elderly populations (90). However, the current study exclusively focused on healthy older adults and alternative aerobic exercise modalities only. Conversely, the ACSM recommends that older individuals should sustain exercise volumes for 16 or 32 weeks or longer to preserve and potentiate exercise benefits. Notably, the present meta-analysis demonstrated statistical significance exclusively for interventions ≤12 weeks in duration. Based on these findings, this study advocates a weekly frequency of 3–5 intervention sessions for older adult patients with MCI. Furthermore, the duration and intensity of individual sessions, along with the overall intervention timeline, should be progressively titrated to optimise efficacy.

Extensive research has demonstrated that AE significantly enhances cognitive ability through various mechanisms. Silva et al. (93) revealed that AE effectively promotes neural plasticity, boosts cerebral blood flow, and strengthens synaptic connectivity, thereby positively influencing cognitive function. From an evolutionary neuroscience perspective, Raichlen and Alexander (94) postulated that AE enhances brain adaptability, facilitating the maintenance of executive function and memory retention while improving cognitive resilience during aging. At the molecular level, AE modulates dopamine, a neurotransmitter pivotal in motivation and cognitive processing, the regulation of which by exercise may underpin its cognitive benefits. Stillman et al (95). Further, aerobic exercise delays cognitive decline through three interconnected mechanisms: at the cellular level, exercise promotes the secretion of neurotrophic factors (BDNF, NGF, IGF-1), which facilitate neuronal growth, synaptic plasticity, and long-term potentiation (LTP)—fundamental processes underpinning learning and memory (96, 97); at the neurostructural level, aerobic exercise induces structural remodelling, characterised by expanded gray and white matter volumes in the hippocampus and increased cortical thickness, alterations that are robustly associated with advanced cognitive functions. Psychologically, regular exercise alleviates stress, depression, and anxiety, enhancing emotional well-being and fostering cognitive resilience.

### Strengths and limitations

This study had several limitations. First, the use of multiple assessment tools to measure the same cognitive domain for comparison with AE interventions complicates the interpretation of the meta-analysis results. Second, the lack of standardized intervention protocols is a significant limitation. The “AE” interventions in the literature vary widely in modality (e.g., brisk walking, cycling), frequency, session duration, and intensity, lacking a unified, validated implementation framework. Third, considerable clinical heterogeneity across study samples must be acknowledged. Participants differed significantly in comorbidities, medication use, baseline physical and psychological status, sex, and education. Fourth, intervention durations were generally brief (predominantly 8–24 weeks), with a notable absence of long-term follow-up assessments. This precludes an evaluation of the long-term maintenance of cognitive benefits or the potential of AE to delay progression to dementia; future high-quality RCTs should therefore incorporate extended follow-up periods (e.g., 6–12 months post-intervention) to robustly validate the sustained benefits of AE.

The main advantage of this meta-analysis is the inclusion of a relatively large number of studies that adhere to strict and robust methodological standards to evaluate the efficacy of AE in enhancing cognitive function in elderly patients with MCI. The results show that AE can effectively improve the global cognition and memory of elderly people with MCI, and this result may provide empirical data for clinicians. However, methodological limitations and a limited number of studies have hindered the ability to determine the potential advantages of AE interventions for specific cognitive domains and different intervention methods. Furthermore, our review suggests future research should prioritize the following directions: First, researchers should develop a core, standardized AE intervention protocol for individuals with MCI. This requires establishing an evidence-based consensus on modality, intensity, frequency, and duration, supported by objective dose monitoring (e.g., using wearable devices). Second, multi-center collaborations should be established to employ a harmonized battery of validated cognitive assessments. This will enable robust longitudinal tracking of specific domains, particularly memory and executive function. Third, future study designs must systematically document comprehensive baseline participant characteristics (including MCI subtype, comorbidities, medication history, and genetic risk profiles) and incorporate post-intervention follow-up of at least 6–12 months. This is essential to clarify the long-term maintenance of cognitive benefits and the potential of AE to delay dementia progression. Ultimately, through the execution of high-quality, large-sample RCTs with extended follow-up, combined with advanced data analysis (e.g., exploring moderators and individual responses), we can work towards developing a targeted, scalable, and evidence-based intervention framework. This will provide precise, personalized guidance for clinical management.

## Conclusion

This systematic review and meta-analysis explored the impact of AE on global cognitive function and specific cognitive domains in patients with MCI. The findings unequivocally demonstrate that AE significantly enhances global cognition and memory in elderly individuals with MCI. Based on diverse intervention modalities, a frequency of 3–5 sessions per week is recommended. However, the optimal duration, intensity, and total duration of each intervention should be individualised and progressively adapted to each participant’s capacity. This study provides robust evidence supporting a dose-response association between AE and cognitive improvement in MCI, thereby offering practical implications for optimising future intervention strategies.

## Data Availability

The original contributions presented in the study are included in the article/**Supplementary Material**. Further inquiries can be directed to the corresponding authors.
