# Supplementary material for: Effects of aerobic exercise on cognitive function in older adults with mild cognitive impairment: a systematic review and meta-analysis
Source: Front Psychiatry. 2026 Jan 9;16:1741998. doi: 10.3389/fpsyt.2025.1741998 (PMC12827630; doi:10.3389/fpsyt.2025.1741998)
Supplement: Supplementary file 1 [file Image1.pdf]

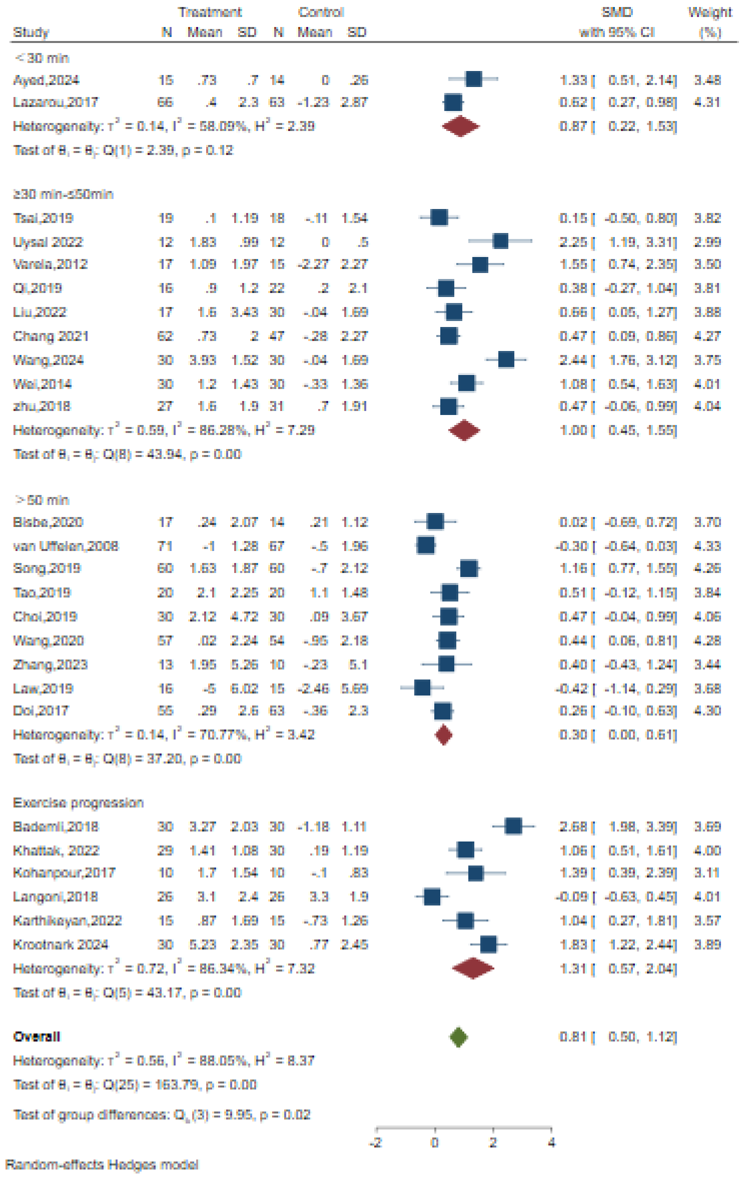

Figure 3A Forest plot for AE duration on global cognition. Exercise progression is change in exercise duration throughout intervention

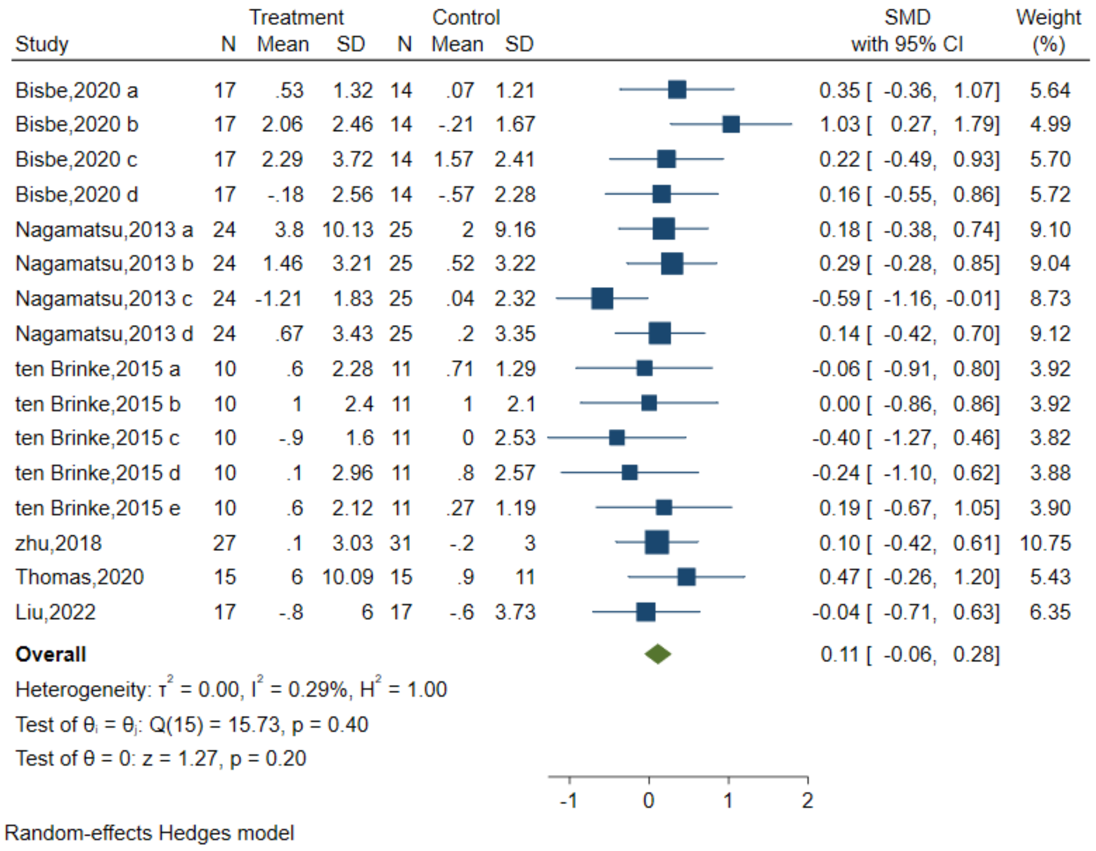

Figure 5A Forest plot for aerobic exercise on verbal memory

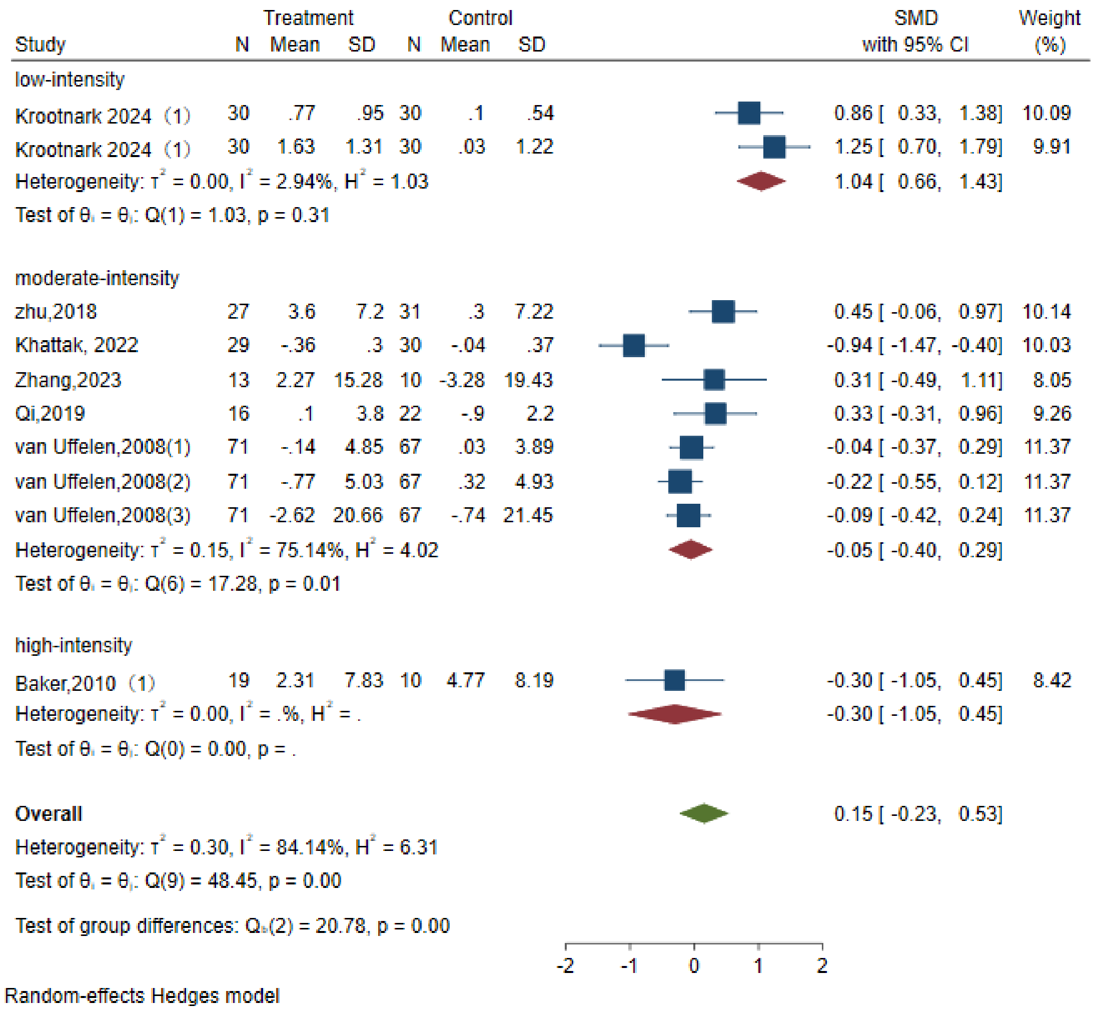

Figure 6A Forest plot for aerobic exercise intensity on attention

a) Global cognition

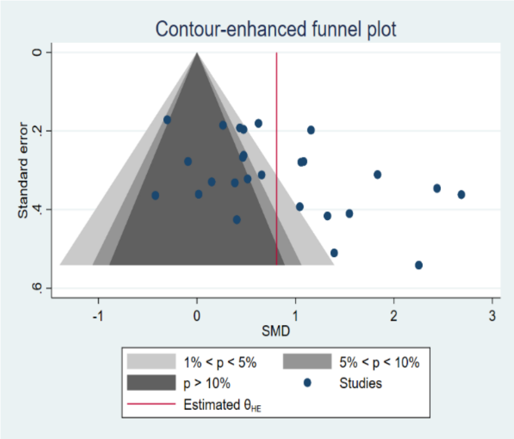

b) Executive function

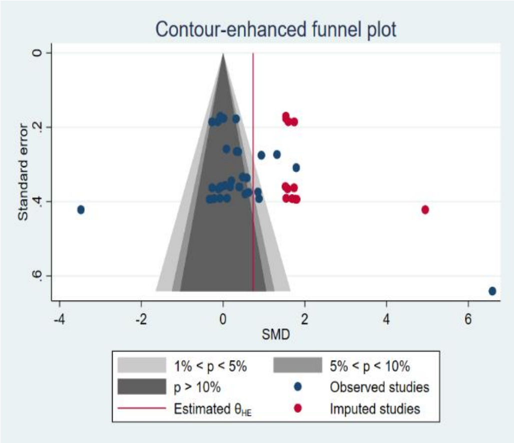

c) Attention

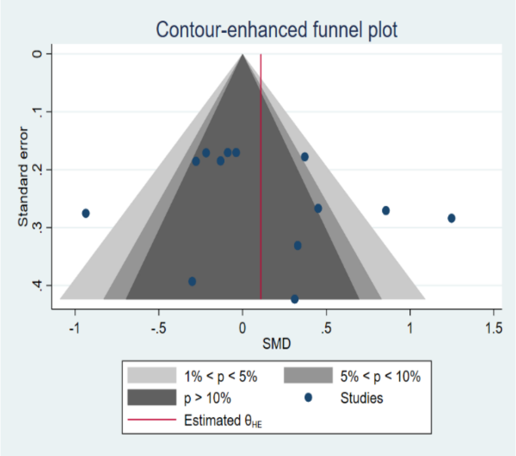

d) Memory

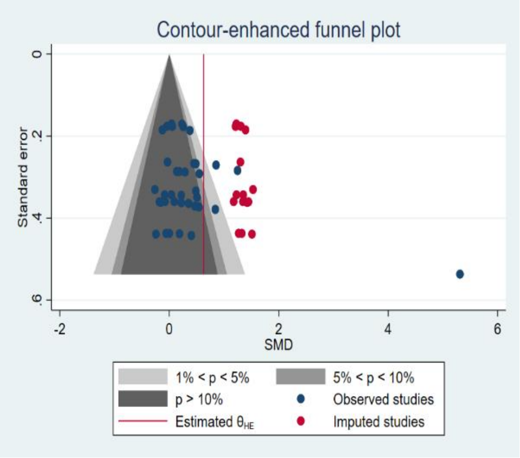

Figure S1. Funnel plots

a) ≤12 weeks

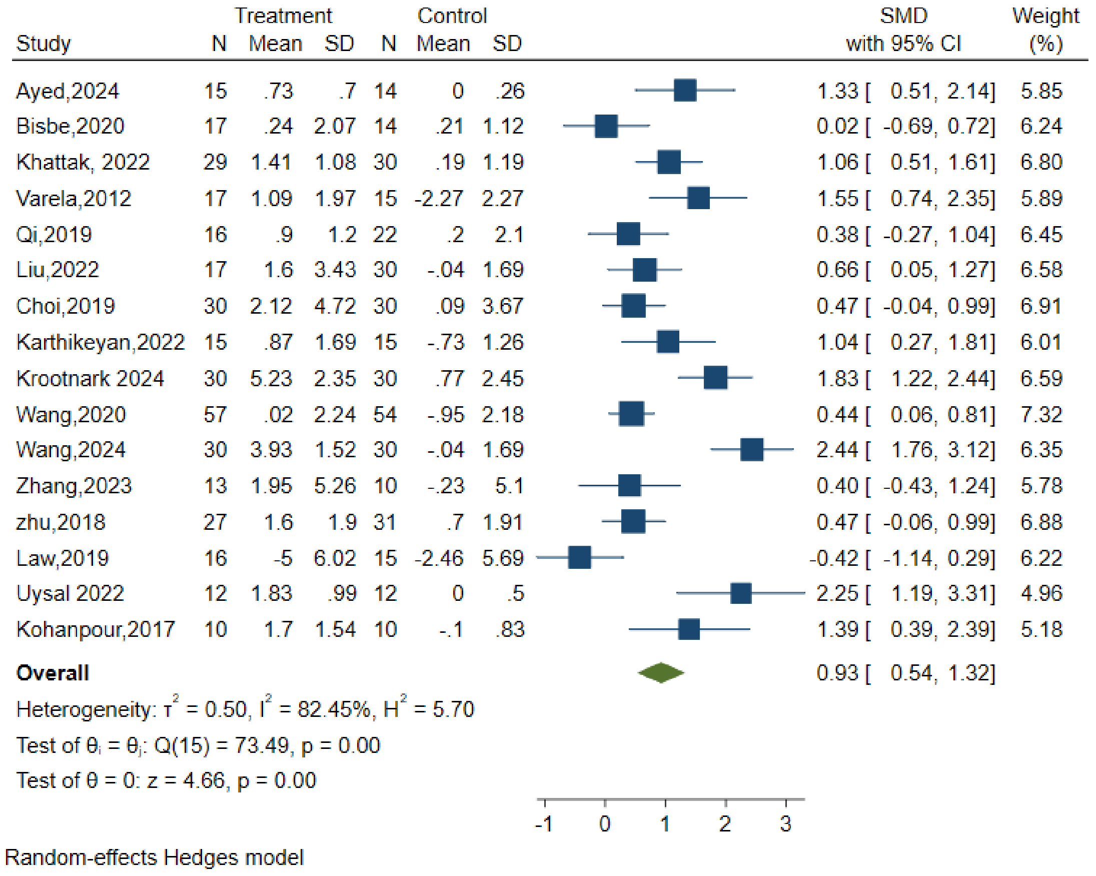

b) ＞12 weeks

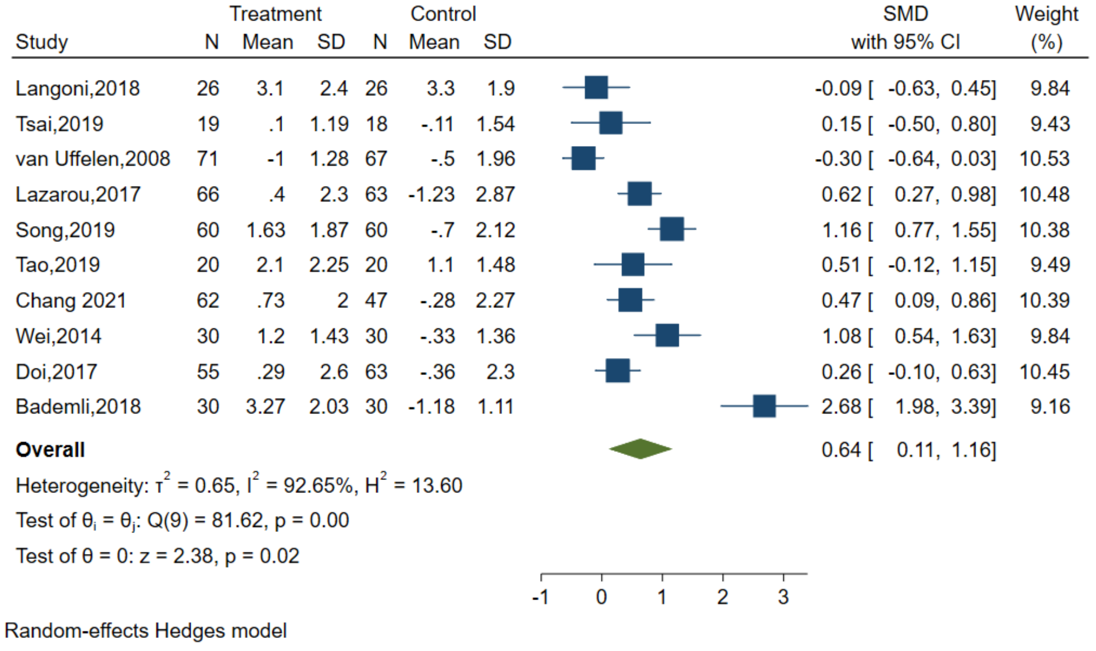

Figure S2A Subgroup analysis of the over length of the intervention in global cognition

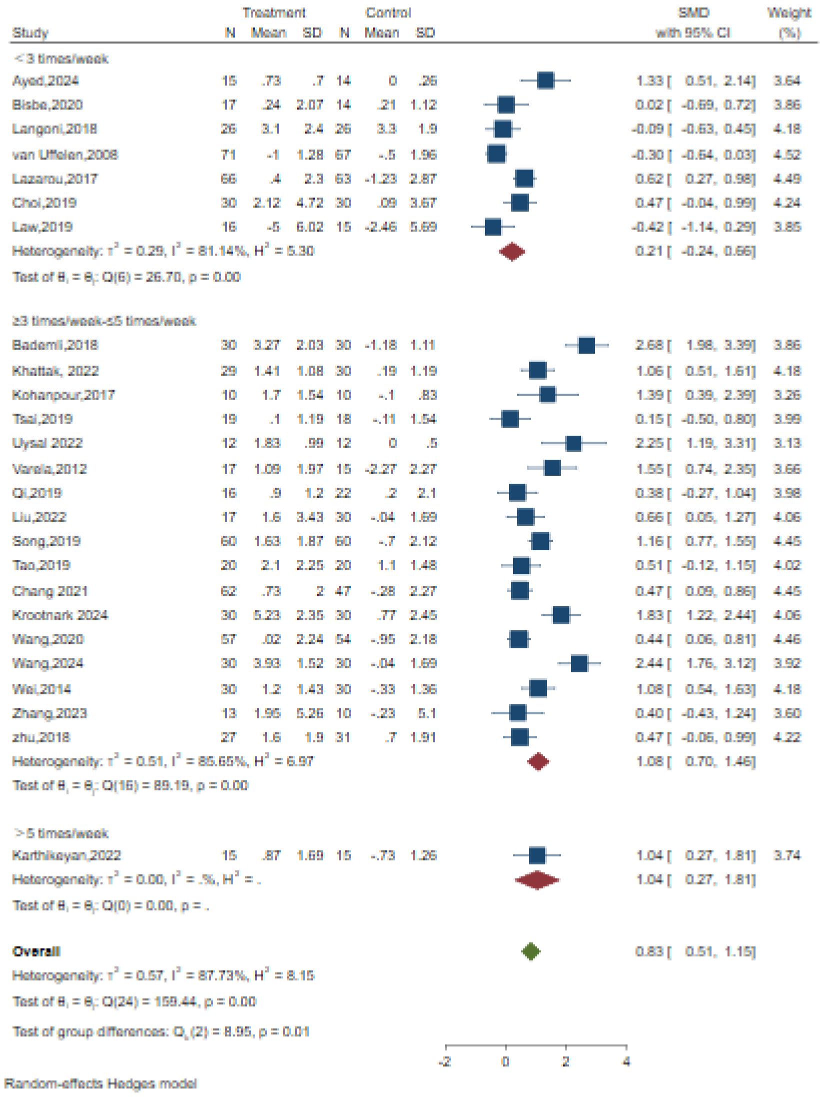

Figure S2B Subgroup analysis of the intervention frequency in global cognition

a) “low-intensity” intervention on global intervention

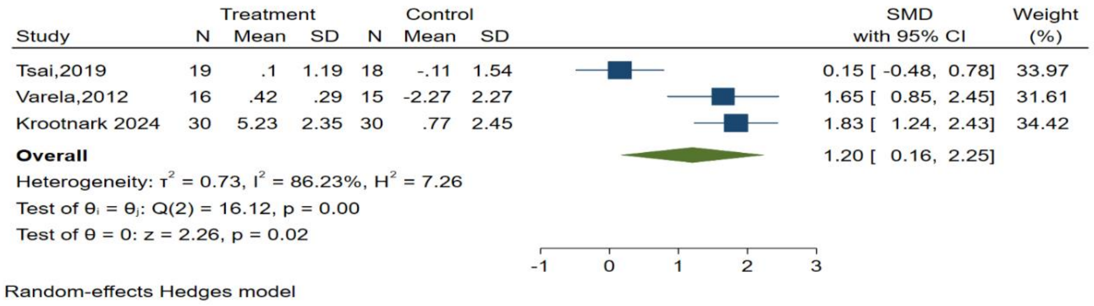

b) “moderate-intensity” intervention on global intervention

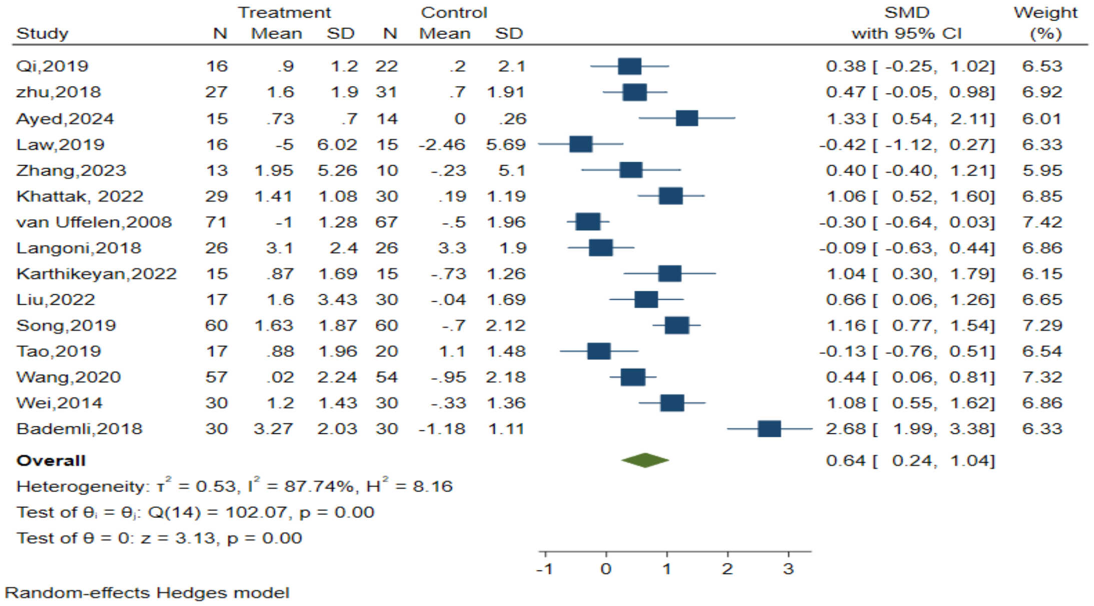

c) “high-intensity” intervention on global intervention

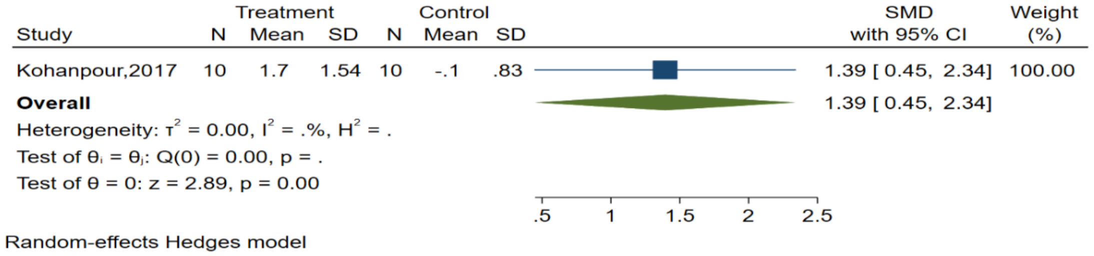

d) “Varies with the intensity of the intervention process” intervention on global intervention

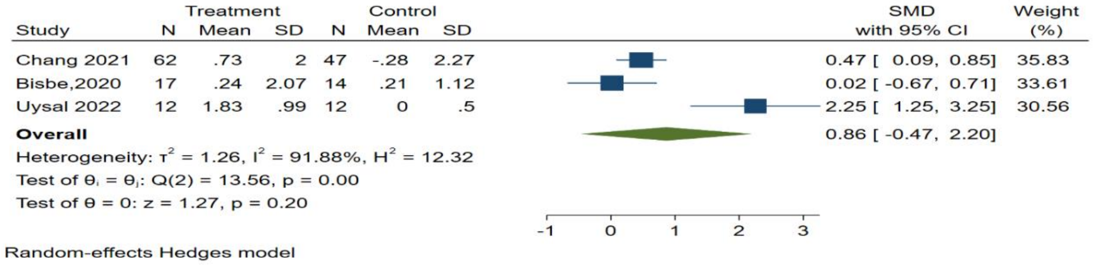

Figure S2C Subgroup analysis of the intervention intensity in global cognition

a) “dance” intervention on global intervention

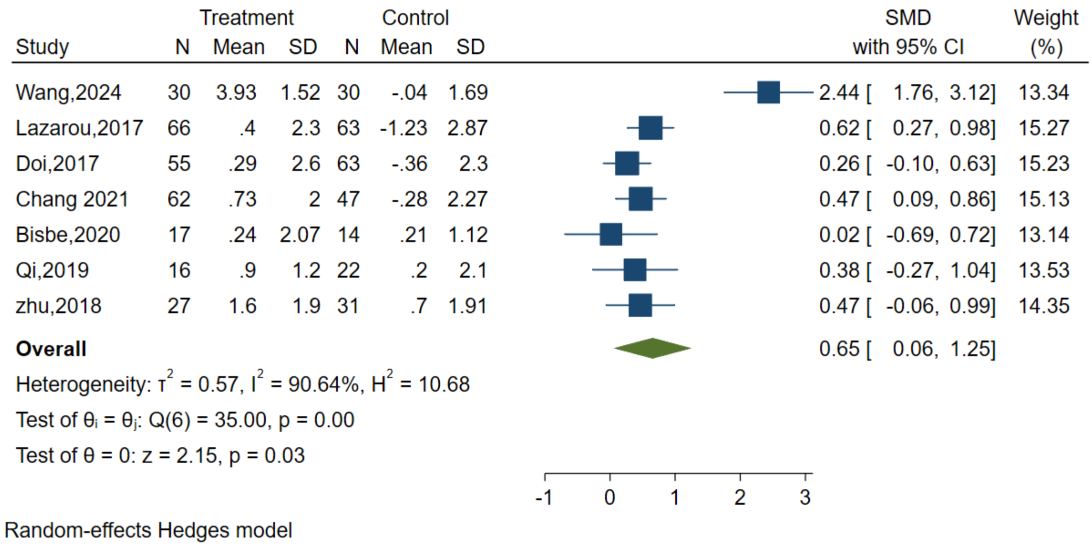

b)“walk” intervention on global intervention

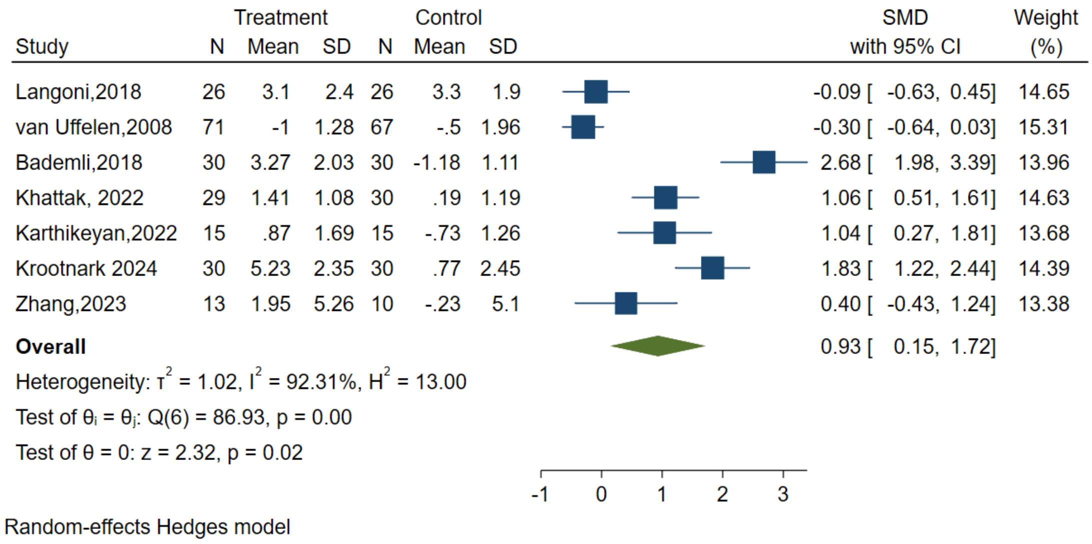

e) “bicycle” intervention on global intervention

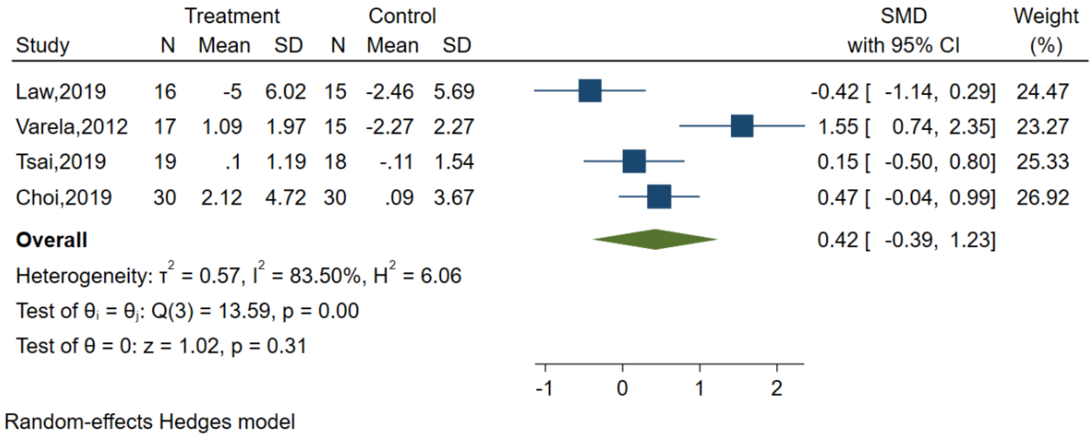

Figure S2D Subgroup analysis of different intervention type in global cognition

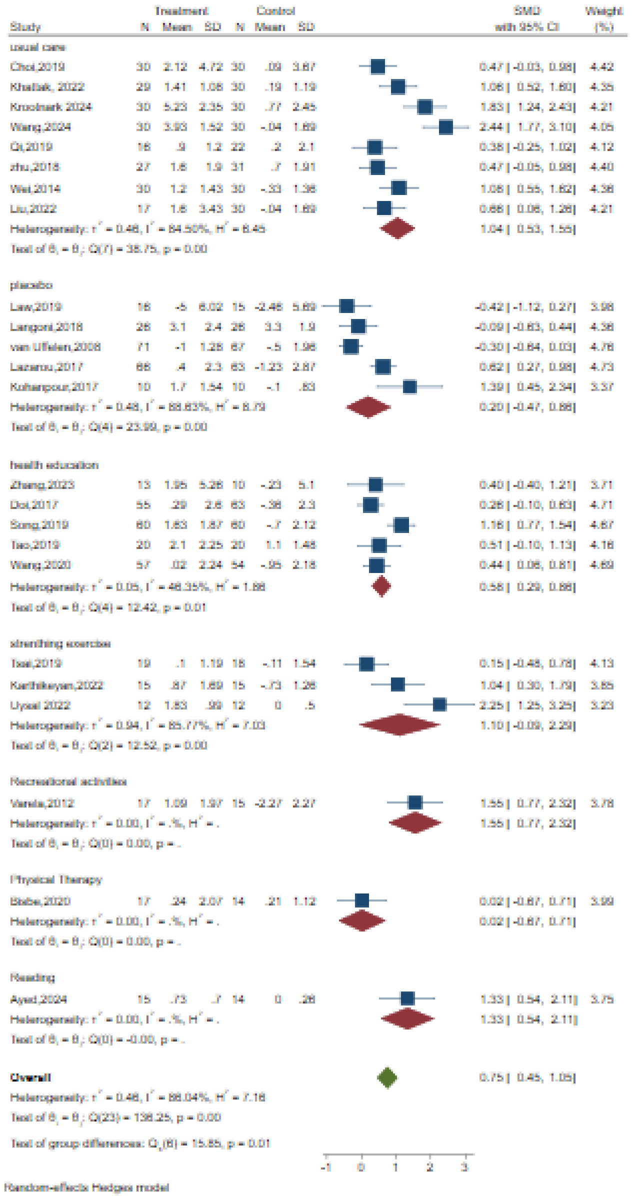

Figure S2E Subgroup analysis of different control type in global cognition

a) ≤12 weeks

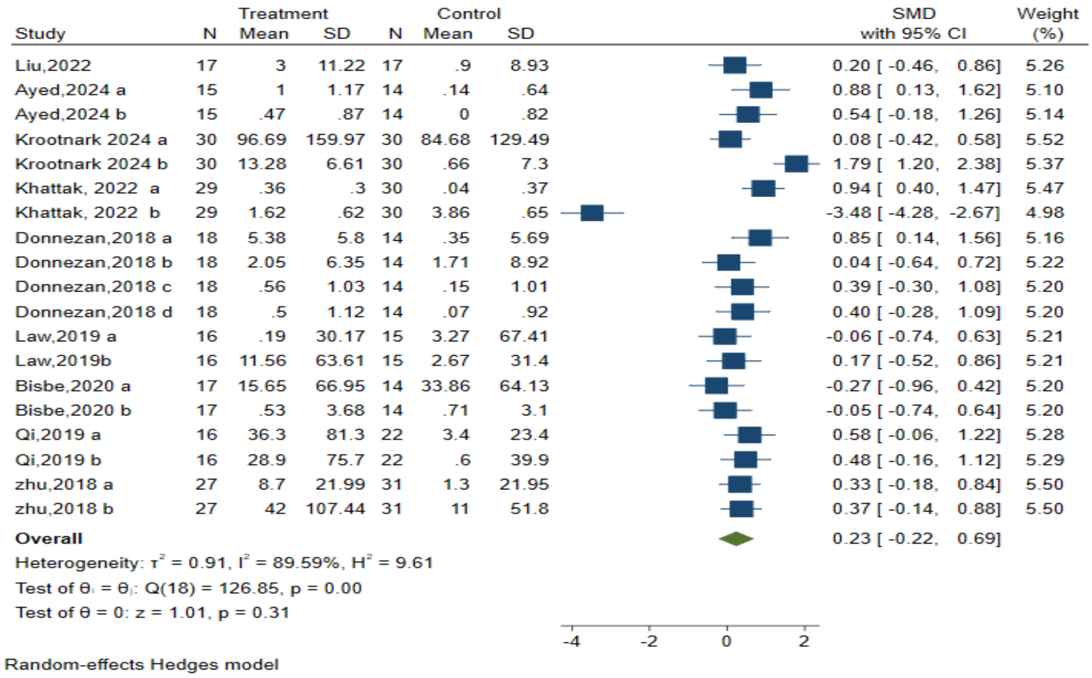

Study	Treatment N	Treatment Mean	Treatment SD	Control N	Control Mean	Control SD	SMD with 95% CI	Weight (%)
Liu,2022	17	3	11.22	17	.9	8.93	0.20 [-0.46, 0.86]	5.26
Ayed,2024 a	15	1	1.17	14	.14	.64	0.88 [0.13, 1.62]	5.10
Ayed,2024 b	15	.47	.87	14	0	.82	0.54 [-0.18, 1.26]	5.14
Krootnark 2024 a	30	96.69	159.97	30	84.68	129.49	0.08 [-0.42, 0.58]	5.52
Krootnark 2024 b	30	13.28	6.61	30	.66	7.3	1.79 [1.20, 2.38]	5.37
Khattak, 2022 a	29	.36	.3	30	.04	.37	0.94 [0.40, 1.47]	5.47
Khattak, 2022 b	29	1.62	.62	30	3.86	.65	-3.48 [-4.28, -2.67]	4.98
Donnezan,2018 a	18	5.38	5.8	14	.35	5.69	0.85 [0.14, 1.56]	5.16
Donnezan,2018 b	18	2.05	6.35	14	1.71	8.92	0.04 [-0.64, 0.72]	5.22
Donnezan,2018 c	18	.56	1.03	14	.15	1.01	0.39 [-0.30, 1.08]	5.20
Donnezan,2018 d	18	.5	1.12	14	.07	.92	0.40 [-0.28, 1.09]	5.20
Law,2019 a	16	.19	30.17	15	3.27	67.41	-0.06 [-0.74, 0.63]	5.21
Law,2019b	16	11.56	63.61	15	2.67	31.4	0.17 [-0.52, 0.86]	5.21
Bisbe,2020 a	17	15.65	66.95	14	33.86	64.13	-0.27 [-0.96, 0.42]	5.20
Bisbe,2020 b	17	.53	3.68	14	.71	3.1	-0.05 [-0.74, 0.64]	5.20
Qi,2019 a	16	36.3	81.3	22	3.4	23.4	0.58 [-0.06, 1.22]	5.28
Qi,2019 b	16	28.9	75.7	22	.6	39.9	0.48 [-0.16, 1.12]	5.29
zhu,2018 a	27	8.7	21.99	31	1.3	21.95	0.33 [-0.18, 0.84]	5.50
zhu,2018 b	27	42	107.44	31	11	51.8	0.37 [-0.14, 0.88]	5.50
**Overall**							0.23 [-0.22, 0.69]	

Heterogeneity: τ^2 = 0.91, I^2 = 89.59%, H^2 = 9.61

Test of $\theta_i = \theta_j$: Q(18) = 126.85, p = 0.00

Test of θ = 0: z = 1.01, p = 0.31

Random-effects Hedges model

b) ＞12 weeks

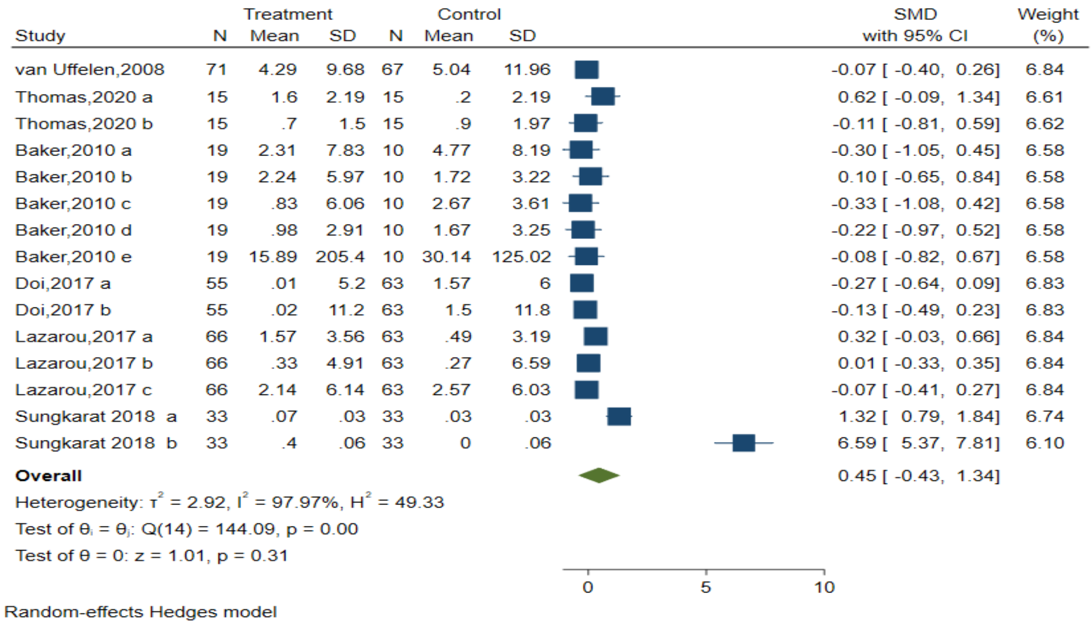

Study	Treatment N	Treatment Mean	Treatment SD	Control N	Control Mean	Control SD	SMD with 95% CI	Weight (%)
van Uffelen,2008	71	4.29	9.68	67	5.04	11.96	-0.07 [-0.40, 0.26]	6.84
Thomas,2020 a	15	1.6	2.19	15	.2	2.19	0.62 [-0.09, 1.34]	6.61
Thomas,2020 b	15	.7	1.5	15	.9	1.97	-0.11 [-0.81, 0.59]	6.62
Baker,2010 a	19	2.31	7.83	10	4.77	8.19	-0.30 [-1.05, 0.45]	6.58
Baker,2010 b	19	2.24	5.97	10	1.72	3.22	0.10 [-0.65, 0.84]	6.58
Baker,2010 c	19	.83	6.06	10	2.67	3.61	-0.33 [-1.08, 0.42]	6.58
Baker,2010 d	19	.98	2.91	10	1.67	3.25	-0.22 [-0.97, 0.52]	6.58
Baker,2010 e	19	15.89	205.4	10	30.14	125.02	-0.08 [-0.82, 0.67]	6.58
Doi,2017 a	55	.01	5.2	63	1.57	6	-0.27 [-0.64, 0.09]	6.83
Doi,2017 b	55	.02	11.2	63	1.5	11.8	-0.13 [-0.49, 0.23]	6.83
Lazarou,2017 a	66	1.57	3.56	63	.49	3.19	0.32 [-0.03, 0.66]	6.84
Lazarou,2017 b	66	.33	4.91	63	.27	6.59	0.01 [-0.33, 0.35]	6.84
Lazarou,2017 c	66	2.14	6.14	63	2.57	6.03	-0.07 [-0.41, 0.27]	6.84
Sungkarat 2018 a	33	.07	.03	33	.03	.03	1.32 [0.79, 1.84]	6.74
Sungkarat 2018 b	33	.4	.06	33	0	.06	6.59 [5.37, 7.81]	6.10
**Overall**							0.45 [-0.43, 1.34]	

Heterogeneity: τ^2 = 2.92, I^2 = 97.97%, H^2 = 49.33

Test of $\theta_i = \theta_j$: Q(14) = 144.09, p = 0.00

Test of θ = 0: z = 1.01, p = 0.31

Random-effects Hedges model

Figure S3A Subgroup analysis of the over length of the intervention in executive functions

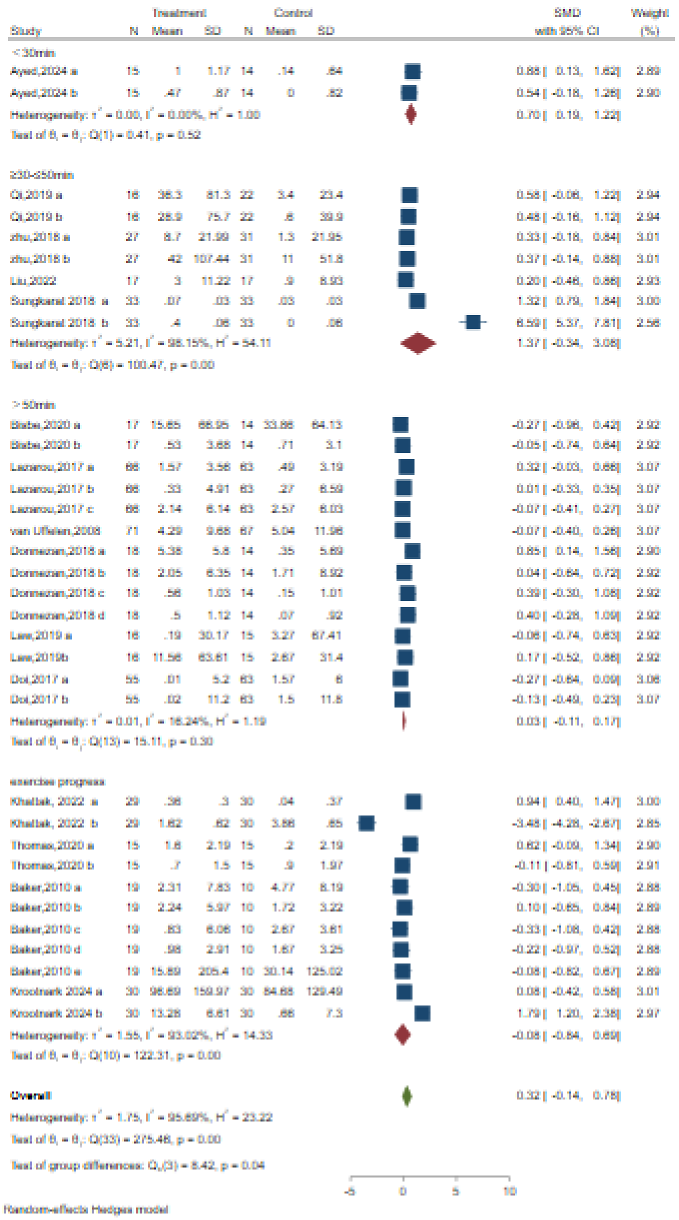

Figure S3B Subgroup analysis of the intervention duration in executive functions

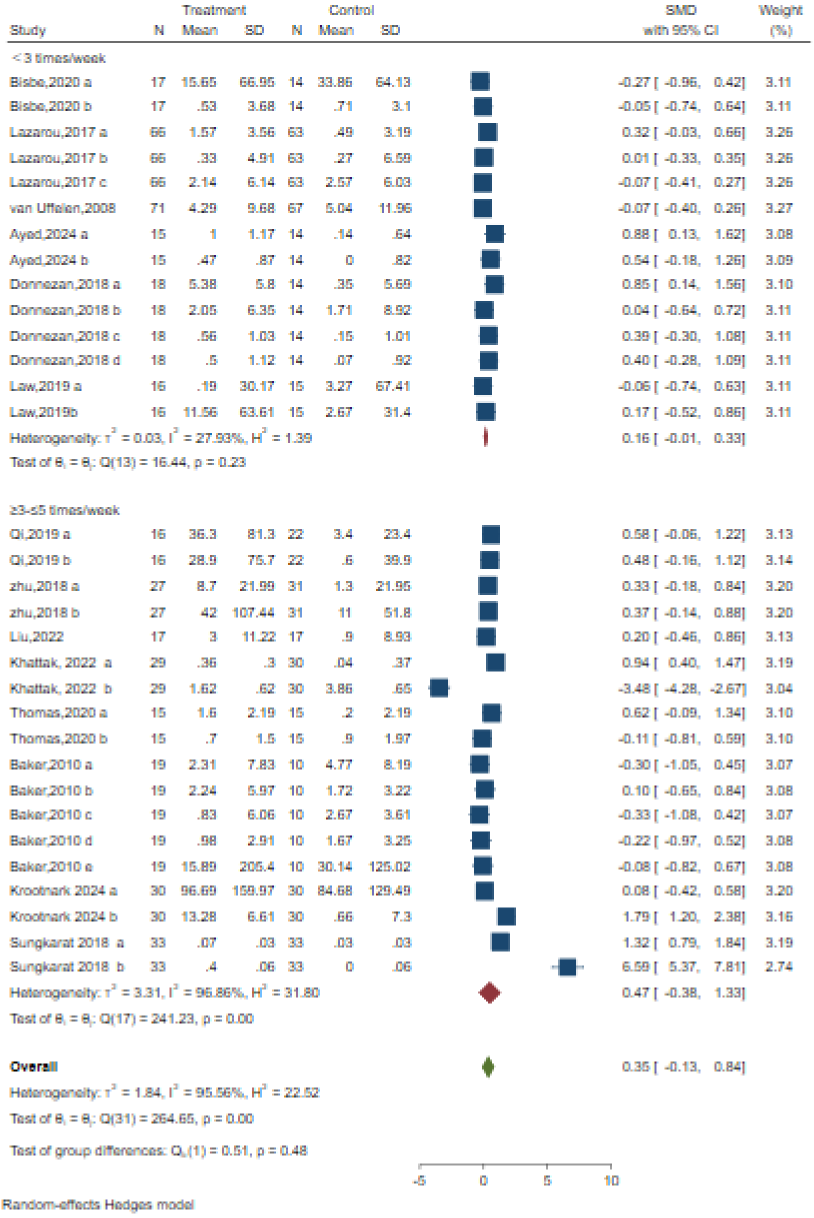

Figure S3C Subgroup analysis of the intervention frequency in executive functions

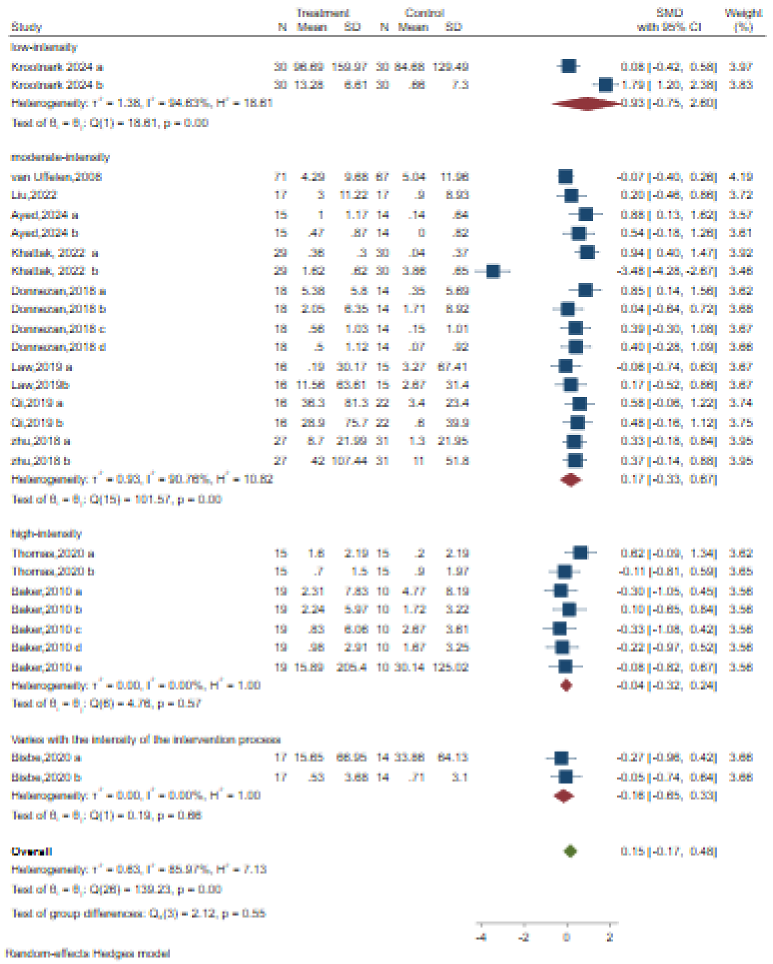

Figure S3D Subgroup analysis of the intervention intensity in executive functions

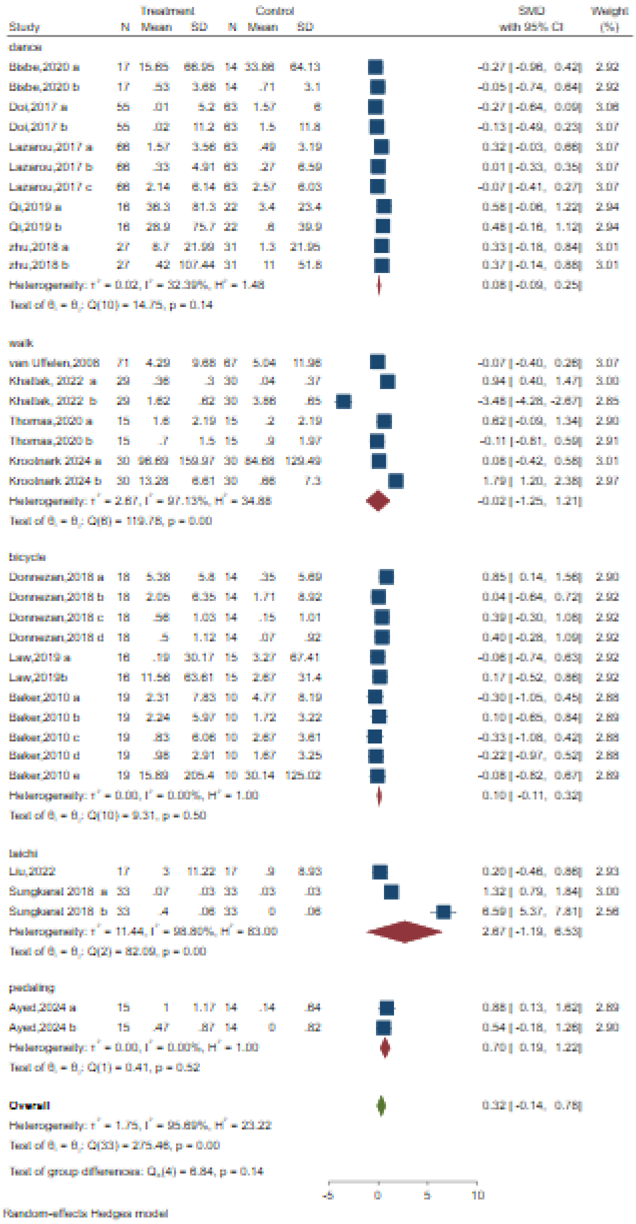

Figure S3E Subgroup analysis of the different intervention type in executive functions

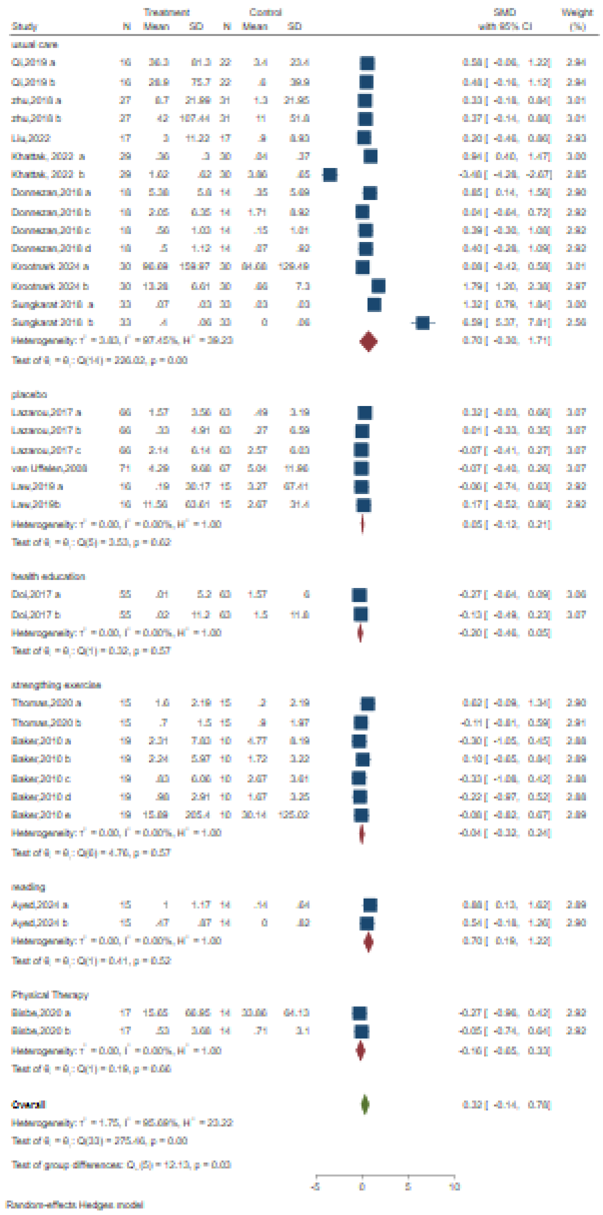

Figure S3F Subgroup analysis of the different control type in executive functions

a) ≤12 weeks

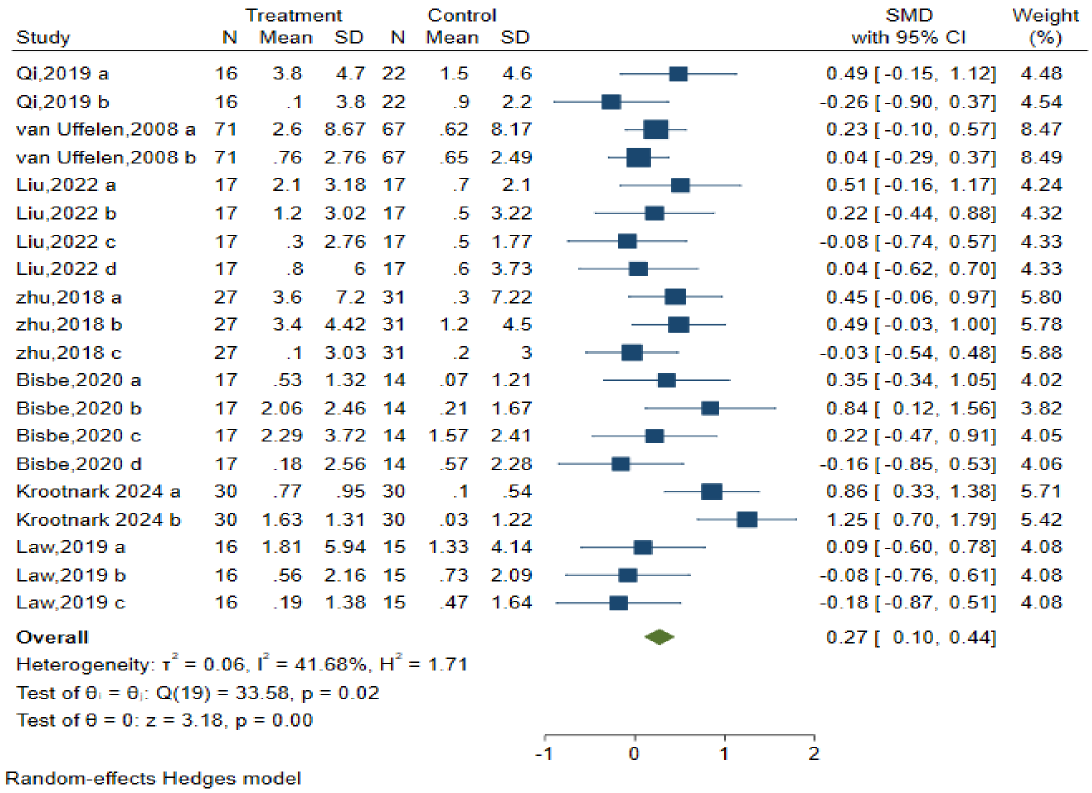

b) ＞12 weeks

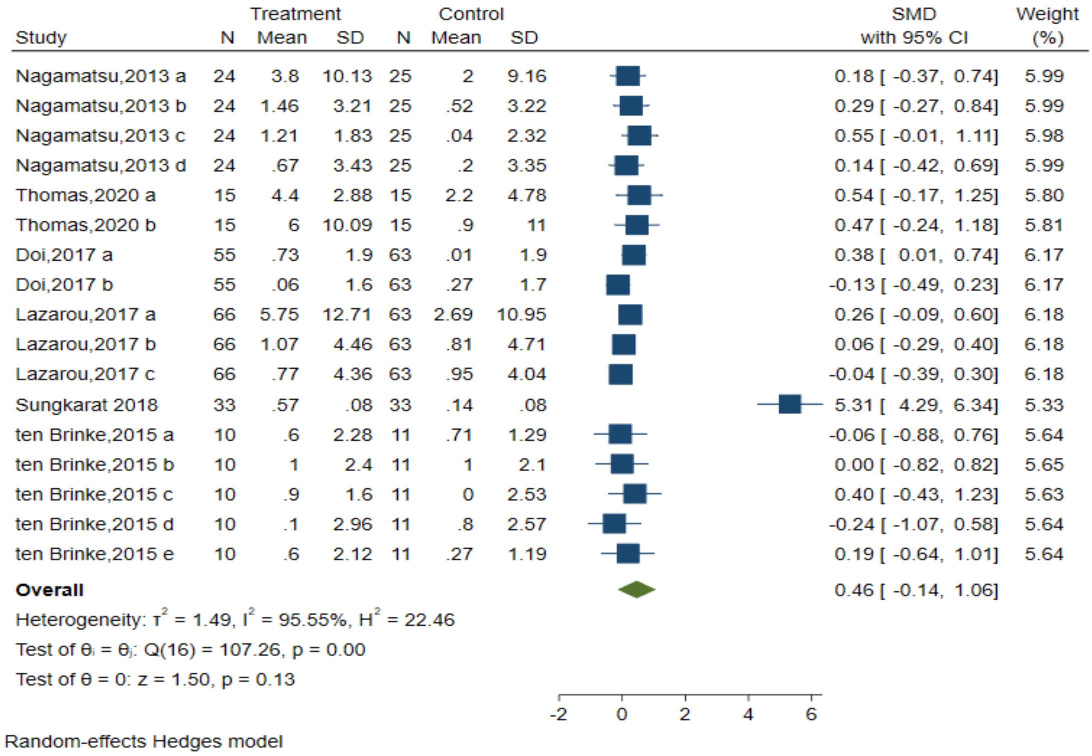

Figure S4A Subgroup analysis of the over length of the intervention in memory

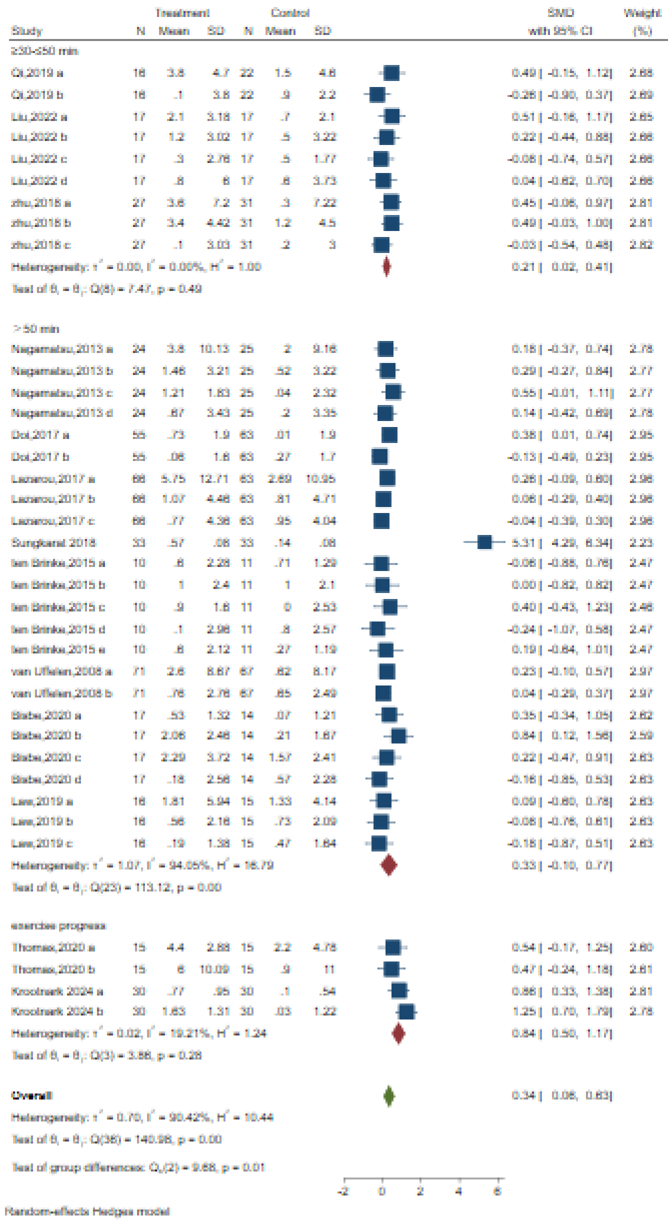

Figure S4B Subgroup analysis of the intervention duration in memory

a) < 3 times/week

Study	Treatment N	Treatment Mean	Treatment SD	Control N	Control Mean	Control SD	SMD with 95% CI	Weight (%)
Nagamatsu,2013 a	24	3.8	10.13	25	2	9.16	0.18 [-0.37, 0.74]	4.18
Nagamatsu,2013 b	24	1.46	3.21	25	.52	3.22	0.29 [-0.27, 0.84]	4.16
Nagamatsu,2013 c	24	1.21	1.83	25	.04	2.32	0.55 [-0.01, 1.11]	4.04
Nagamatsu,2013 d	24	.67	3.43	25	.2	3.35	0.14 [-0.42, 0.69]	4.19
Lazarou,2017 a	66	5.75	12.71	63	2.69	10.95	0.26 [-0.09, 0.60]	10.74
Lazarou,2017 b	66	1.07	4.46	63	.81	4.71	0.06 [-0.29, 0.40]	10.83
Lazarou,2017 c	66	.77	4.36	63	.95	4.04	-0.04 [-0.39, 0.30]	10.83
ten Brinke,2015 a	10	.6	2.28	11	.71	1.29	-0.06 [-0.88, 0.76]	1.89
ten Brinke,2015 b	10	1	2.4	11	1	2.1	0.00 [-0.82, 0.82]	1.89
ten Brinke,2015 c	10	.9	1.6	11	0	2.53	0.40 [-0.43, 1.23]	1.85
ten Brinke,2015 d	10	.1	2.96	11	.8	2.57	-0.24 [-1.07, 0.58]	1.87
ten Brinke,2015 e	10	.6	2.12	11	.27	1.19	0.19 [-0.64, 1.01]	1.88
van Uffelen,2008 a	71	2.6	8.67	67	.62	8.17	0.23 [-0.10, 0.57]	11.50
van Uffelen,2008 b	71	.76	2.76	67	.65	2.49	0.04 [-0.29, 0.37]	11.57
Bisbe,2020 a	17	.53	1.32	14	.07	1.21	0.35 [-0.34, 1.05]	2.65
Bisbe,2020 b	17	2.06	2.46	14	.21	1.67	0.84 [0.12, 1.56]	2.46
Bisbe,2020 c	17	2.29	3.72	14	1.57	2.41	0.22 [-0.47, 0.91]	2.67
Bisbe,2020 d	17	.18	2.56	14	.57	2.28	-0.16 [-0.85, 0.53]	2.68
Law,2019 a	16	1.81	5.94	15	1.33	4.14	0.09 [-0.60, 0.78]	2.71
Law,2019 b	16	.56	2.16	15	.73	2.09	-0.08 [-0.76, 0.61]	2.71
Law,2019 c	16	.19	1.38	15	.47	1.64	-0.18 [-0.87, 0.51]	2.70
**Overall**							0.14 [0.03, 0.25]	

Heterogeneity: τ^2 = 0.00, I^2 = 0.00%, H^2 = 1.00

Test of $\theta_i = \theta_j$: Q(20) = 12.29, p = 0.91

Test of θ = 0: z = 2.44, p = 0.01

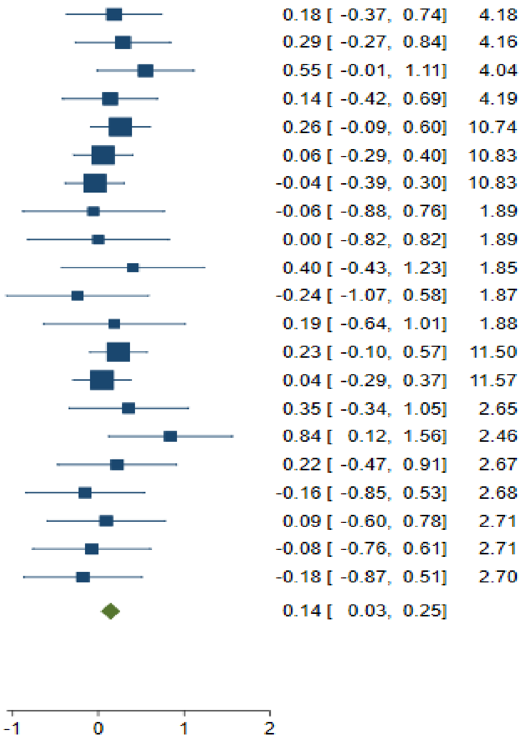

Random-effects Hedges model

b) ≤3-≤5 times/week

Study	Treatment N	Treatment Mean	Treatment SD	Control N	Control Mean	Control SD	SMD with 95% CI	Weight (%)
Thomas,2020 a	15	4.4	2.88	15	2.2	4.78	0.54 [-0.17, 1.25]	7.06
Thomas,2020 b	15	6	10.09	15	.9	11	0.47 [-0.24, 1.18]	7.07
Sungkarat 2018	33	.57	.08	33	.14	.08	5.31 [4.29, 6.34]	6.58
Qi,2019 a	16	3.8	4.7	22	1.5	4.6	0.49 [-0.15, 1.12]	7.15
Qi,2019 b	16	.1	3.8	22	.9	2.2	-0.26 [-0.90, 0.37]	7.16
Liu,2022 a	17	2.1	3.18	17	.7	2.1	0.51 [-0.16, 1.17]	7.12
Liu,2022 b	17	1.2	3.02	17	.5	3.22	0.22 [-0.44, 0.88]	7.13
Liu,2022 c	17	.3	2.76	17	.5	1.77	-0.08 [-0.74, 0.57]	7.13
Liu,2022 d	17	.8	6	17	.6	3.73	0.04 [-0.62, 0.70]	7.13
zhu,2018 a	27	3.6	7.2	31	.3	7.22	0.45 [-0.06, 0.97]	7.30
zhu,2018 b	27	3.4	4.42	31	1.2	4.5	0.49 [-0.03, 1.00]	7.30
zhu,2018 c	27	.1	3.03	31	.2	3	-0.03 [-0.54, 0.48]	7.31
Krootnark 2024 a	30	.77	.95	30	.1	.54	0.86 [0.33, 1.38]	7.29
Krootnark 2024 b	30	1.63	1.31	30	.03	1.22	1.25 [0.70, 1.79]	7.26
**Overall**							0.70 [-0.02, 1.42]	

Heterogeneity: τ^2 = 1.78, I^2 = 94.78%, H^2 = 19.16

Test of $\theta_i = \theta_j$: Q(13) = 109.14, p = 0.00

Test of θ = 0: z = 1.92, p = 0.06

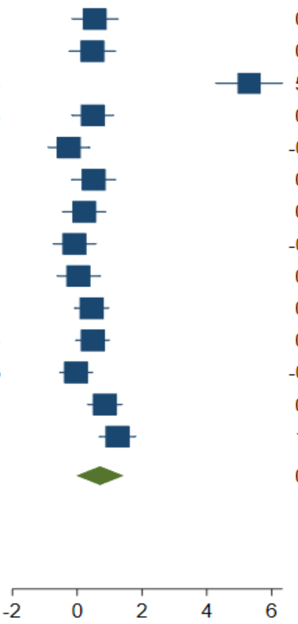

Random-effects Hedges model

Figure S4C Subgroup analysis of the intervention frequency in memory

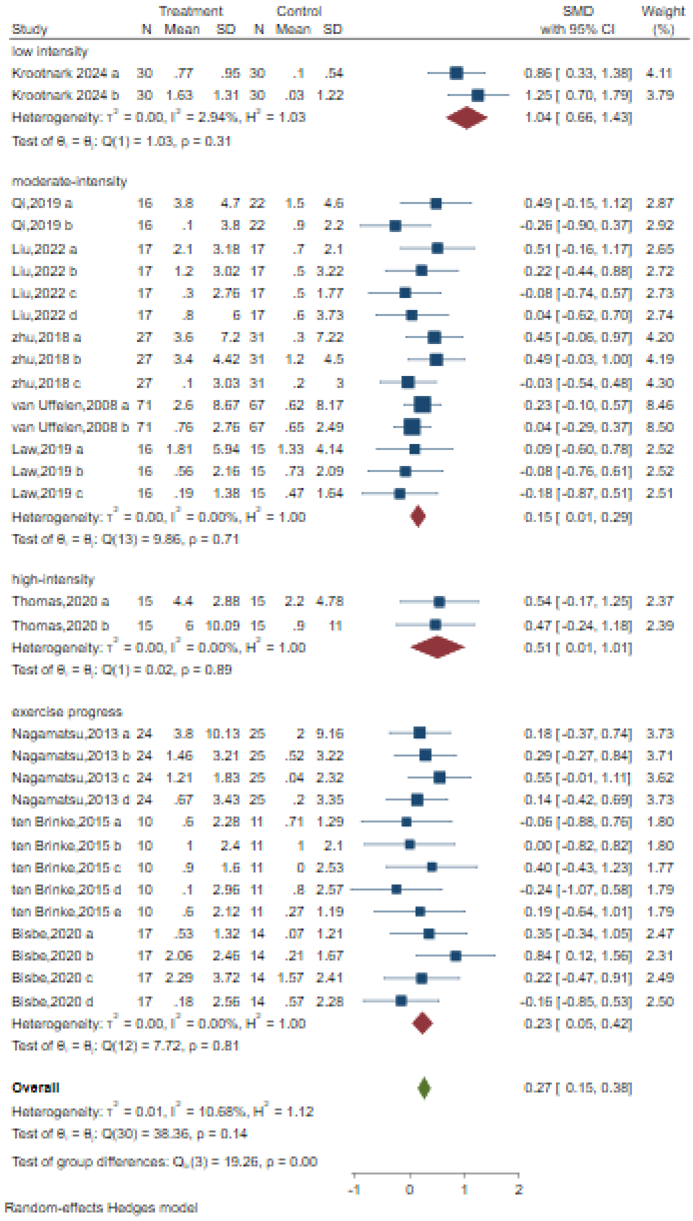

Figure S4D Subgroup analysis of the intervention intensity in memory

a) "dance" intervention on memory

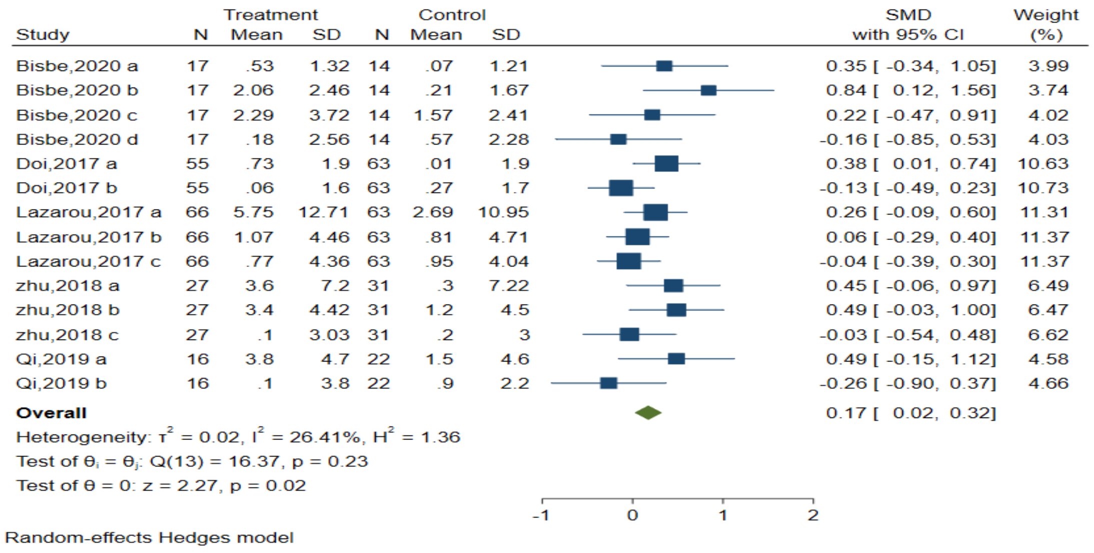

b) "walk" intervention on memory

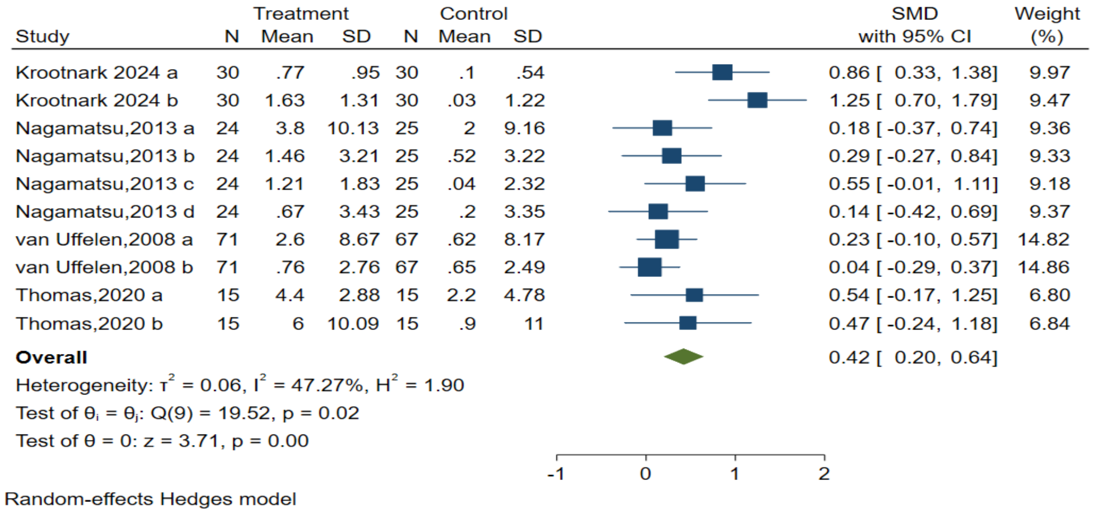

c) "bicycle" intervention on memory

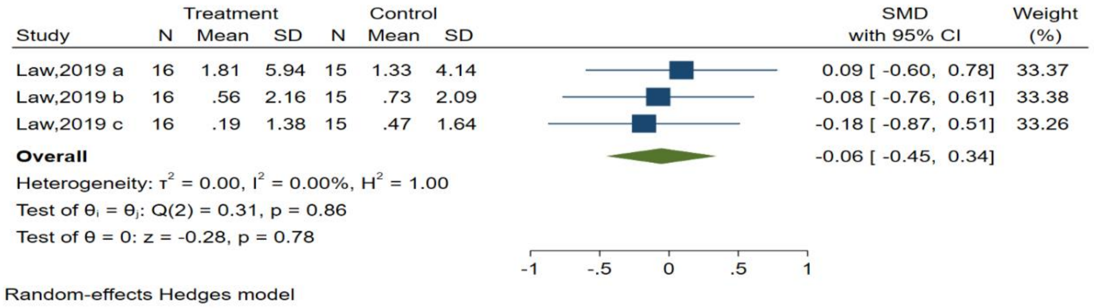

d) "taichi" intervention on memory

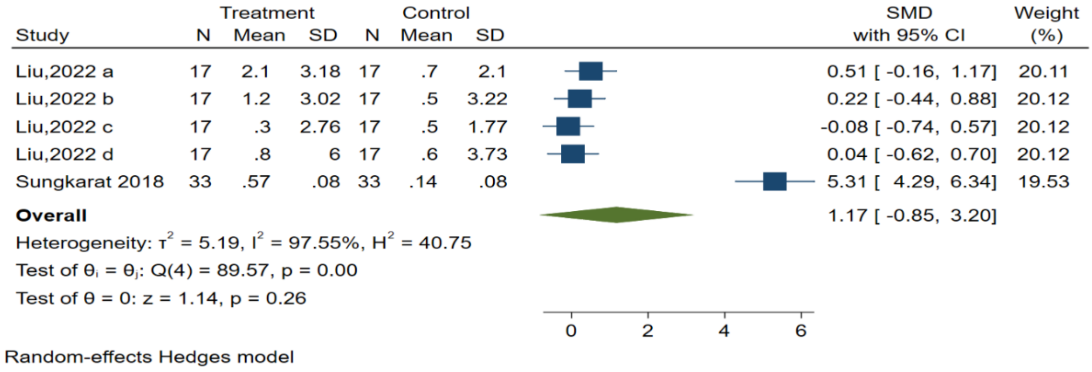

Figure S4E Subgroup analysis of the intervention type in memory

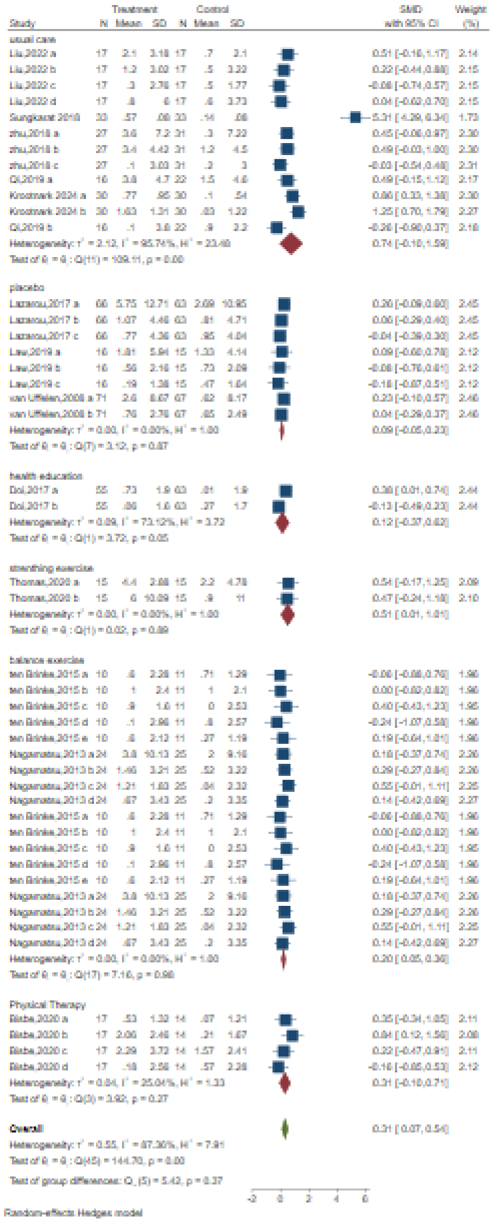

Figure S4F Subgroup analysis of the control type in memory

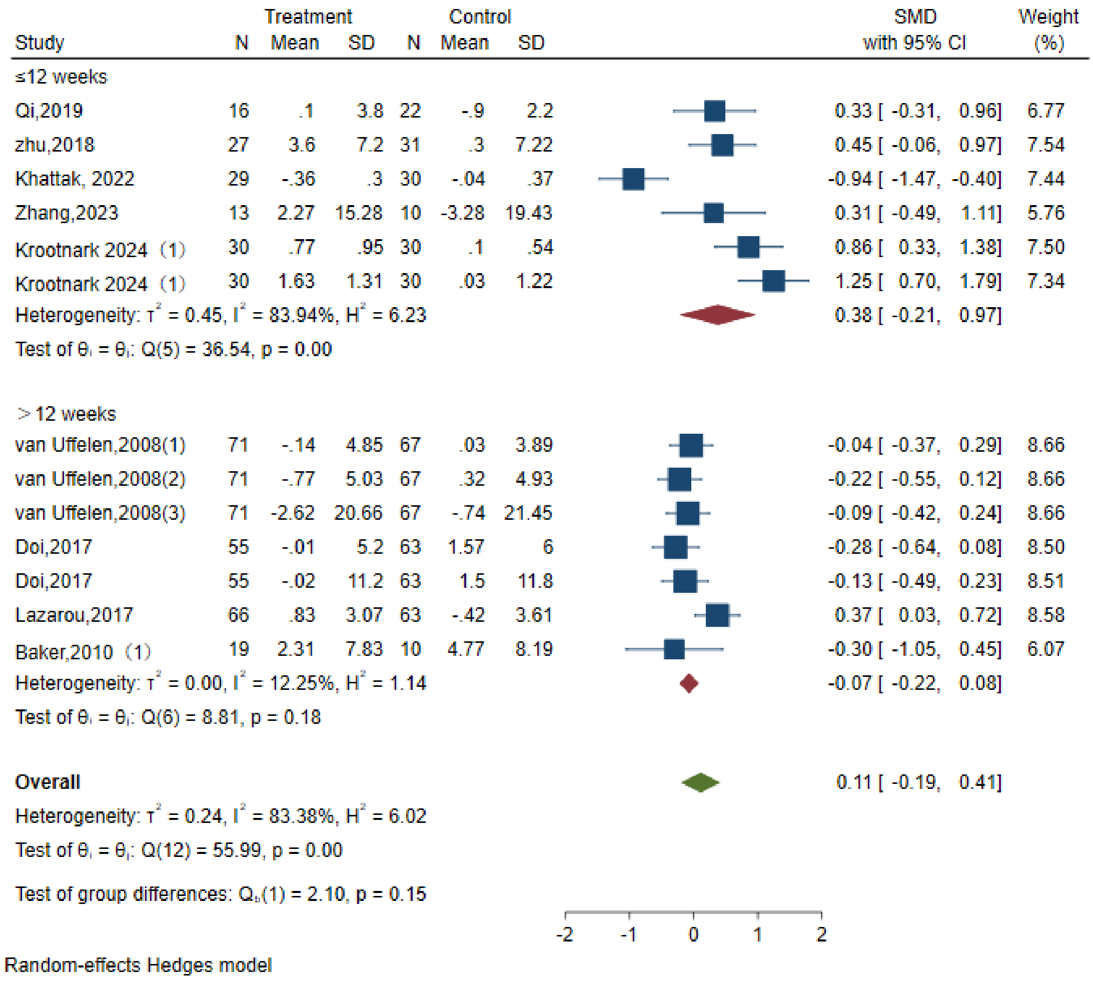

Figure S5A Subgroup analysis of the over length of the intervention in attention

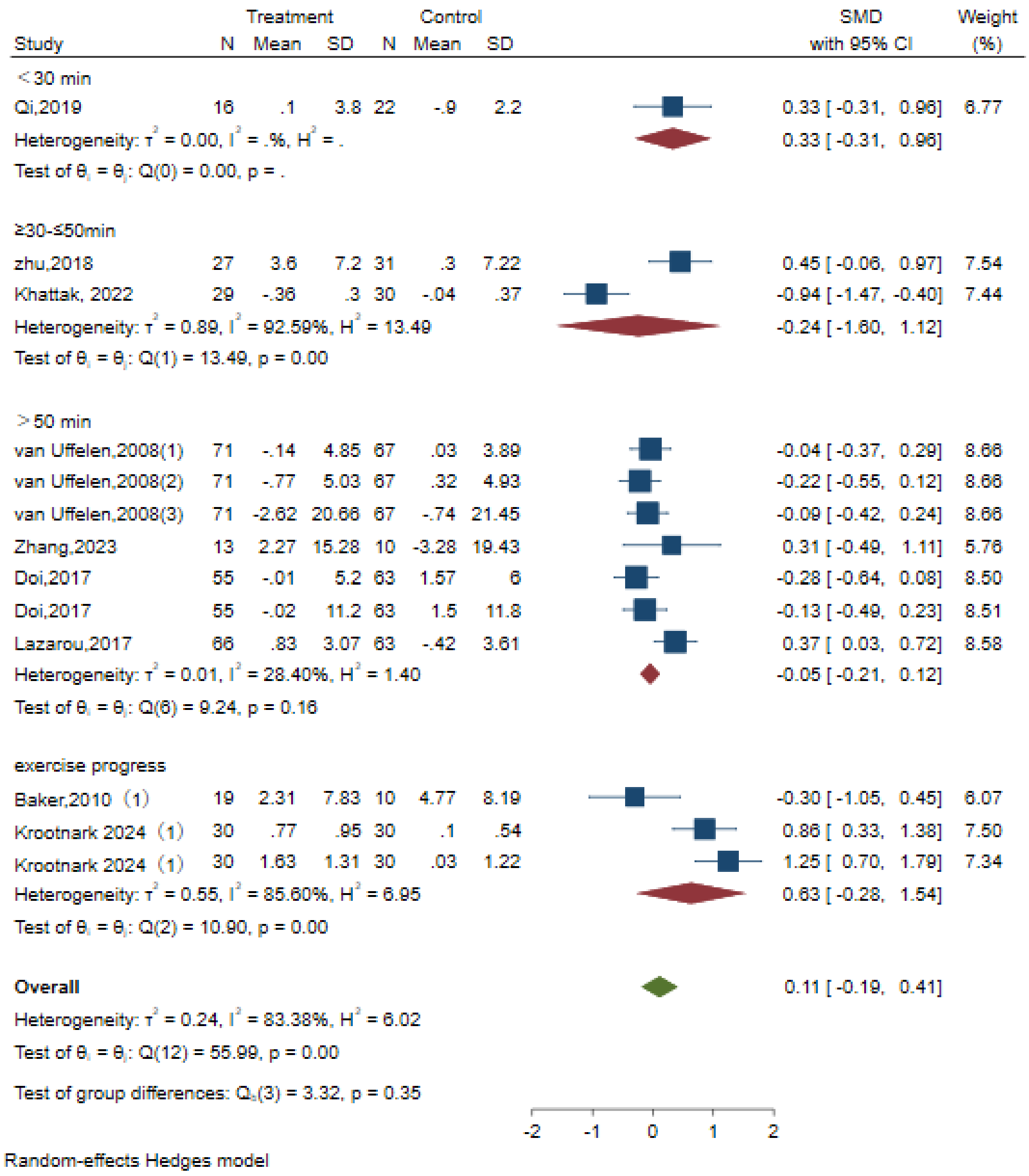

Figure S5B Subgroup analysis of the intervention duration in attention, exercise progress is time varies with exercise progress

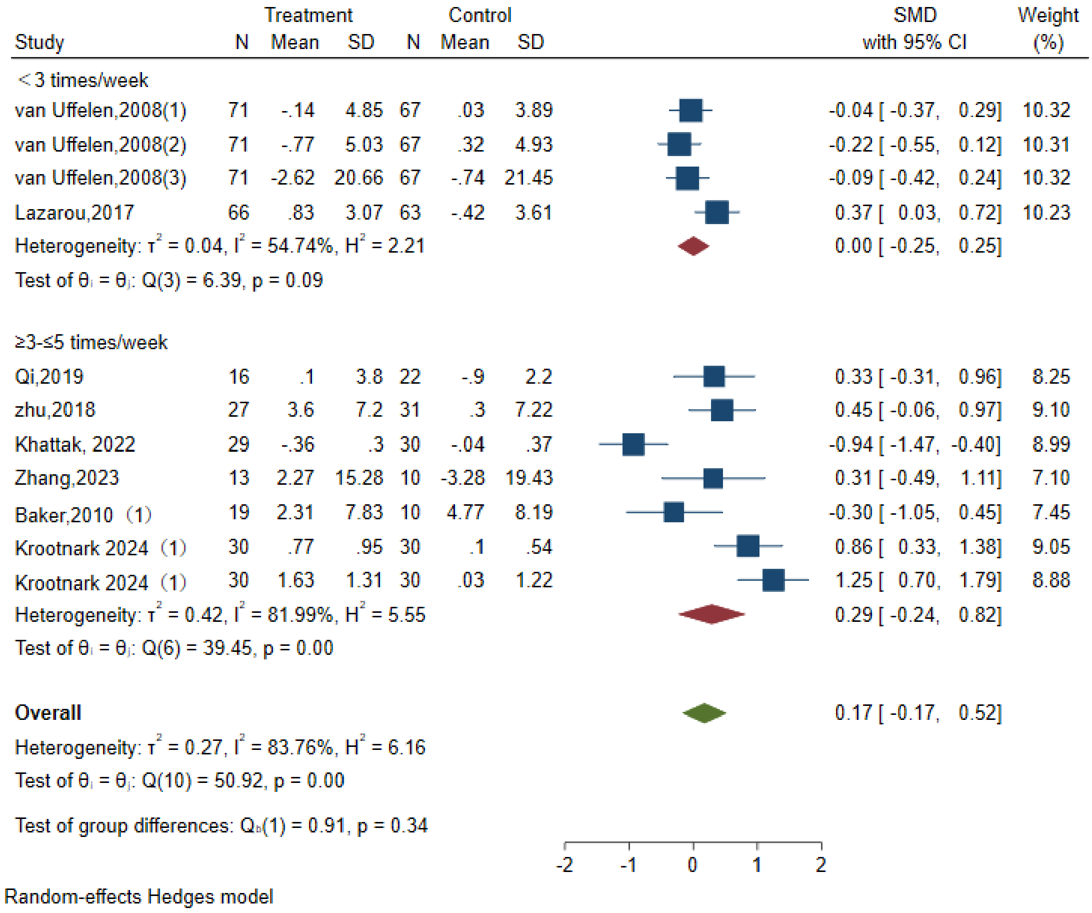

Figure S5C Subgroup analysis of the intervention frequency in attention

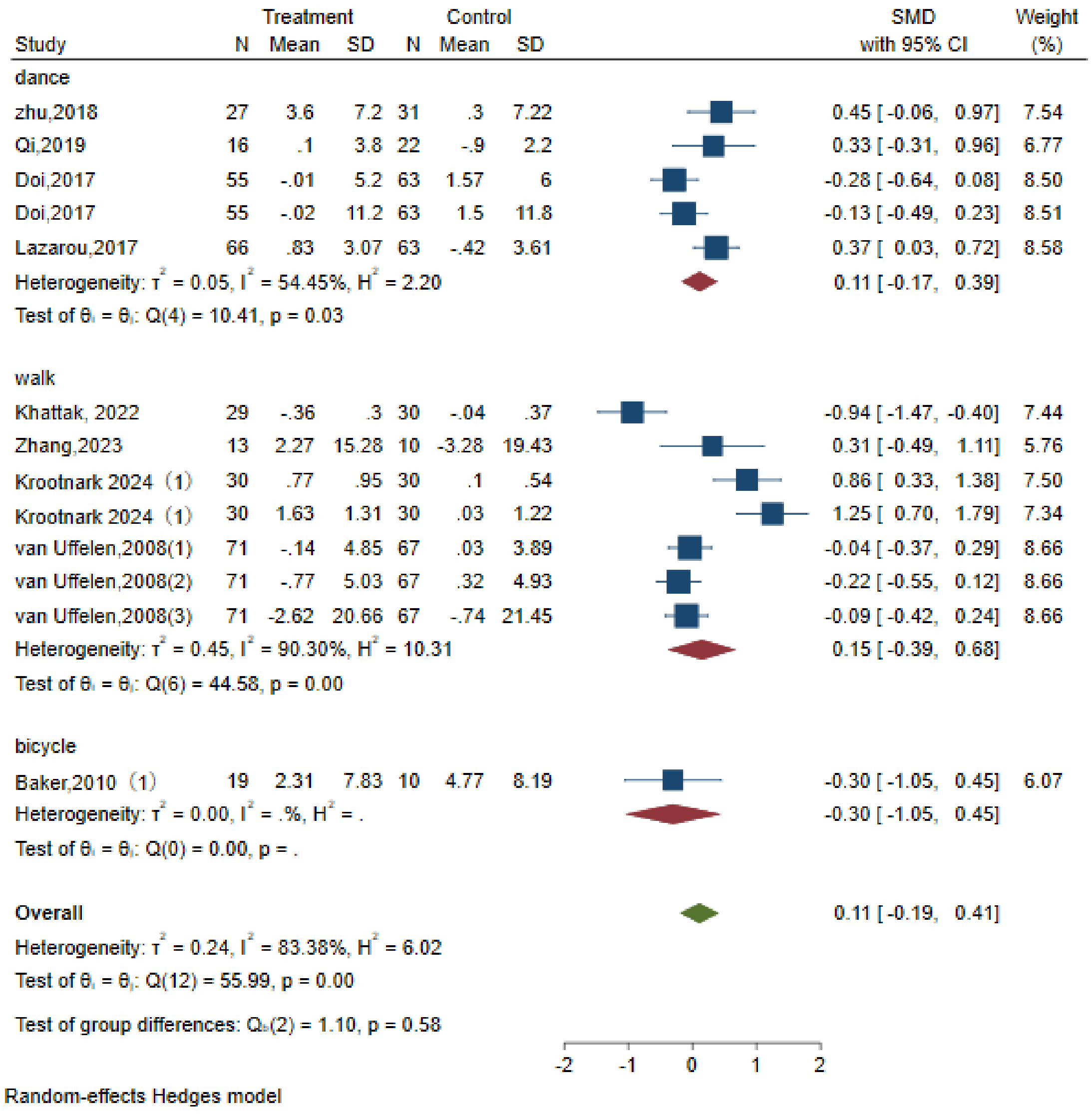

Figure S5D Subgroup analysis of the intervention type in attention

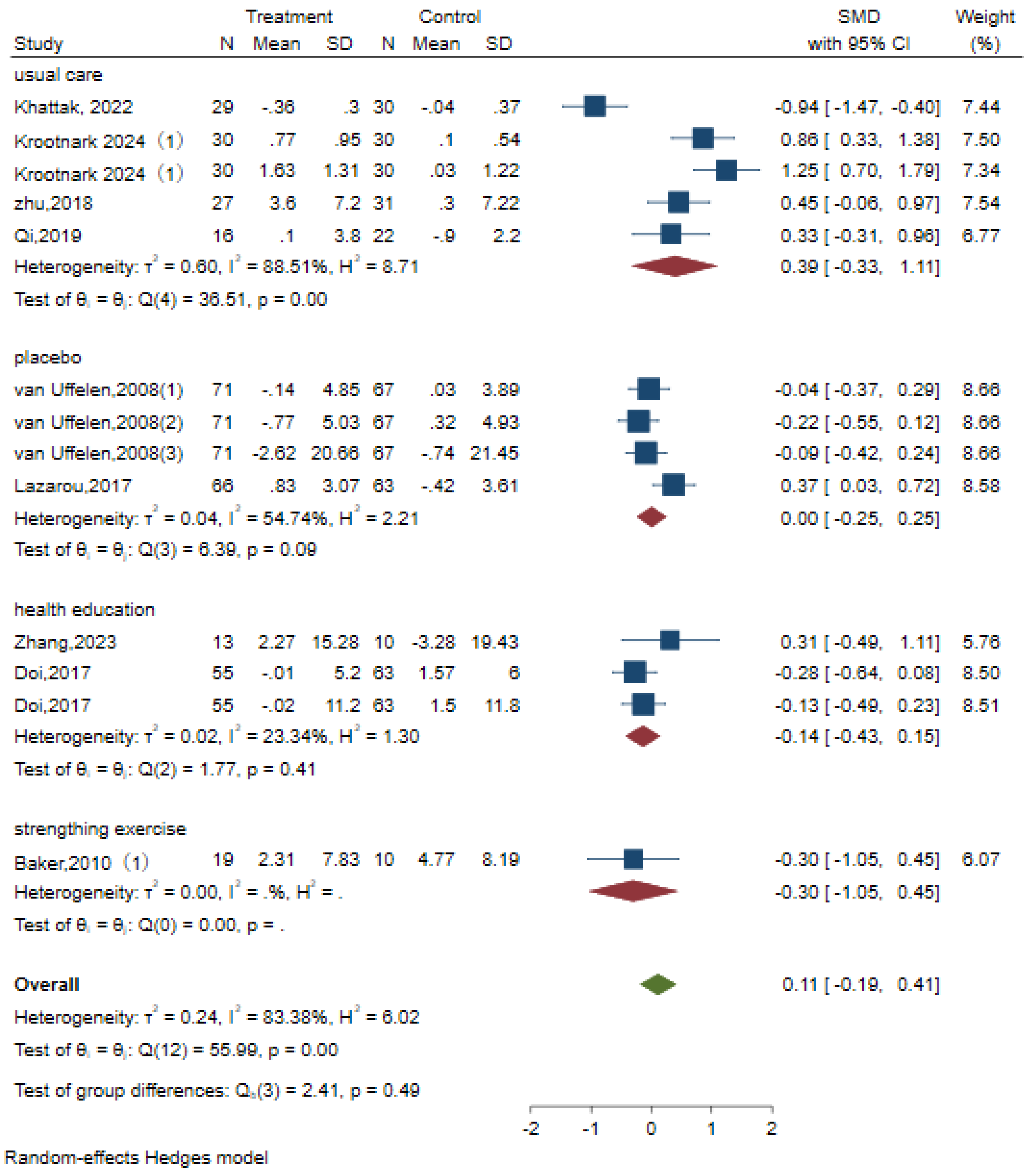

Figure S5E Subgroup analysis of the control type in attention

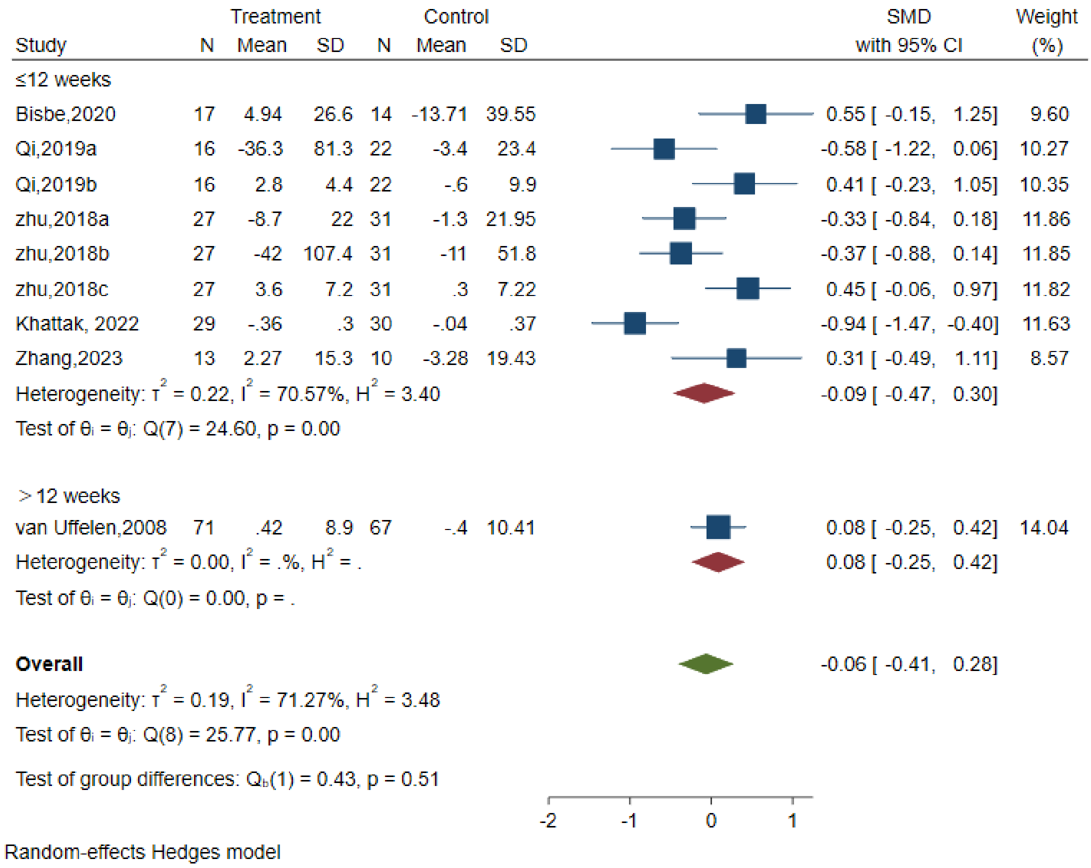

Figure S6A Subgroup analysis of the over length of the intervention in processing speed

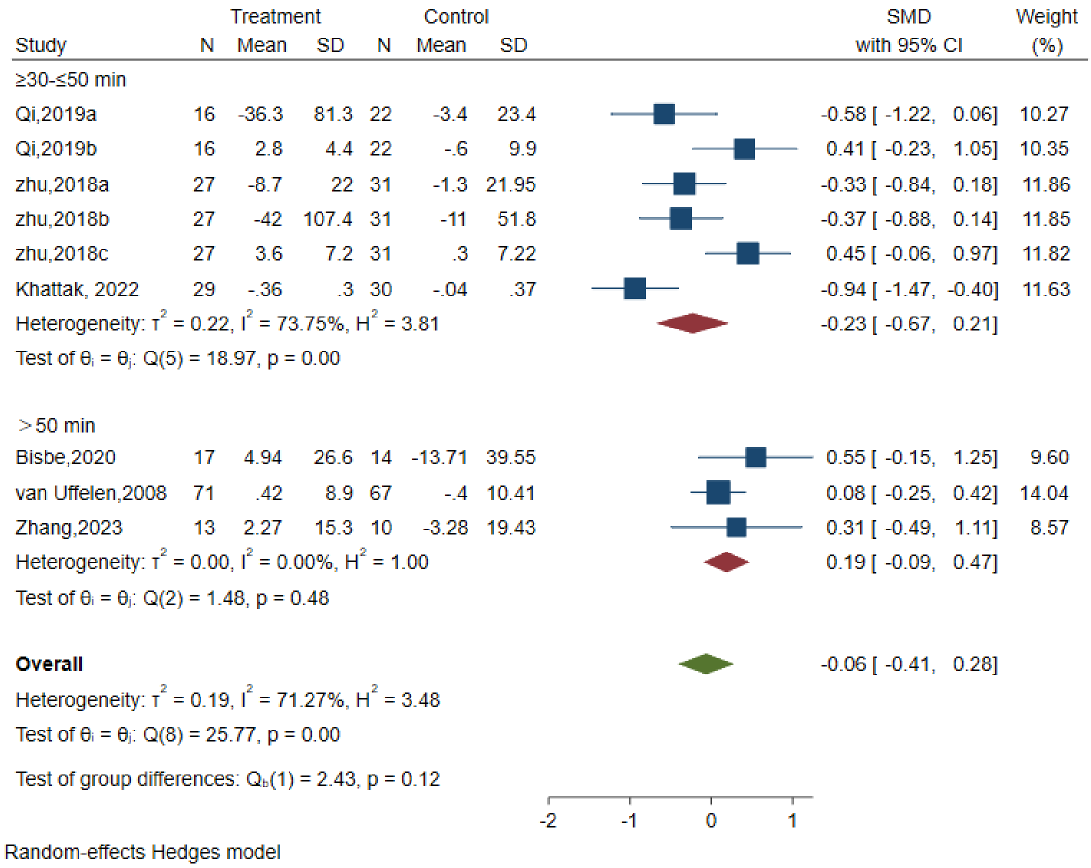

Figure S6B Subgroup analysis of the intervention duration in processing speed

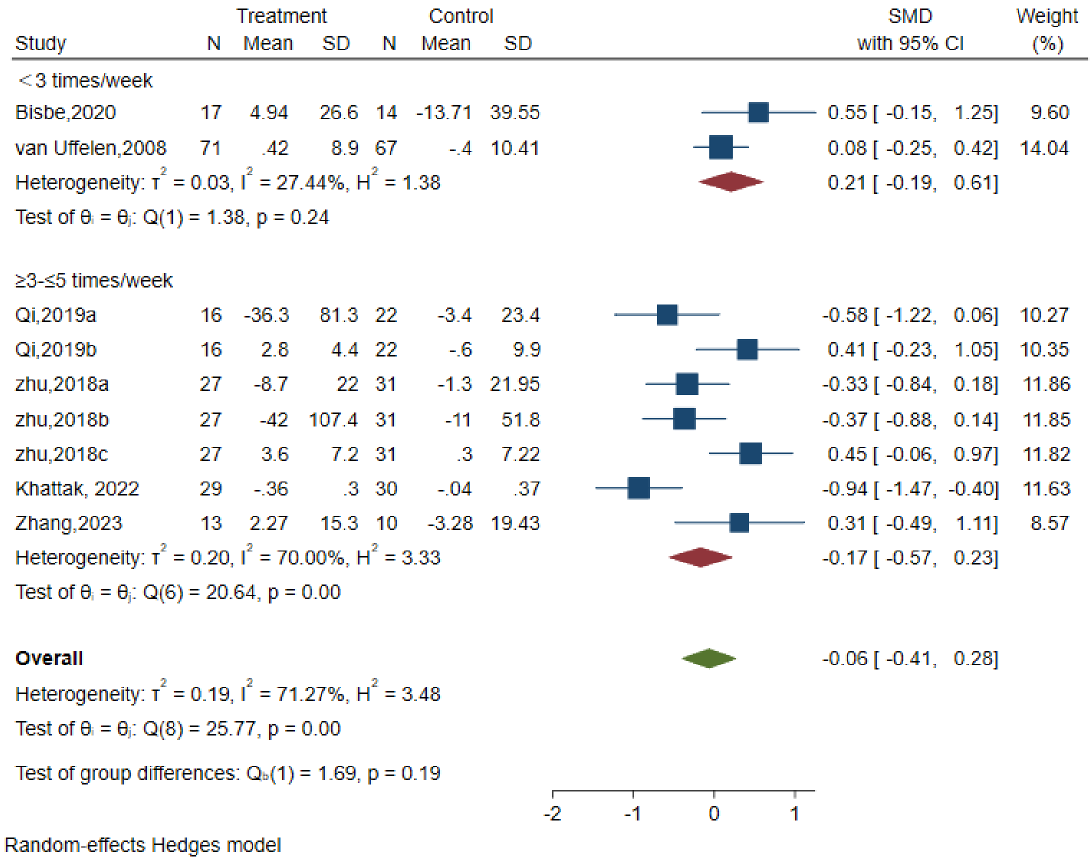

Figure S6C Subgroup analysis of the intervention frequency in processing speed

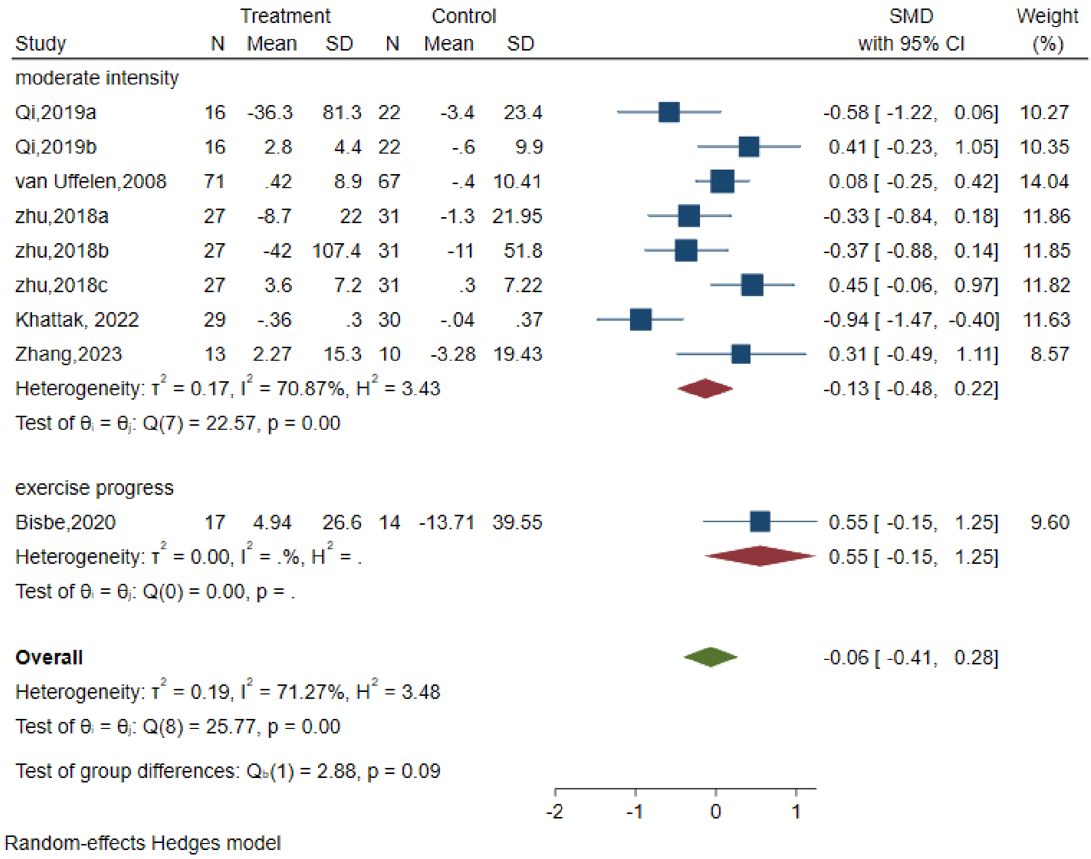

Figure S6D Subgroup analysis of the intervention intensity in processing speed

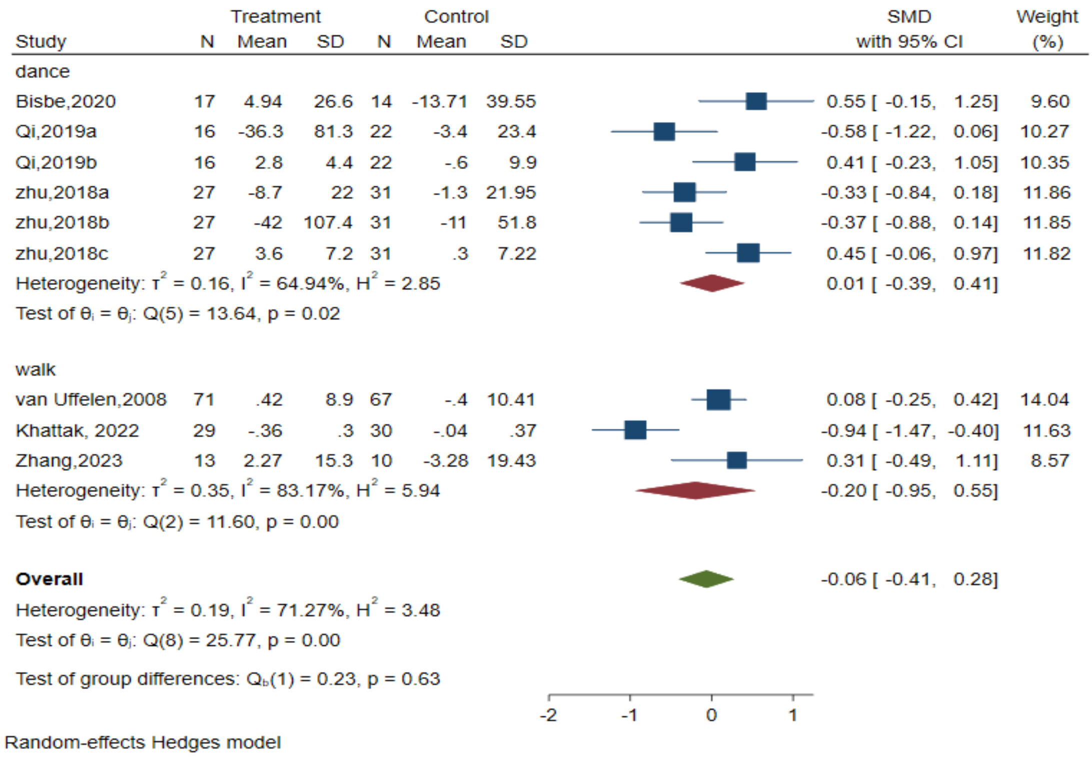

Figure S6E Subgroup analysis of the intervention type in processing speed

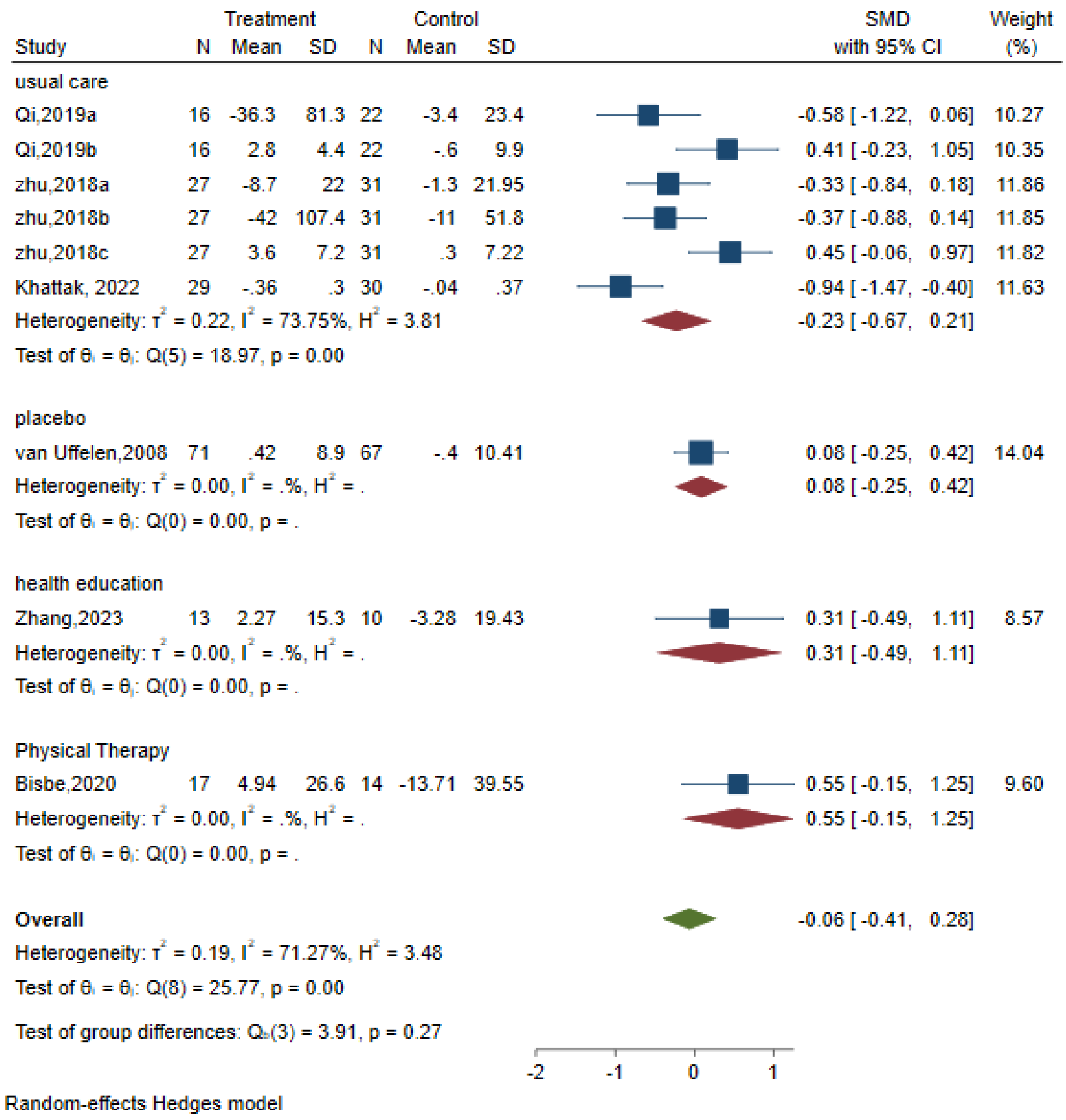

Figure S6F Subgroup analysis of the control type in processing speed

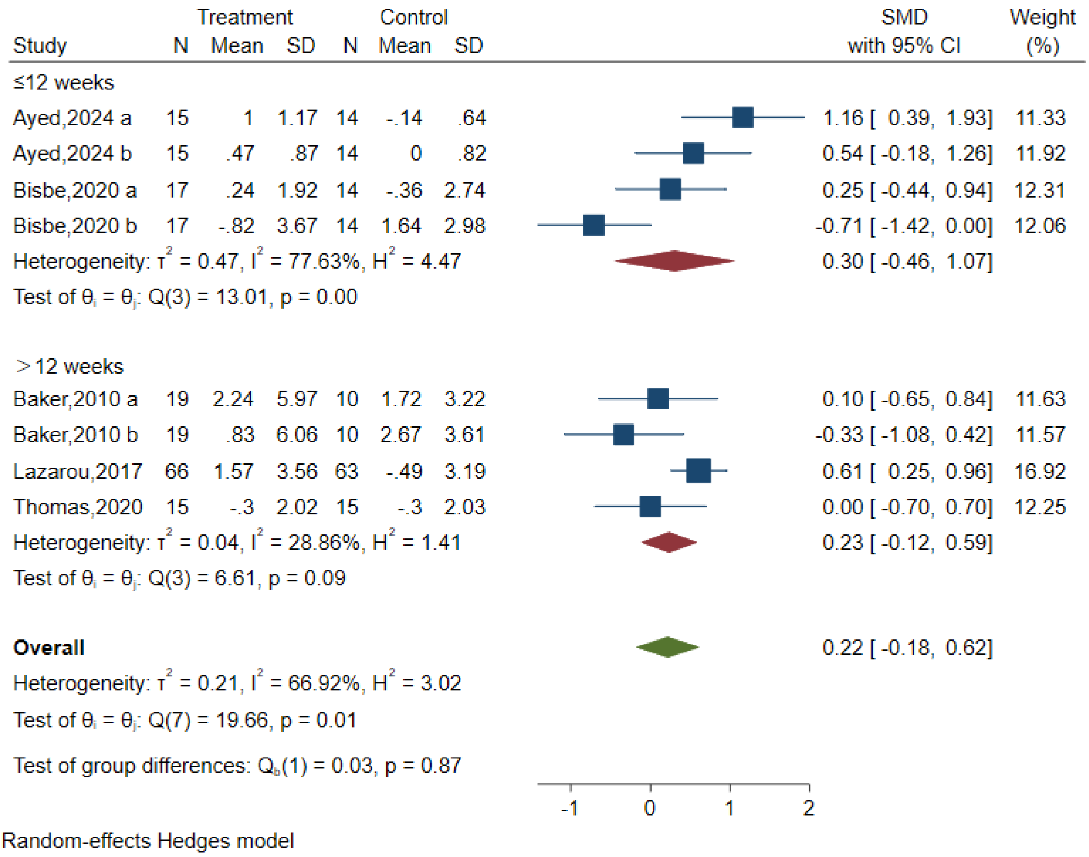

Figure S7A Subgroup analysis of the over length of intervention in language

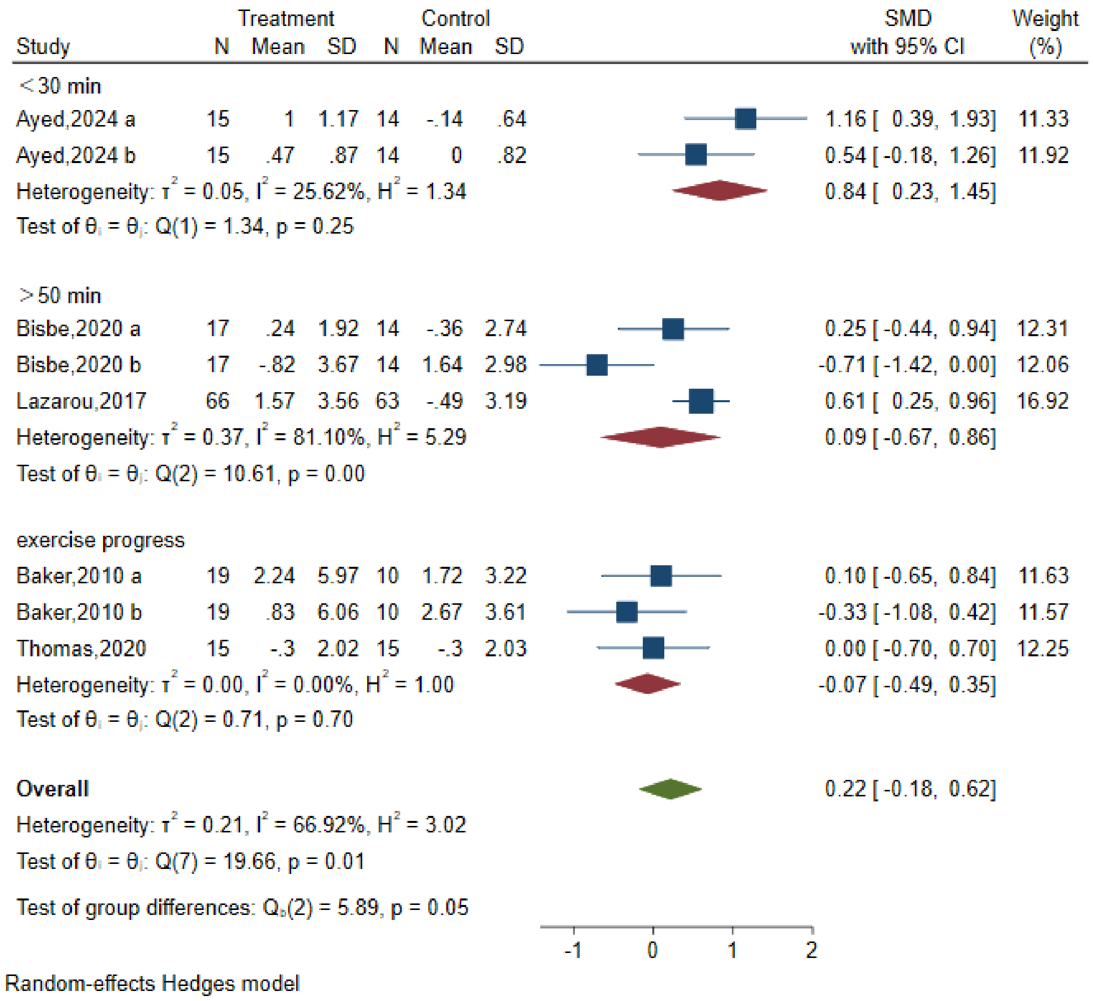

Figure S7B Subgroup analysis of the intervention duration in language

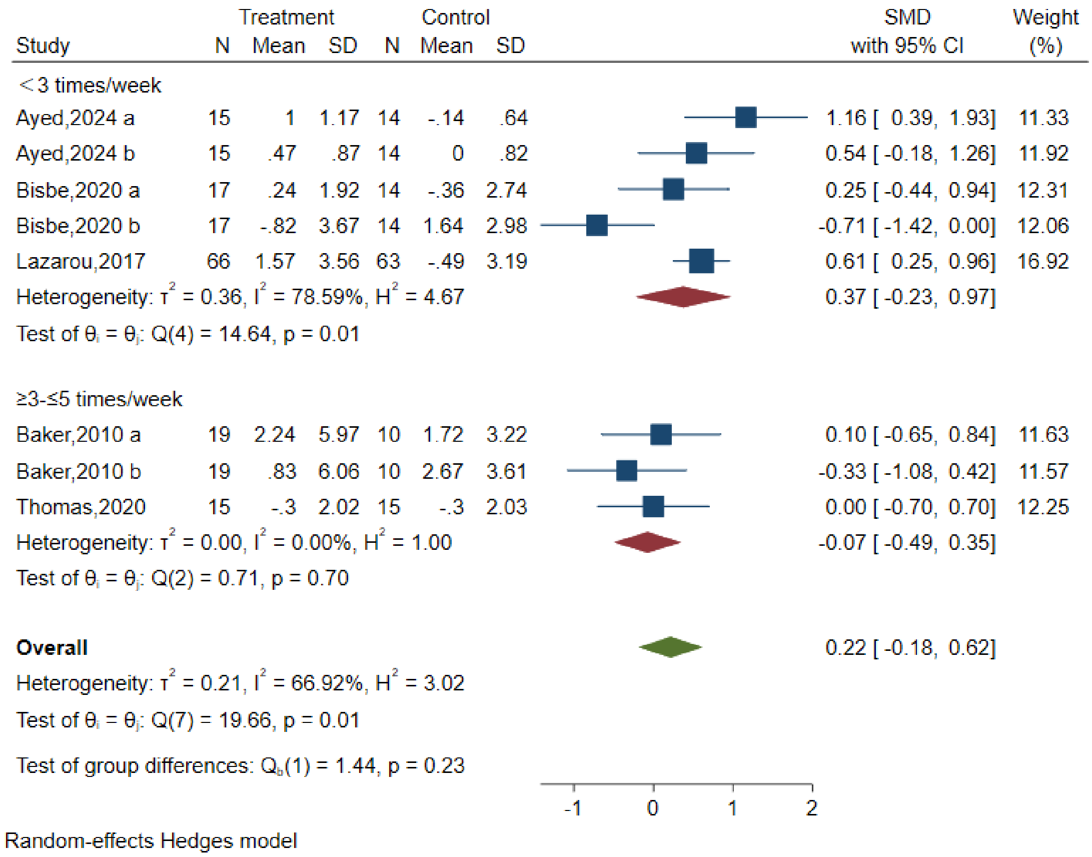

Figure S7C Subgroup analysis of the intervention frequency in language

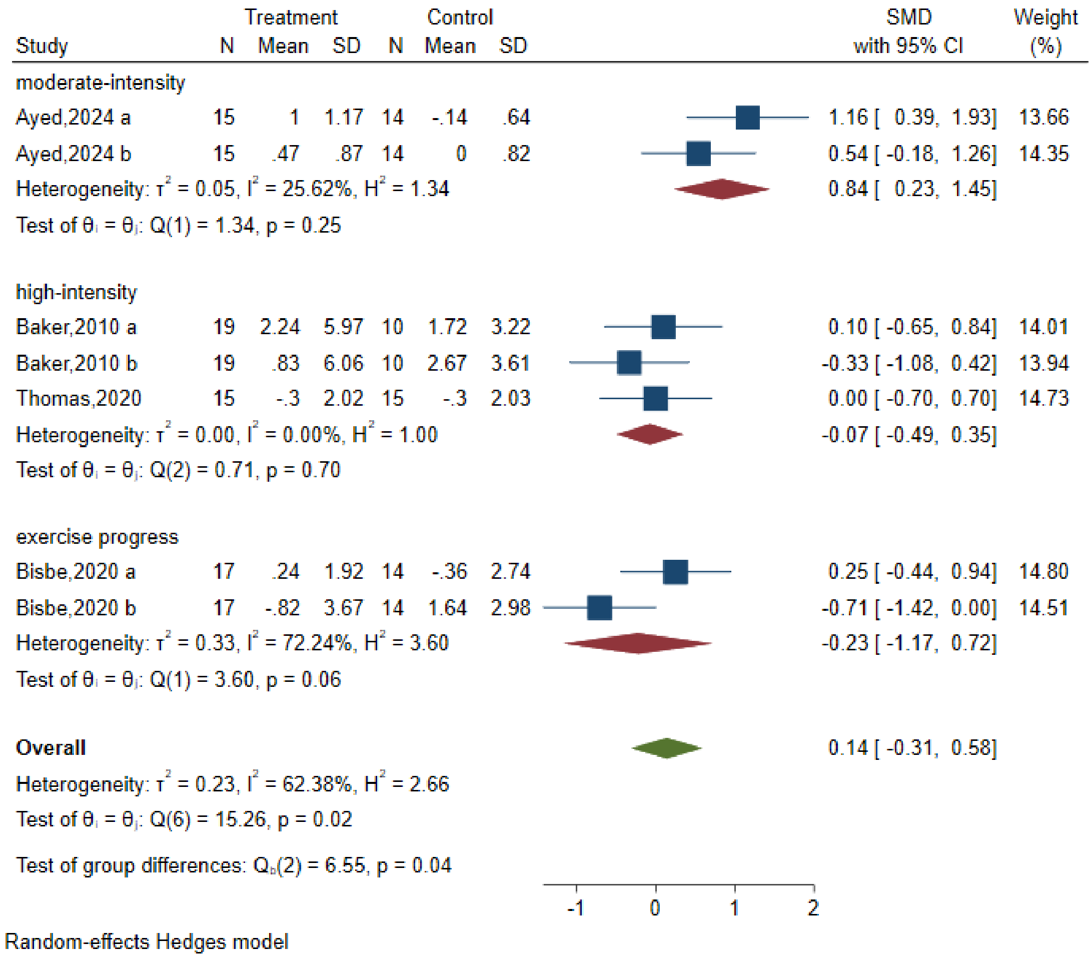

Figure S7D Subgroup analysis of the intervention intensity in language

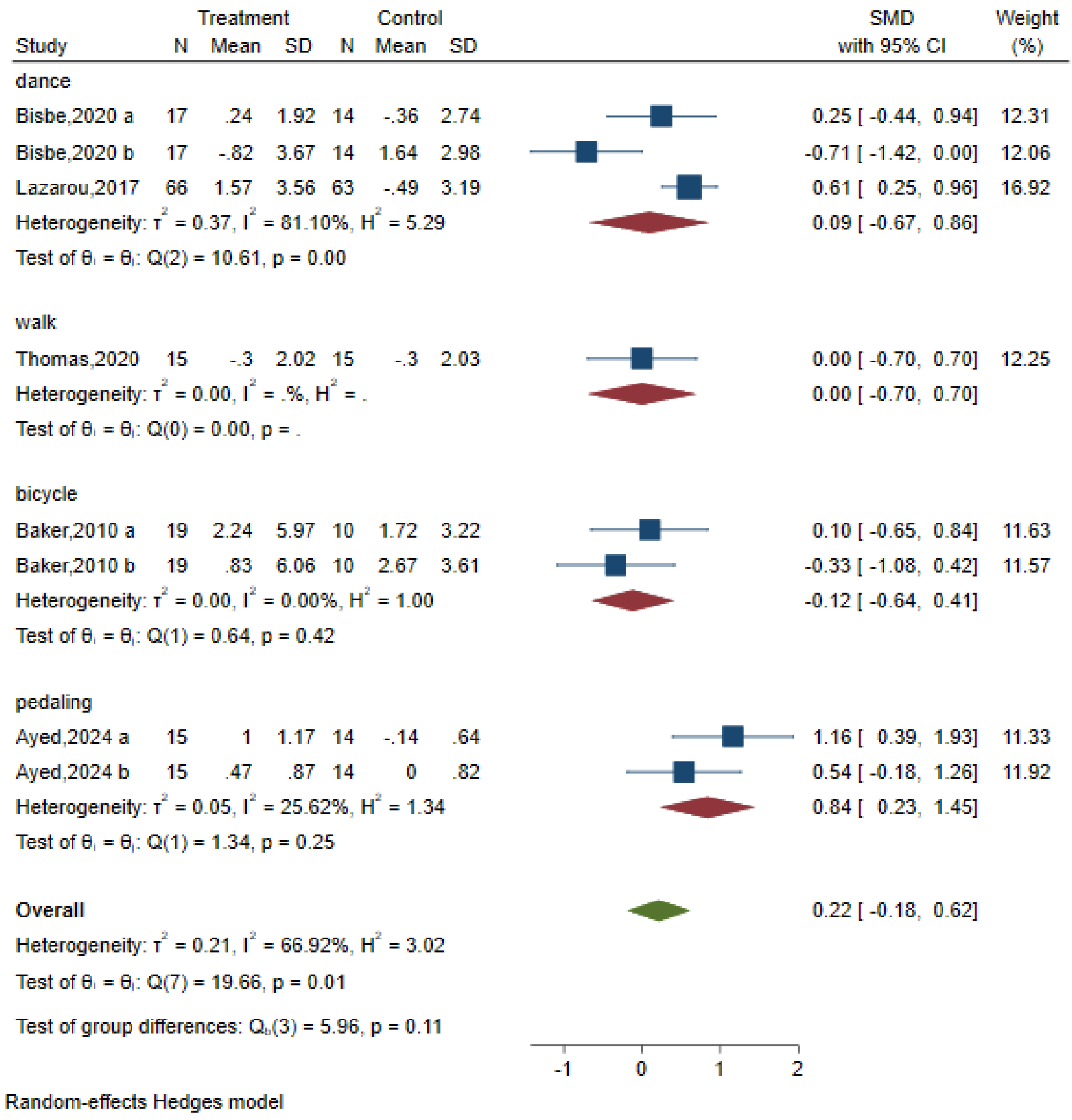

Figure S7E Subgroup analysis of the intervention type in language

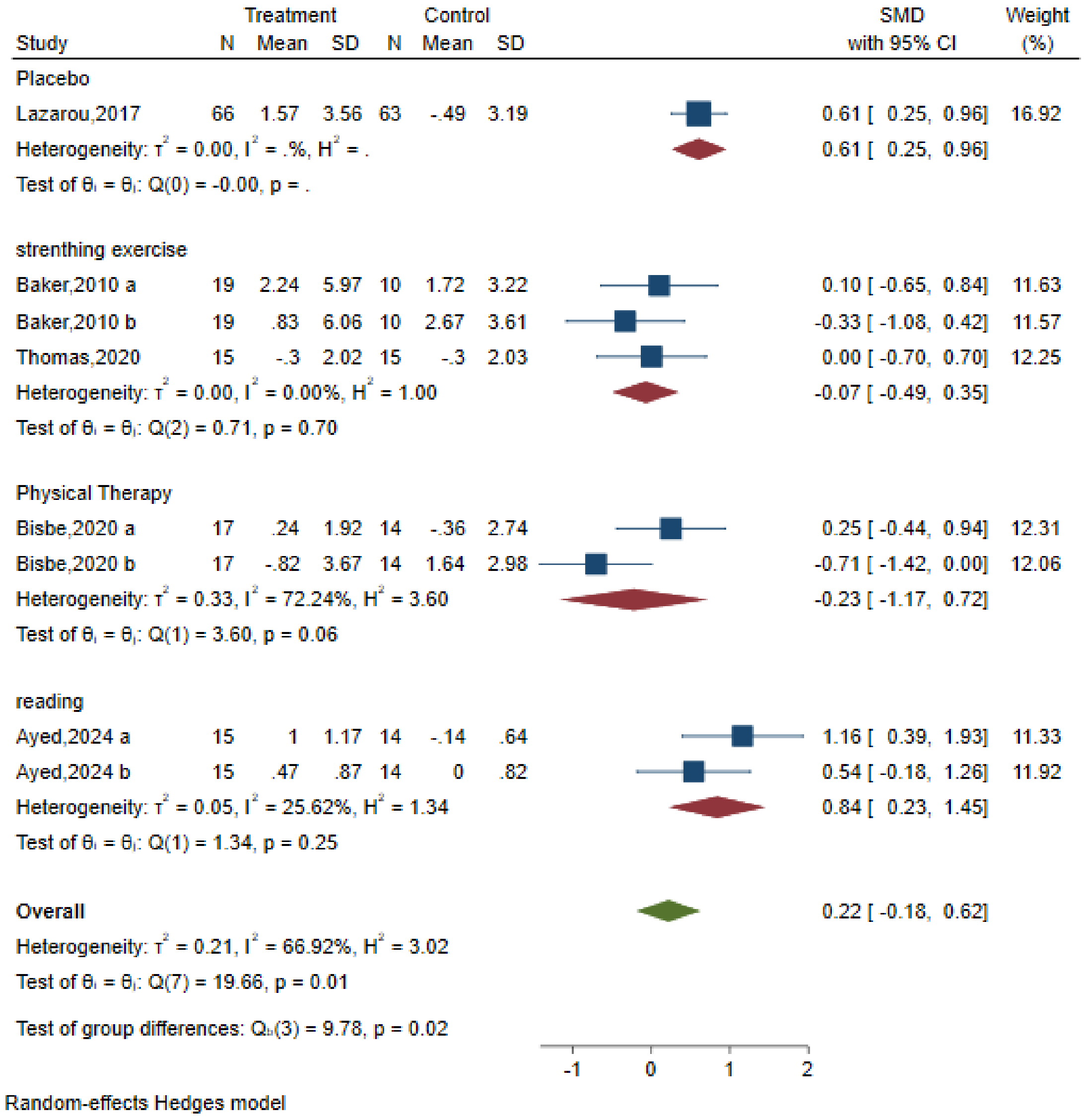

Figure S7F Subgroup analysis of the control type in language

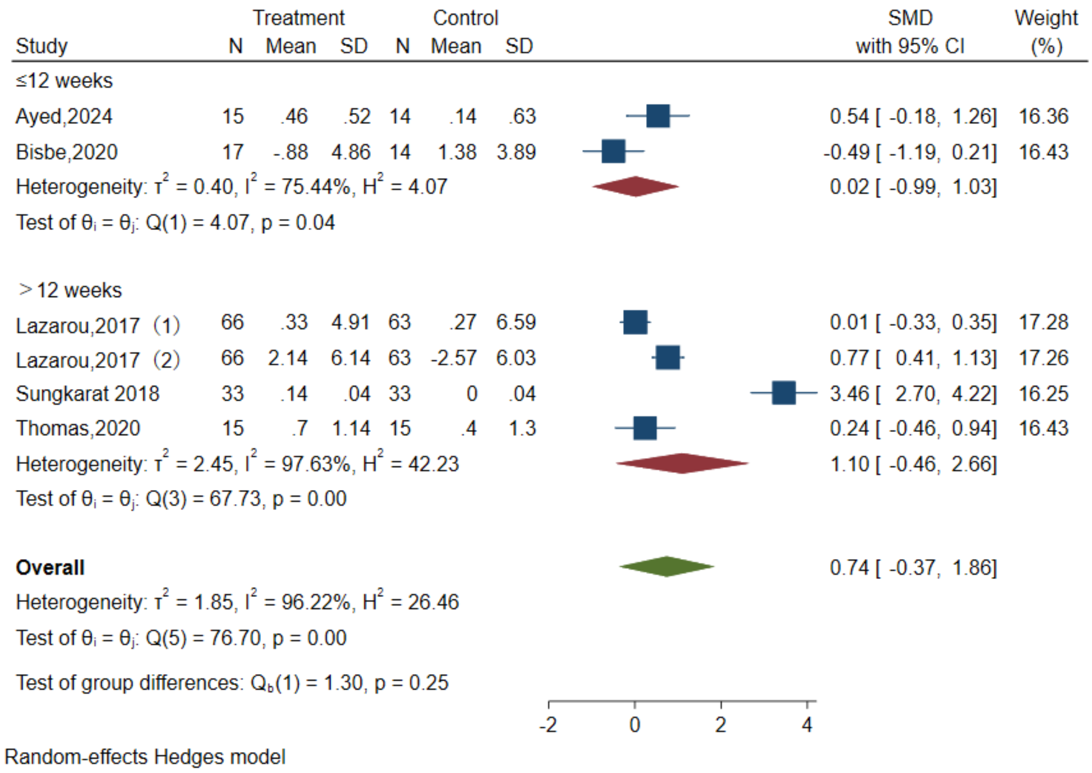

Figure S8A Subgroup analysis of the over length of intervention in Visuospatial function

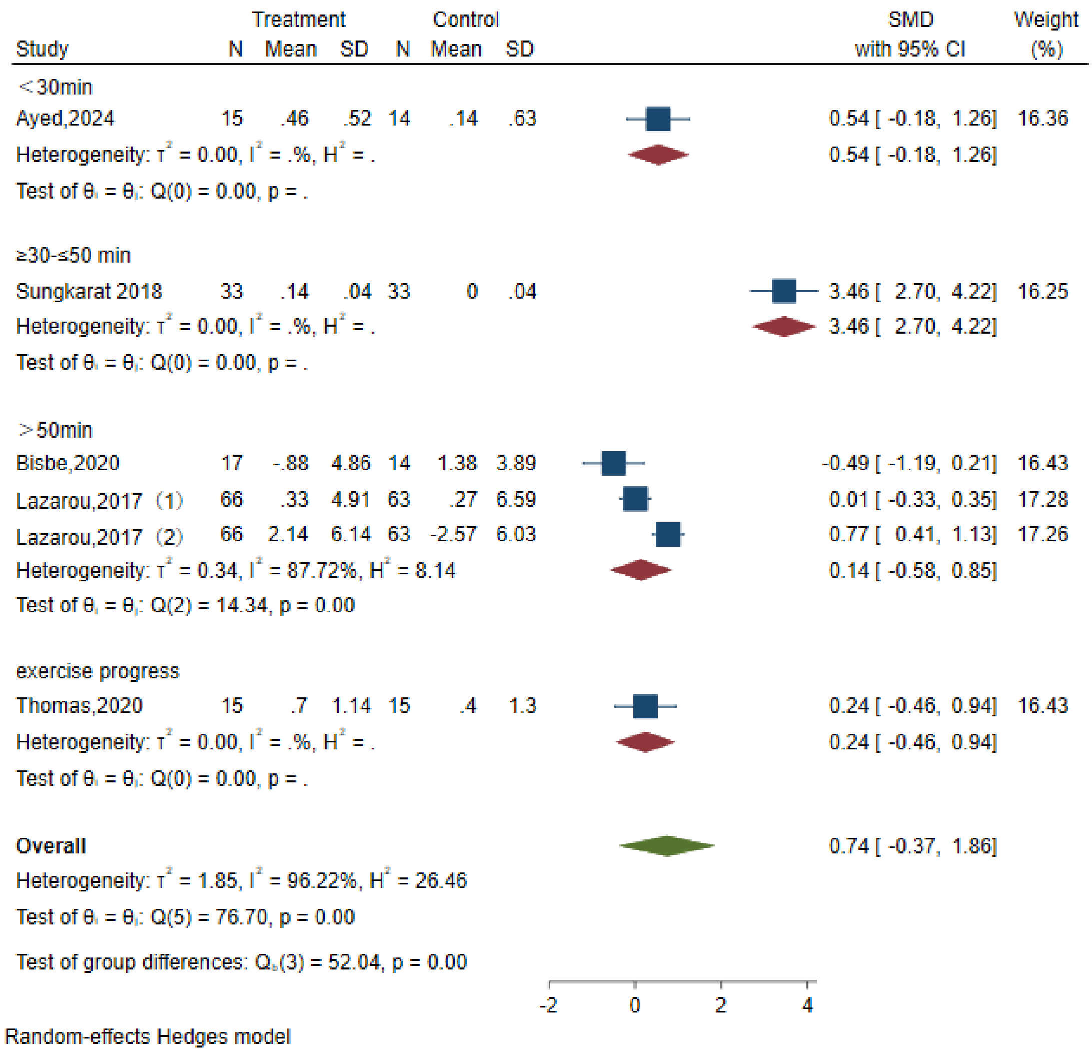

Figure S8B Subgroup analysis of the intervention duration in Visuospatial function

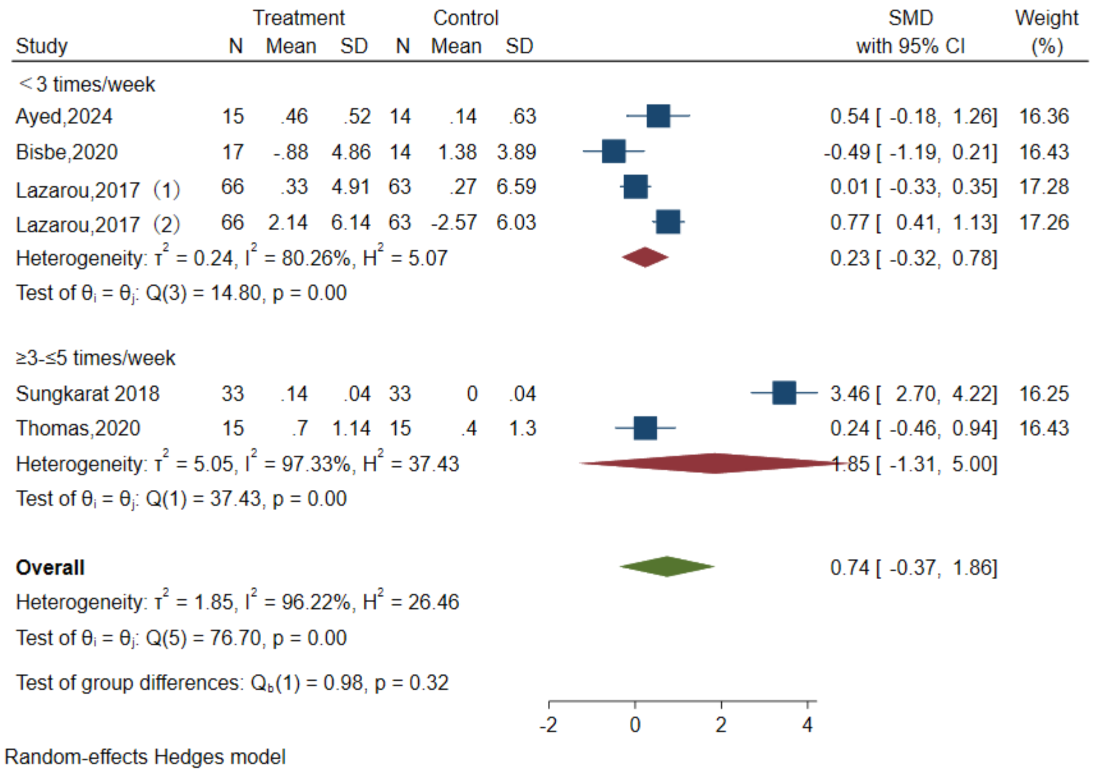

Figure S8C Subgroup analysis of the intervention frequency in Visuospatial function

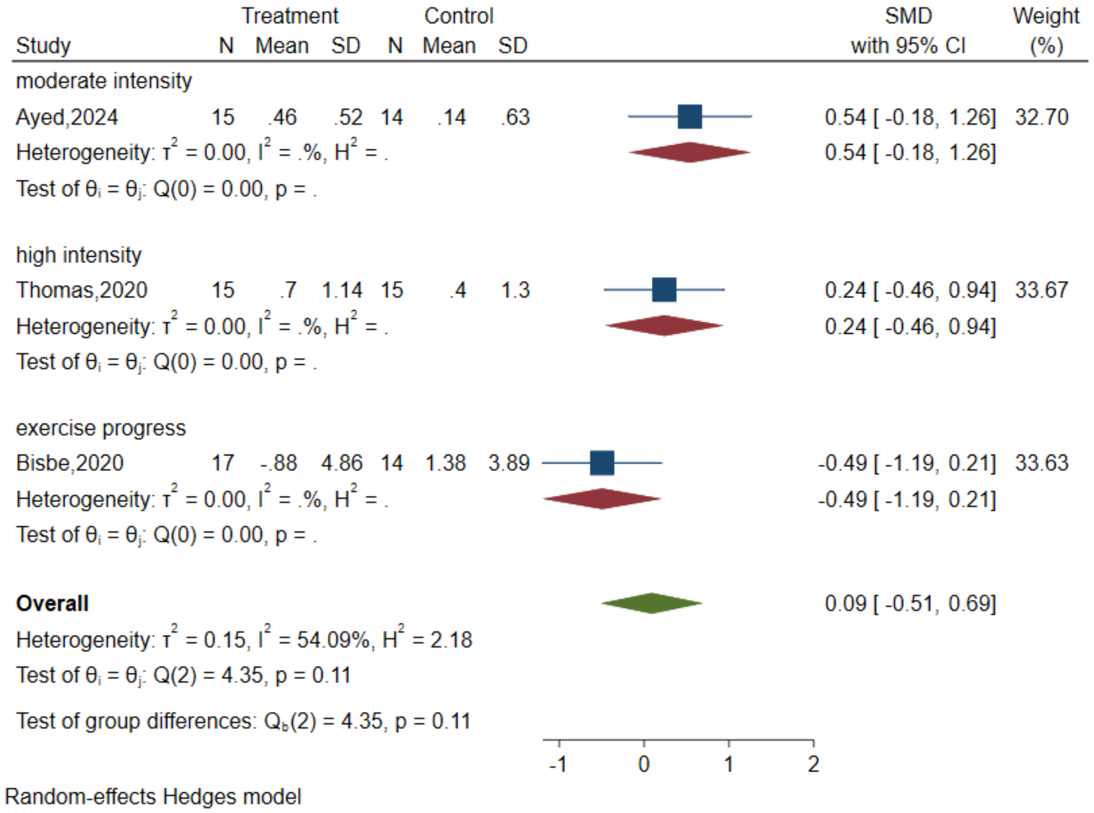

Figure S8D Subgroup analysis of the intervention intensity in Visuospatial function

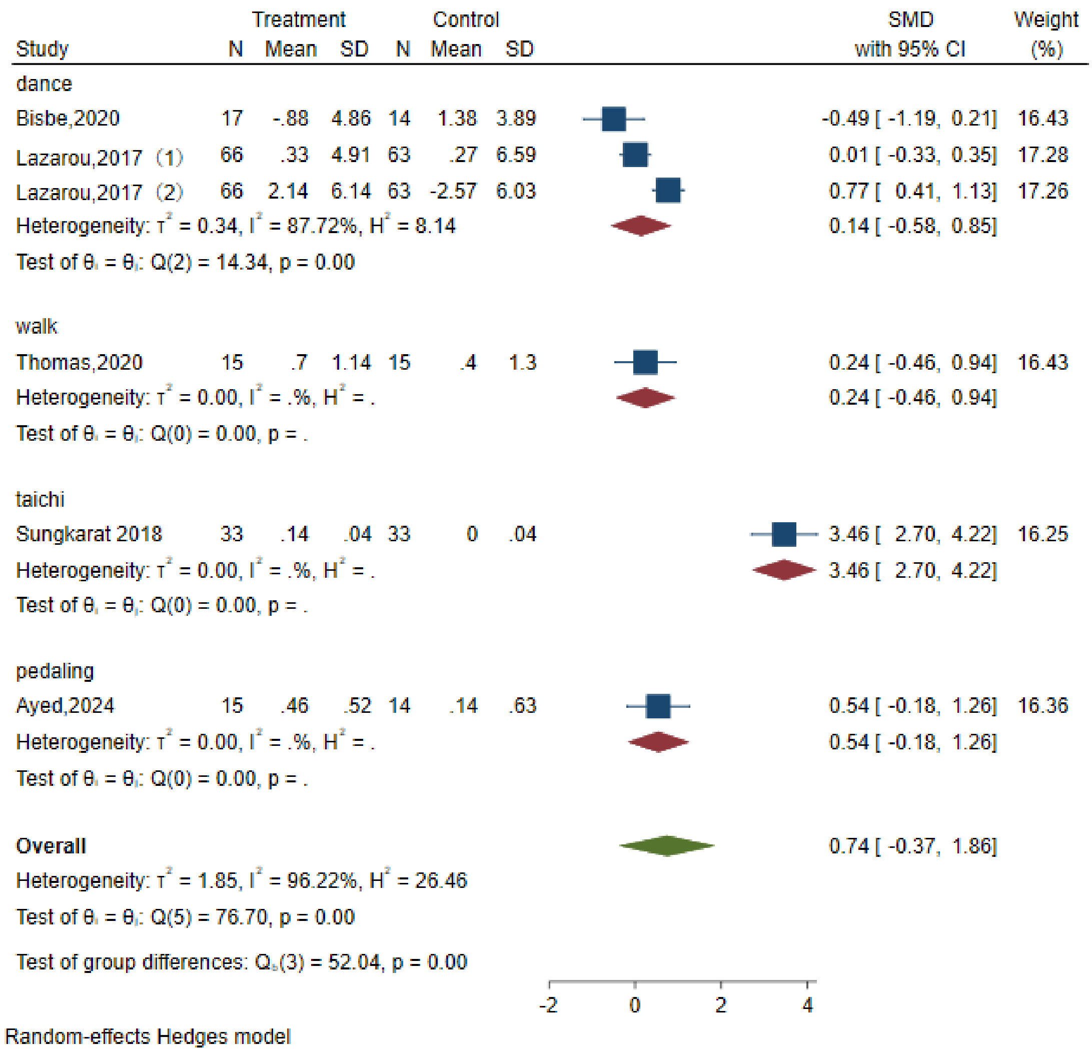

Figure S8E Subgroup analysis of the intervention type in Visuospatial function

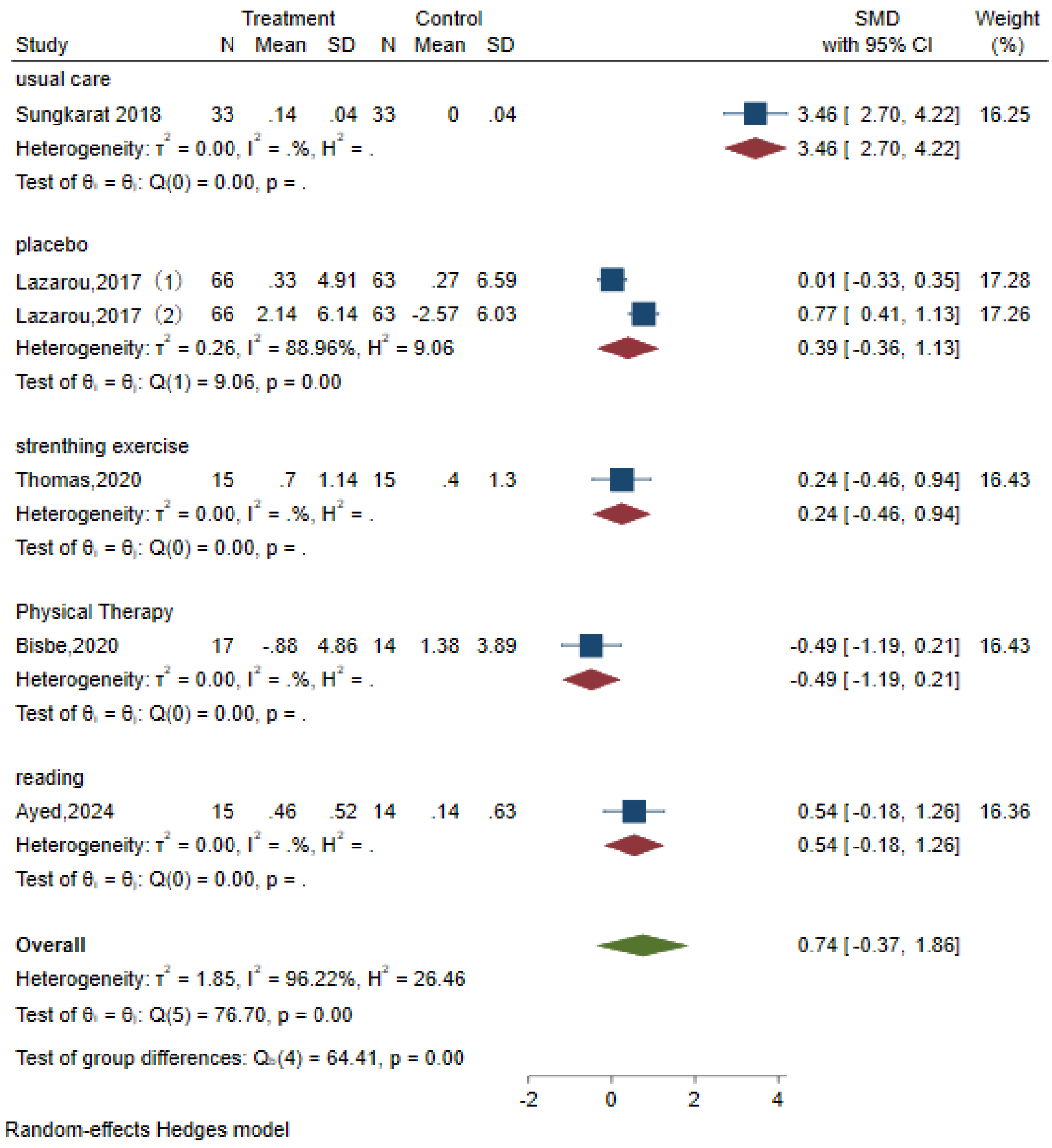

Figure S8F Subgroup analysis of the control type in Visuospatial function

a) global cognition

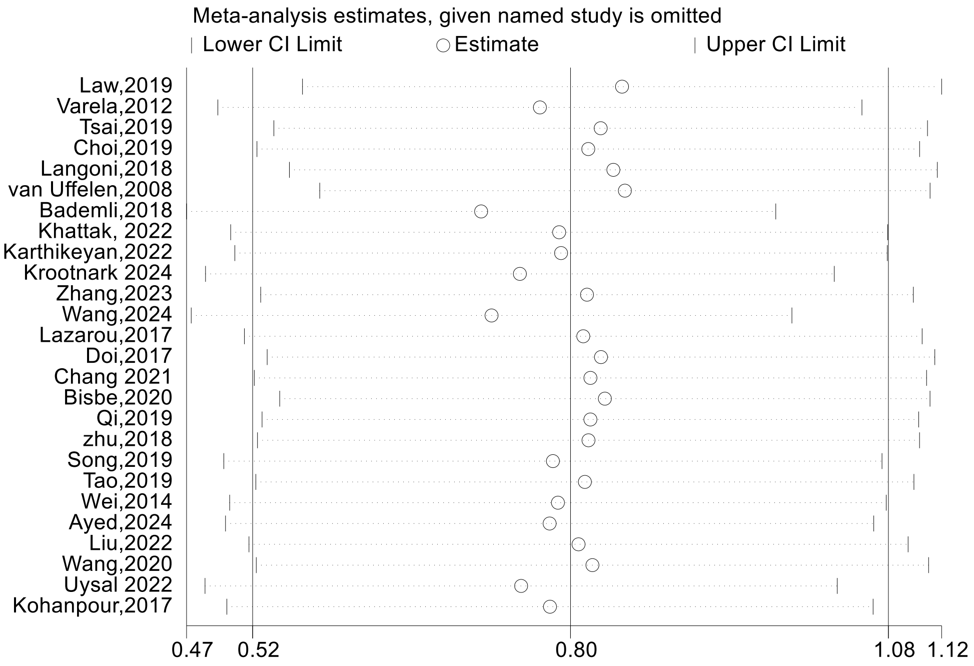

b) executive function

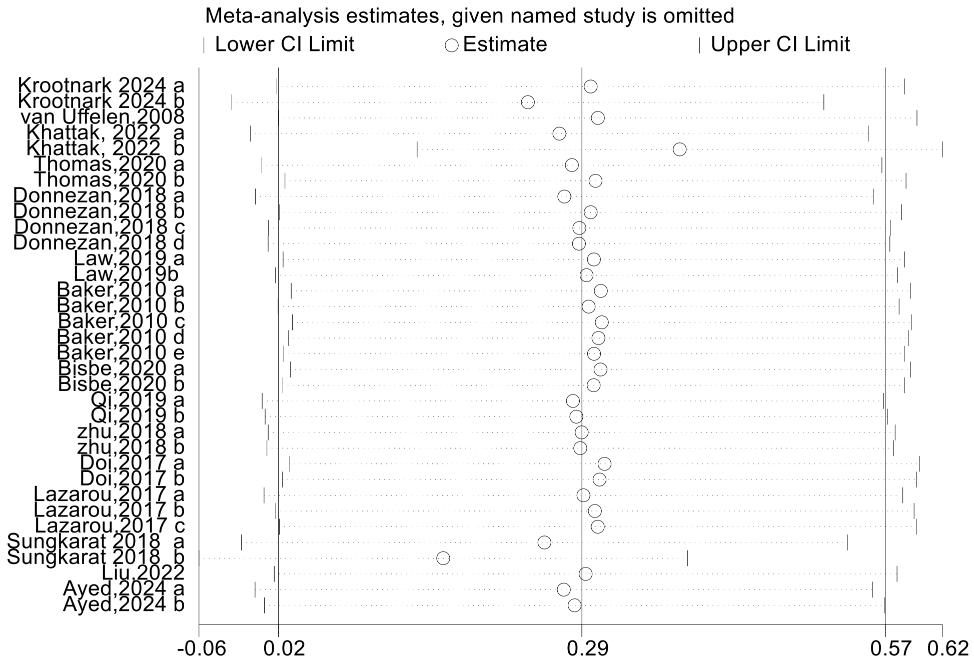

c) memory

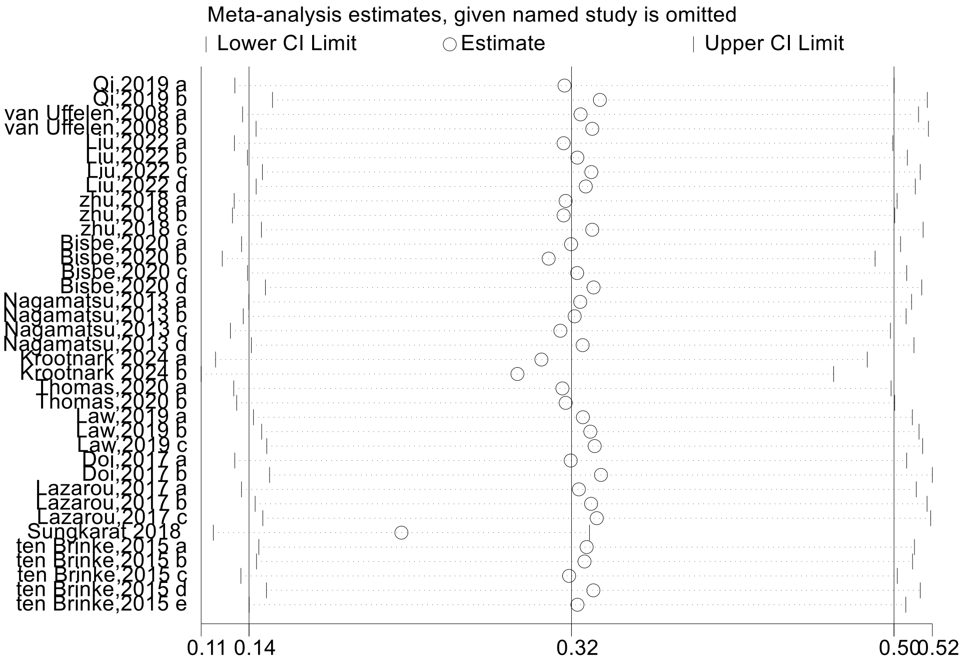

d) verbal memory

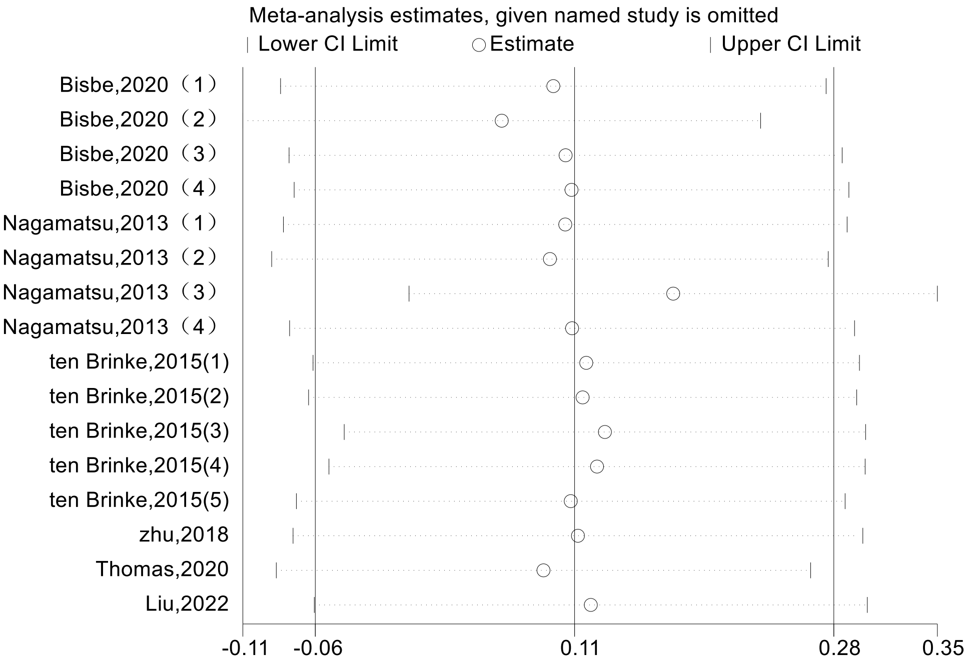

e) attention

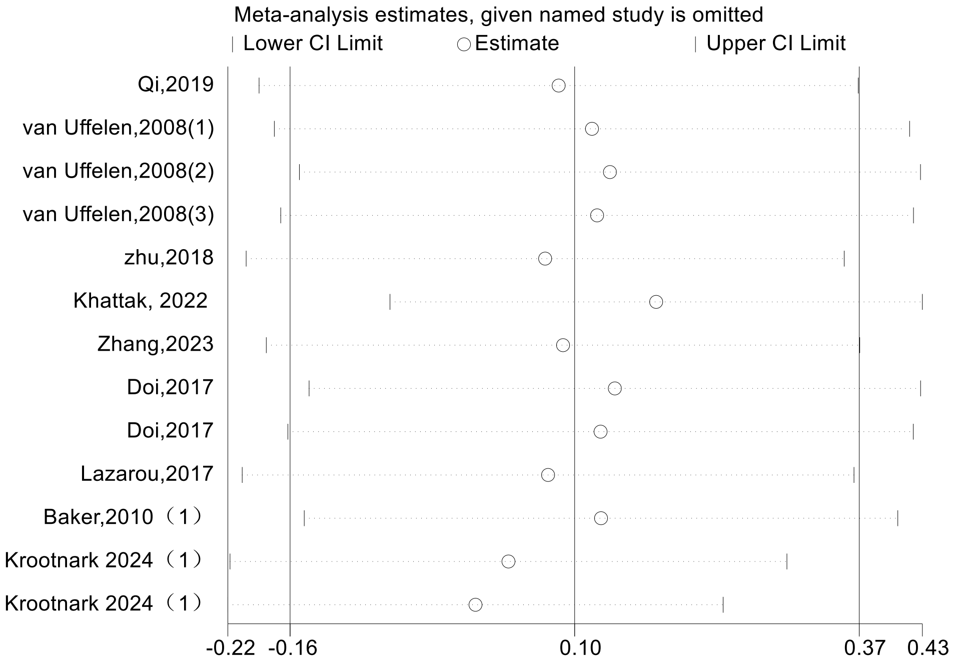

f) processing speed

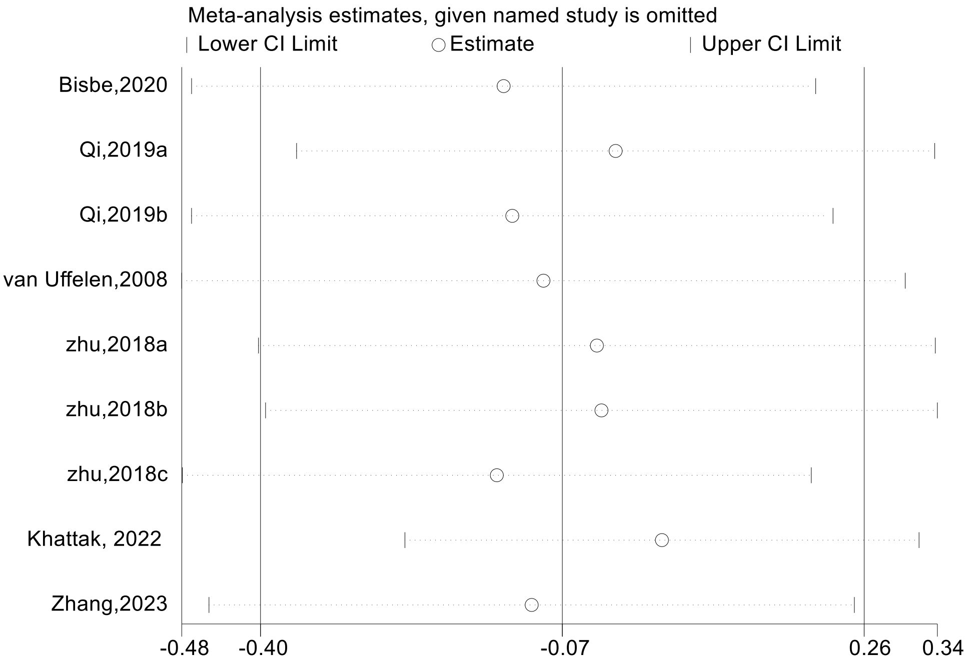

g) language

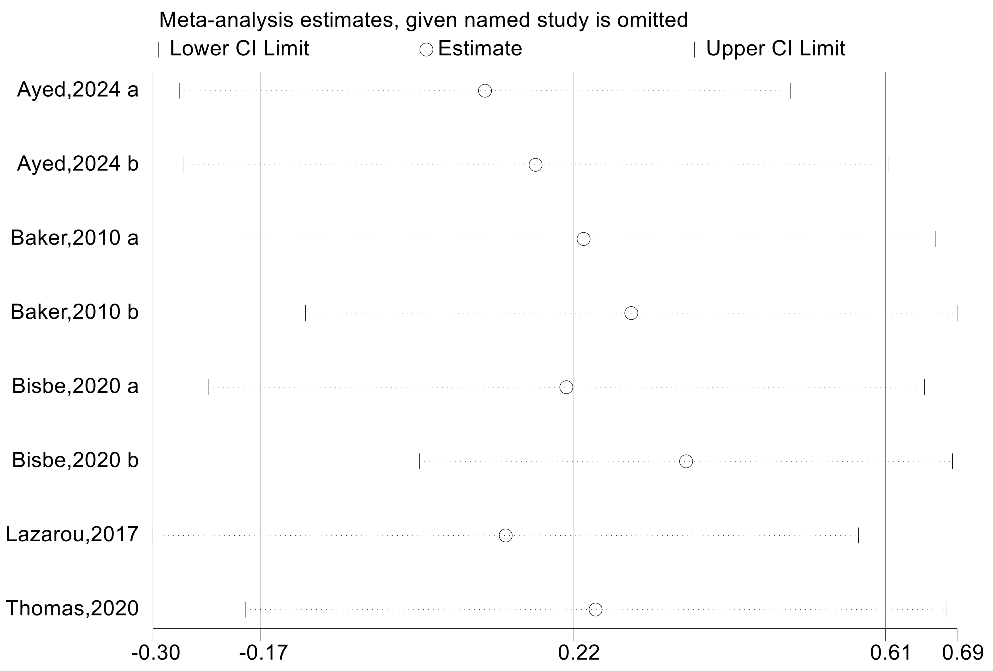

h) visuo-spatial construction

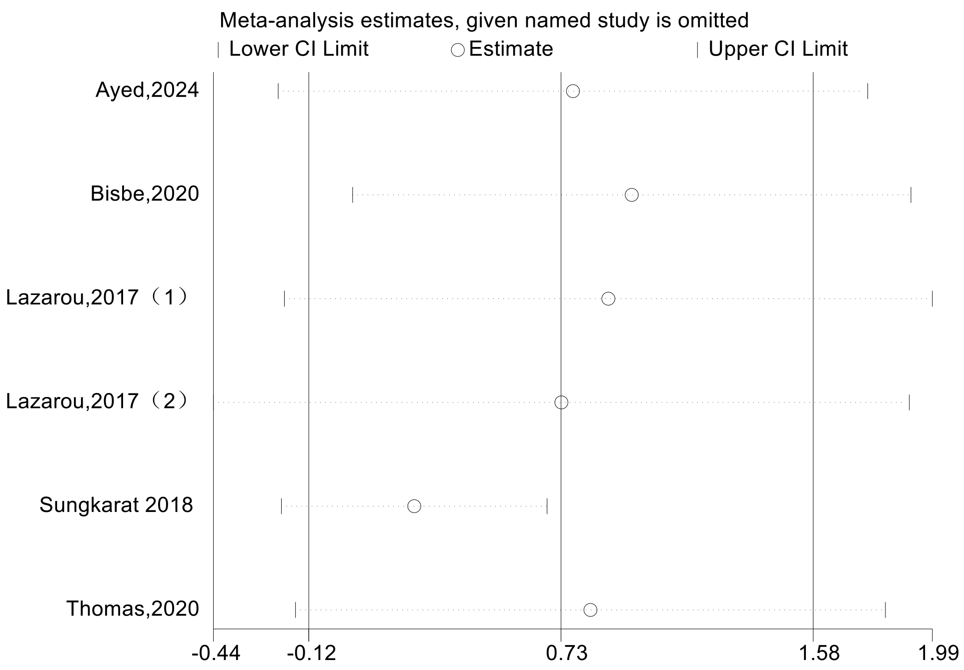

Figure S9 Sensitive analysis of the aerobic exercise on global congnition and different cognitive domains
