# Supplementary material for: Effects of aerobic exercise on cognitive function in older adults with mild cognitive impairment: a systematic review and meta-analysis
Source: Front Psychiatry. 2026 Jan 9;16:1741998. doi: 10.3389/fpsyt.2025.1741998 (PMC12827630; doi:10.3389/fpsyt.2025.1741998)
Supplement: Supplementary file 2 [file DataSheet1.pdf]

Supplement Table 4. The measurement tools for different outcomes

**Cognitive domains**	**Measurement tools of interests**
Global cognition	Mini-Mental State Examination (MMSE); The Montreal cognitive assessment (MoCA); Addenbrooke's Cognitive Examination Revised (ACE-R); The Neurobehavioral Cognitive Status Examination (NCSE)
Executive function	Trial mark test (TMT-B, TMT-A); Stroop color word test (SCWT); Digit span test (backward, forward); verbal fluency test (VFT); the Matrix Reasoning test; Task Switching; Symbol Digit Modalities; Task switching; Letter Fluency , Category Fluency; the Wechsler Adult Intelligence Scale 3rd Edition (WAIS-III); and Rey Osterrieth Complex Figure Test
Memory	Digit span test (backward, forward); Auditory Verbal Learning Test (AVLT); Chinese Version Verbal Learning Test (CVVLT); the Wechsler Memory Scale-Third Edition (WMS-III), the Visual Memory subtest of the Repeatable Battery for the Assessment of Neuropsychological Status (RBANS); the National Center for Geriatrics and Gerontology Functional Assessment Tool study cognitive assessment battery; Rivermead Behavioral Memory Test (RBMT); Rey's Auditory Verbal Learning Test (RAVLT); The Wechsler Memory Scale-Revised Logical Memory (WMS-R LM); International shopping list; list learning delayed recall test and spatial n-back task test
Attention	Trial mark test (TMT-A); Abridged Stroop Colour Word Test (SCWT-A); Digit span test (backward, forward); Attention network test(ANT)； and Test of Everyday Attention (TEA 4)

Processing speed	Digit Symbol Substitution Test (DSST); Symbol Digit Modalities Test (SDMT), The Purdue Pegboard test; and Trail-Making Test (TMT)
Language	Verbal Fluency Test (VFT), Verbal Fluency F-A-S test(FAS), and Boston Naming Test (BNT)

Table S1. Summary of the meta-regression results

**Moderator**	**Test of overall effect**	**Estimate**	**SE**	**z**	***p***
Global cognition					
AE period (weeks)	Q = 163.79, df = 25, p = 0.000	-0.02	0.01	-1.47	0.143
Duration (minutes)	*Q = 101.39, df = 19, p = 0.000*	*-0.03*	*0.01*	*-2.46*	*0.01*
Frequency (number of sessions/week)	Q = 150.26, df = 23, p = 0. 000	0.25	0.13	1.92	0.06
Intervention Intensity	Q = 137.95, df = 21, p = 0. 000	-0.03	0.22	-0.15	0.88
Intervention type	Q = 152.43, df = 23, p = 0. 000	0.01	0.08	0.17	0.86
Control type	Q = 130.59, df = 23, p = 0. 000	0.00	0.10	0.04	0.97
Scale type	Q = 163.79, df = 25, p = 0. 000	-0.09	0.28	-0.33	0.74
Attention					
AE period (weeks)	Q = 54.40, df = 12, p = 0.000	-0.01	0.01	-1.13	0.26

Duration (minutes)	Q = 49.45, df = 10, p = 0.000	-0.01	0.25	-0.04	0.96
Frequency (number of sessions/week)	Q = 46.98, df = 9 p = 0.000	0.10	0.15	0.66	0.51
Intervention Intensity	*Q = 21.57, df = 9, p = 0.01*	*-0.83*	*0.29*	*-2.89*	*0.00*
Intervention type	Q = 54.40, df = 12, p = 0.000	0.01	0.17	0.03	0.97
Control type	Q = 54.40, df = 12, p = 0.000	-0.23	0.16	-1.43	0.15
memory					
AE period (weeks)	Q = 134.45, df = 36, p = 0.000	-0.00	0.01	-0.18	0.86
Duration (minutes)	Q = 114.71, df = 32, p = 0.000	-0.01	0.02	-0.58	0.56
Frequency (number of sessions/week)	Q =126.1 7, df = 2 9, p = 0.000	0.33	0.21	1.53	0.13
Intervention Intensity	Q = 36.8 3, df = 3 0, p = 0.182	-0.07	0.06	-1.23	0.21
Intervention type	Q =132.18, df = 31, p =0.000	0.23	0.14	1.61	0.11
Control type	Q = 134.45, df = 36, p = 0.000	-0.07	0.08	-0.90	0.37
Executive function					
AE period (weeks)	Q =262.45, df = 33, p =0.000	-0.00	0.02	-0.03	0.97
Duration (minutes)	Q = 145.29, df = 22, p = 0.000	-0.02	0.02	-0.76	0.45
Frequency (number of sessions/week)	Q = 249.08, df = 27, p = 0.000	-0.16	0.26	-0.60	0.55
Intervention Intensity	Q =132.65, df =26, p =0.000	-0.3	0.23	-1.32	0.19

Intervention type	Q =262.45, df = 33, p =0.000	0.32	0.19	1.66	0.10
Control type	Q = 262.45, df = 33, p =0.000	-0.15	0.15	-1.02	0.31

Note. SE means Standard Error
